# Supplementary material for: Caspase cleavage of influenza A virus M2 disrupts M2-LC3 interaction and regulates virion production
Source: EMBO Rep. 2025 Mar 3;26(7):1768–91. doi: 10.1038/s44319-025-00388-7 (PMC11977235; doi:10.1038/s44319-025-00388-7)
Supplement: Supplementary file 1 — Table EV1 [file 44319_2025_388_MOESM1_ESM.docx]

**Table EV1**

Crystallisation data collection and refinement statistics.

Values in parentheses are for the highest resolution shell.

| **Data collection statistics** |  |
| --- | --- |
| Beamline | SLS PX III |
| Wavelength (Å) | 1.006 |
| Space Group | *P2_1_2_1_2_1_* |
| Unit Cell (Å) | *a* = 35.68, *b* = 38.46, *c* = 93.77  *α* =90.00, *β* = 90.00, *γ* = 90.00 |
| Resolution (Å) | 46.89 - 1.75  (1.84 - 1.75) |
| Observed reflections | 174941 (26026) |
| Unique reflections | 13649 (1935) |
| Redundancy | 12.8 (13.5) |
| Completeness (%) | 100.0 (100.0) |
| *R*_merge_ | 0.051 (0.762) |
| CC1/2 | 1.000 (0.920) |
| <I/σI> | 27.9 (3.3) |
| **Refinement statistics** |  |
| Reflections in test set | 666 |
| *R*_cryst_ | 22.0 |
| *R*_free_ | 24.9 |
| **Number of groups** |  |
| Protein residues | 129 |
| Ions and ligand atoms | 0 |
| Water | 20 |
| Wilson B-factor | 27.36 |
| **RMSD from ideal geometry** |  |
| Bond length (Å) | 0.003 |
| Bond angles (°) | 0.536 |
| **Ramachandran Plot Statistics** |  |
| In Favoured Regions (%) | 116 (100.00) |
| In Allowed Regions (%) | 0 (0.00) |
| Outliers (%) | 0 (0.00) |
