# Supplementary figures and images for: Caspase cleavage of influenza A virus M2 disrupts M2-LC3 interaction and regulates virion production
Source: EMBO Rep. 2025 Mar 3;26(7):1768–91. doi: 10.1038/s44319-025-00388-7 (PMC11977235; doi:10.1038/s44319-025-00388-7)

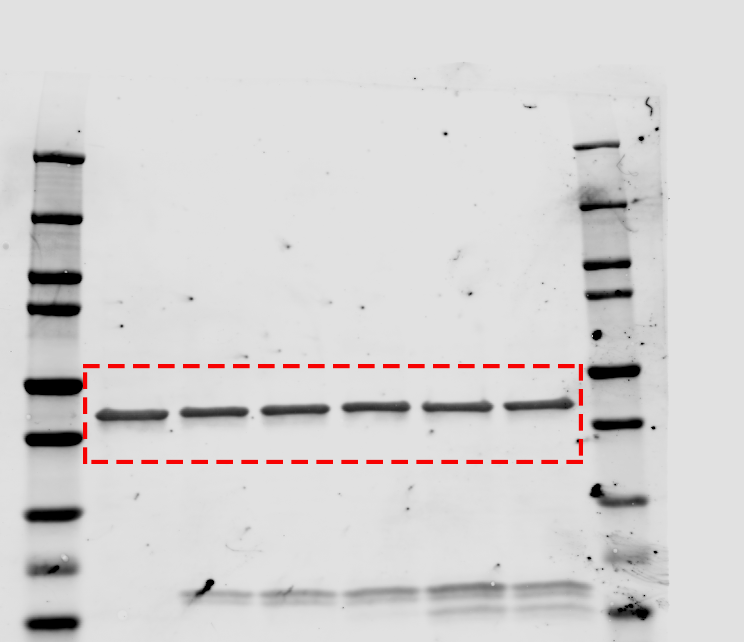

Supplement: Supplementary file 3 — Source data Fig. 1 [file 44319_2025_388_MOESM3_ESM.zip › Figure 1/1A_actin_ms700.tif]

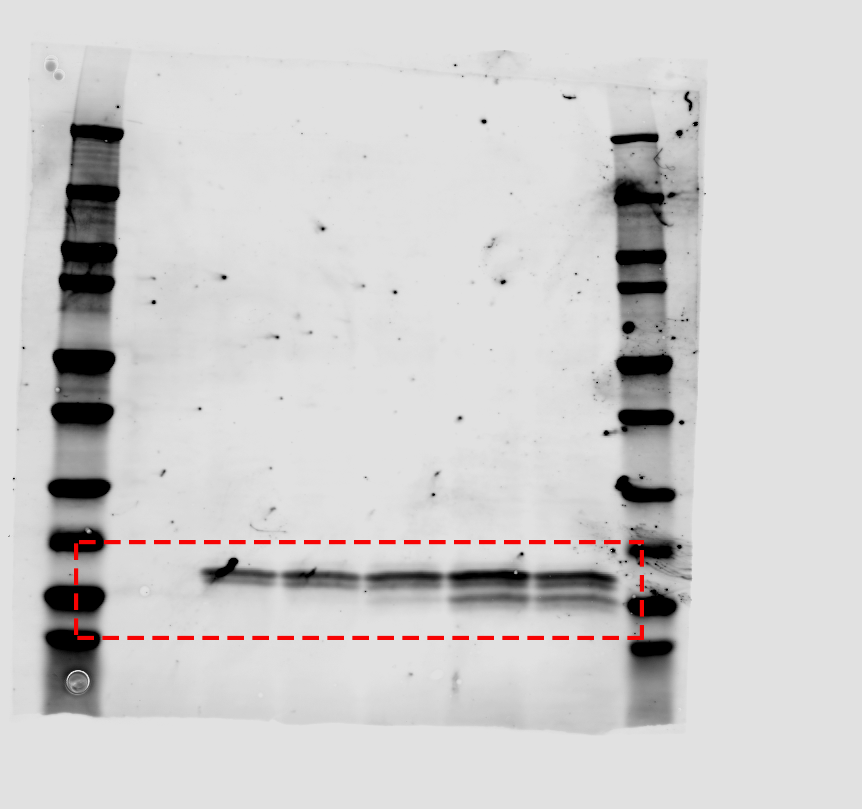

Supplement: Supplementary file 3 — Source data Fig. 1 [file 44319_2025_388_MOESM3_ESM.zip › Figure 1/1A_M2_ms700.tif]

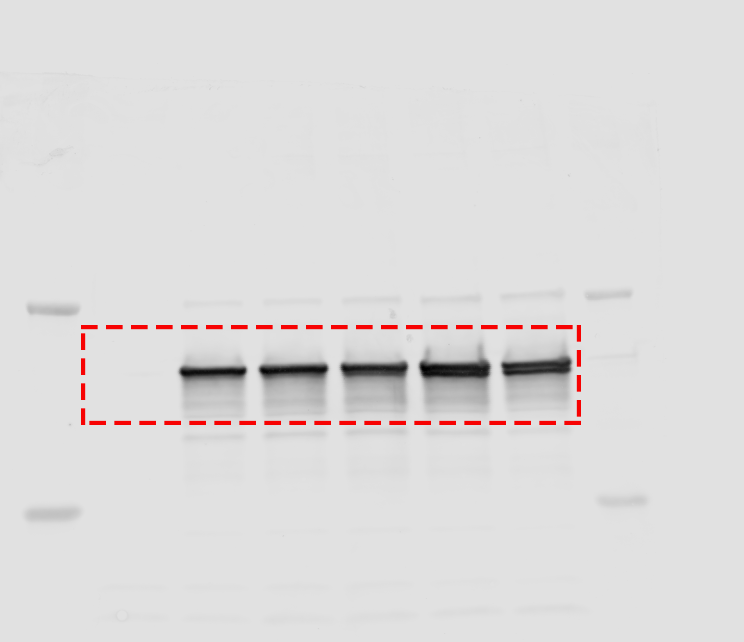

Supplement: Supplementary file 3 — Source data Fig. 1 [file 44319_2025_388_MOESM3_ESM.zip › Figure 1/1A_NP_rb800.tif]

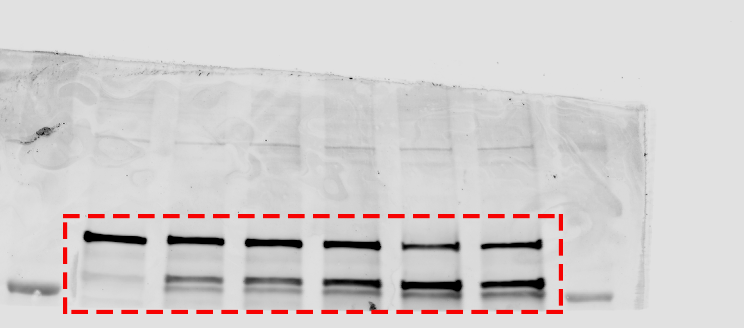

Supplement: Supplementary file 3 — Source data Fig. 1 [file 44319_2025_388_MOESM3_ESM.zip › Figure 1/1A_PARP1_rb800.tif]

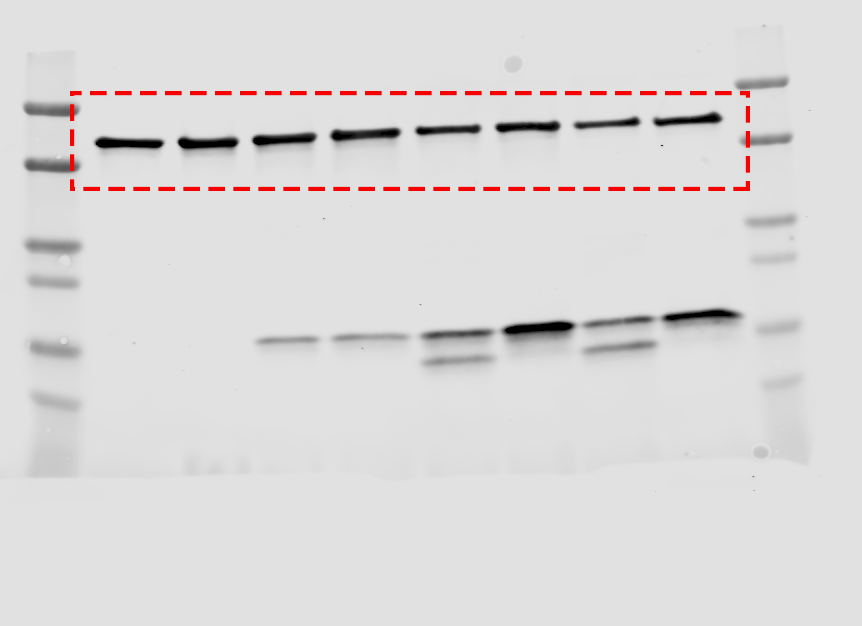

Supplement: Supplementary file 3 — Source data Fig. 1 [file 44319_2025_388_MOESM3_ESM.zip › Figure 1/1B_actin_ms700.tif]

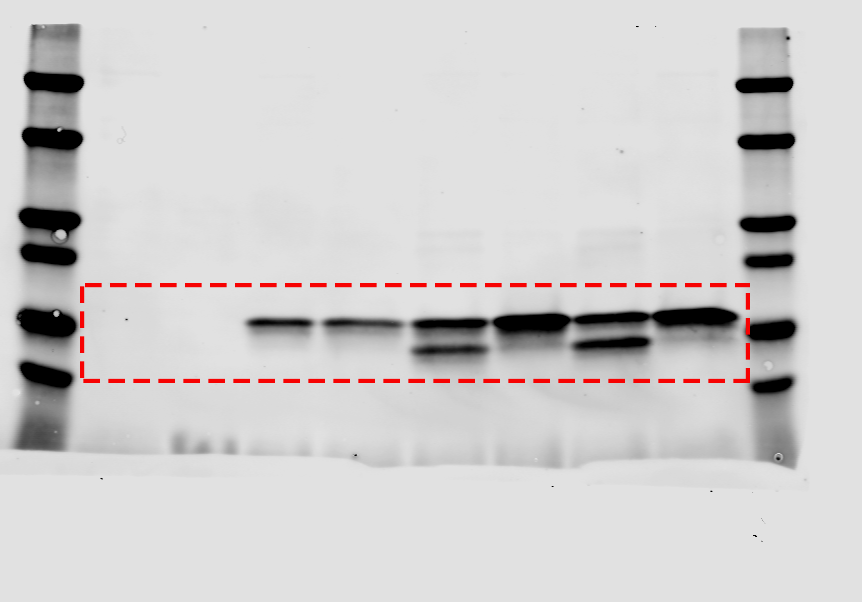

Supplement: Supplementary file 3 — Source data Fig. 1 [file 44319_2025_388_MOESM3_ESM.zip › Figure 1/1B_M2_ms700.tif]

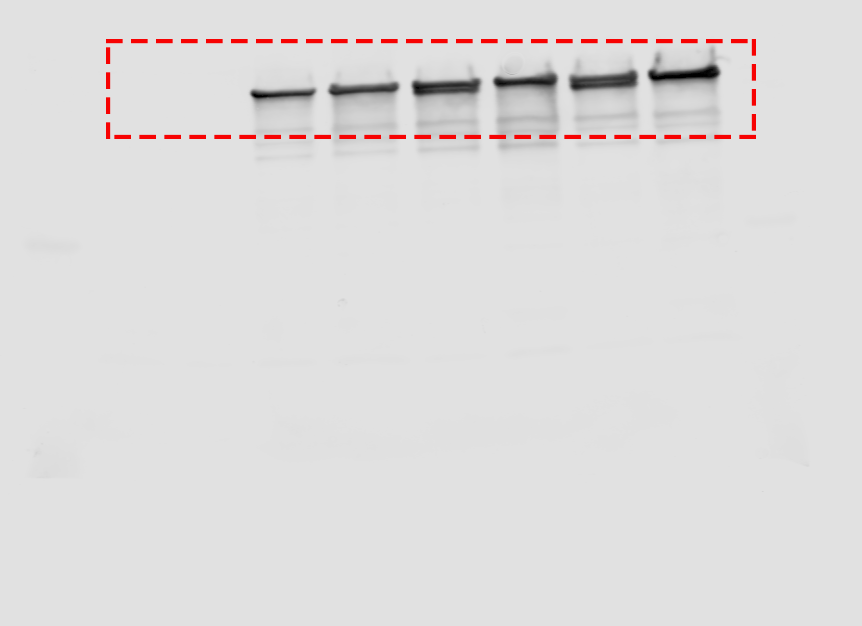

Supplement: Supplementary file 3 — Source data Fig. 1 [file 44319_2025_388_MOESM3_ESM.zip › Figure 1/1B_NP_rb800.tif]

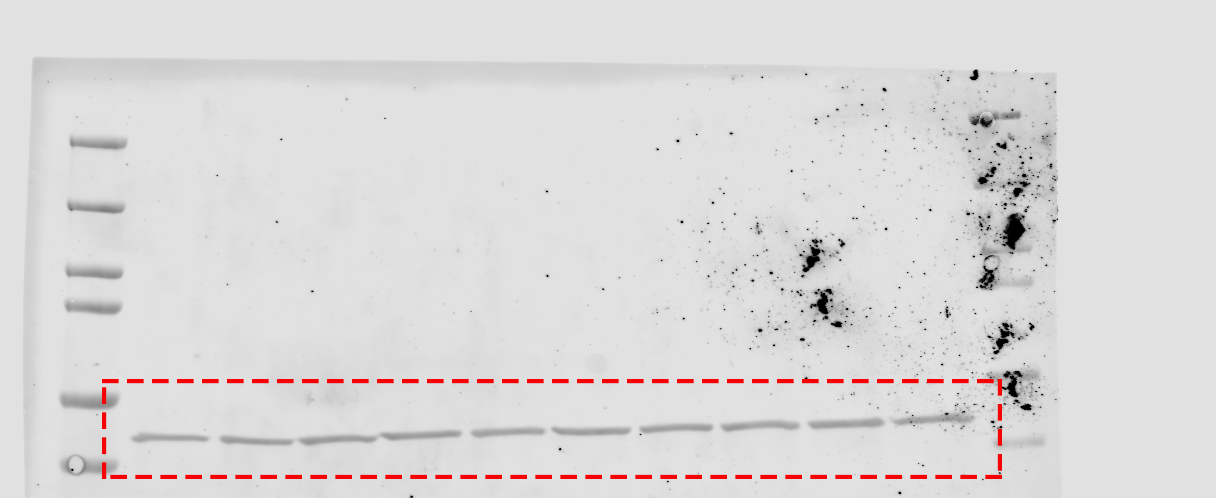

Supplement: Supplementary file 3 — Source data Fig. 1 [file 44319_2025_388_MOESM3_ESM.zip › Figure 1/1C_actin_ms700.tif]

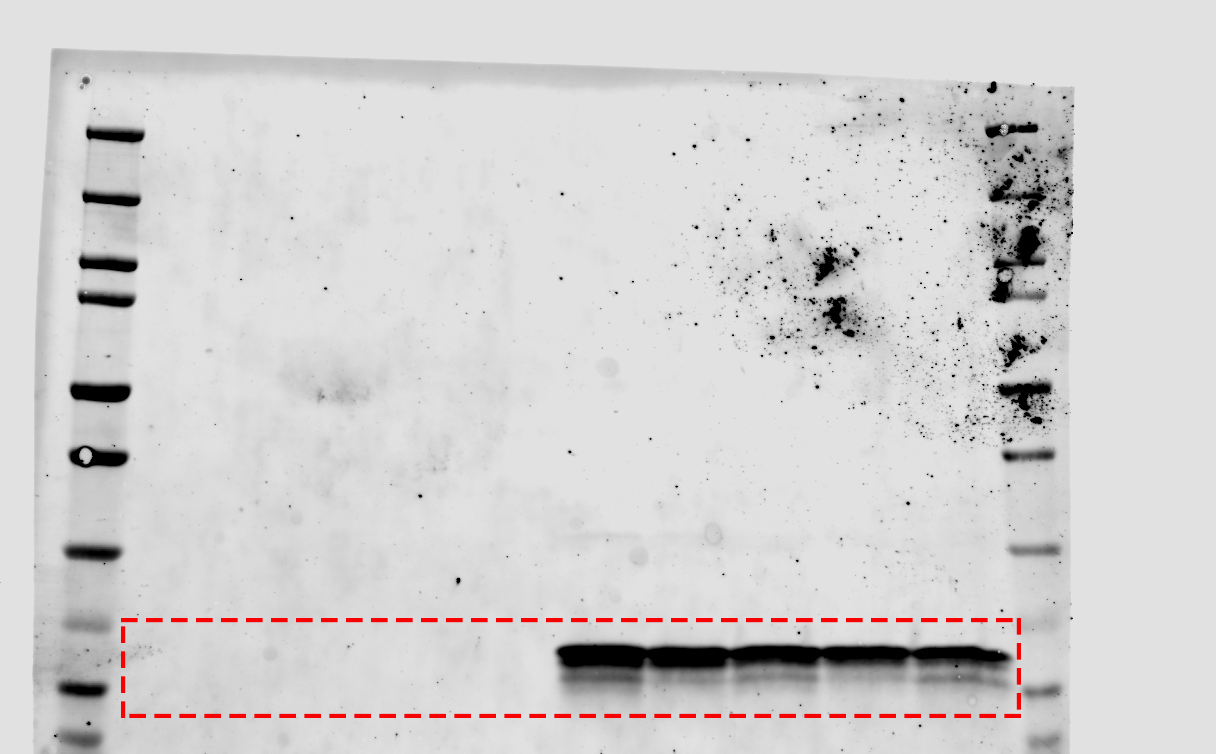

Supplement: Supplementary file 3 — Source data Fig. 1 [file 44319_2025_388_MOESM3_ESM.zip › Figure 1/1C_M2_ms700.tif]

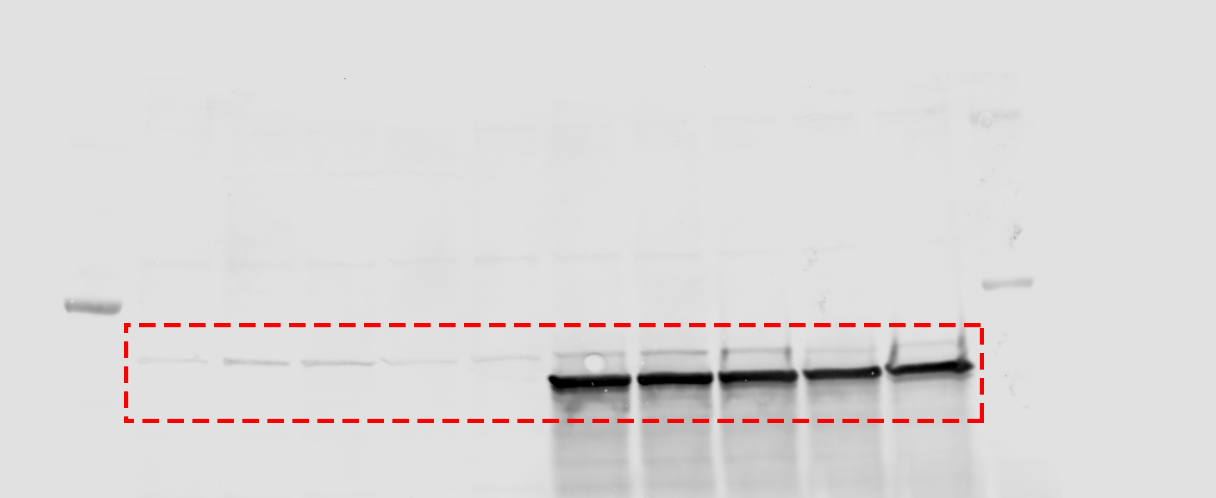

Supplement: Supplementary file 3 — Source data Fig. 1 [file 44319_2025_388_MOESM3_ESM.zip › Figure 1/1C_NP_rb800.tif]

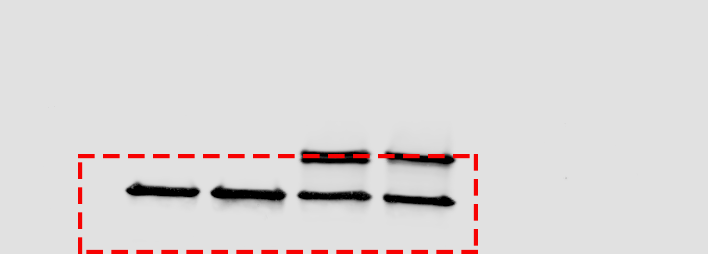

Supplement: Supplementary file 3 — Source data Fig. 1 [file 44319_2025_388_MOESM3_ESM.zip › Figure 1/1D_Actin_rb800.tif]

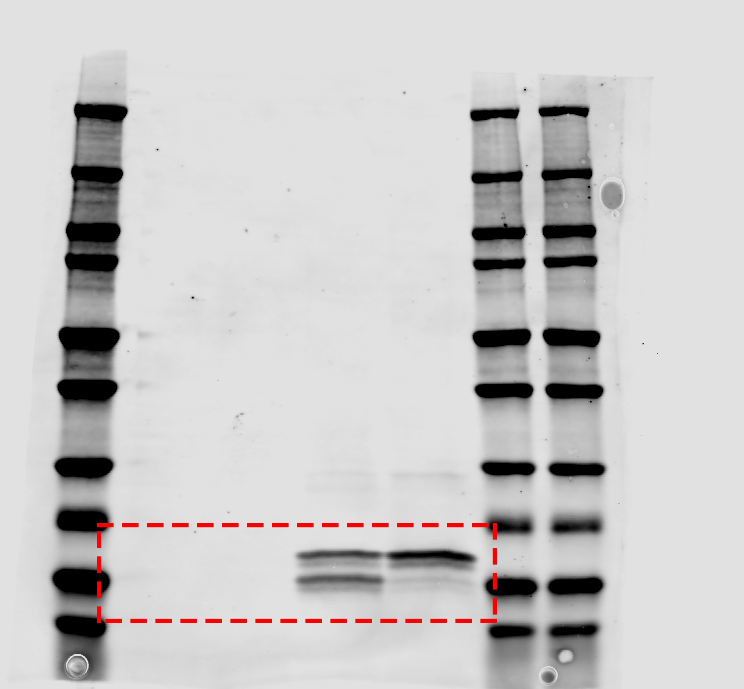

Supplement: Supplementary file 3 — Source data Fig. 1 [file 44319_2025_388_MOESM3_ESM.zip › Figure 1/1D_M2_ms700.tif]

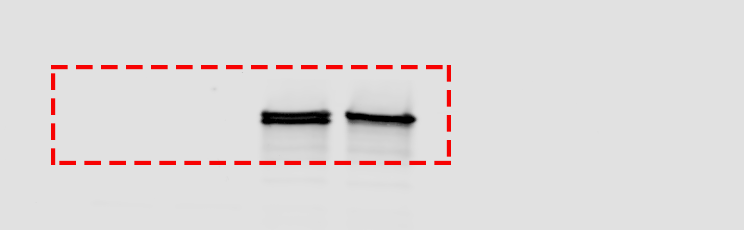

Supplement: Supplementary file 3 — Source data Fig. 1 [file 44319_2025_388_MOESM3_ESM.zip › Figure 1/1D_NP_rb800.tif]

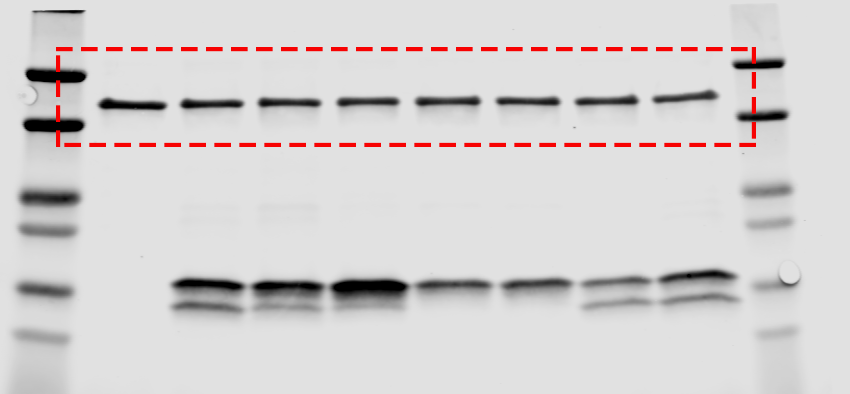

Supplement: Supplementary file 3 — Source data Fig. 1 [file 44319_2025_388_MOESM3_ESM.zip › Figure 1/1F_actin_ms700.tif]

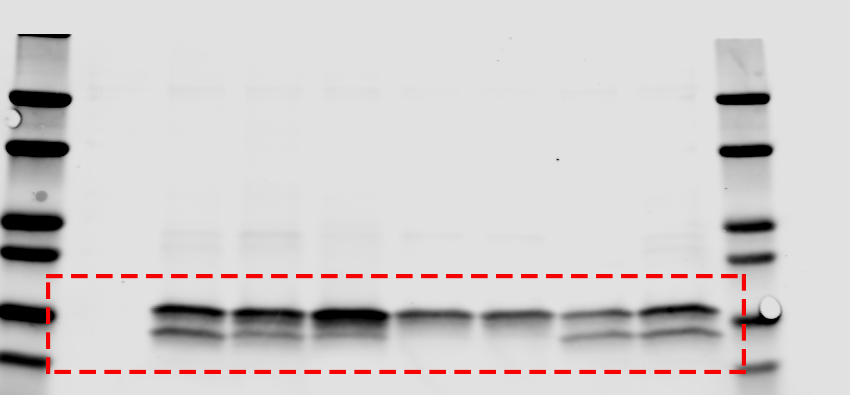

Supplement: Supplementary file 3 — Source data Fig. 1 [file 44319_2025_388_MOESM3_ESM.zip › Figure 1/1F_M2_ms700.tif]

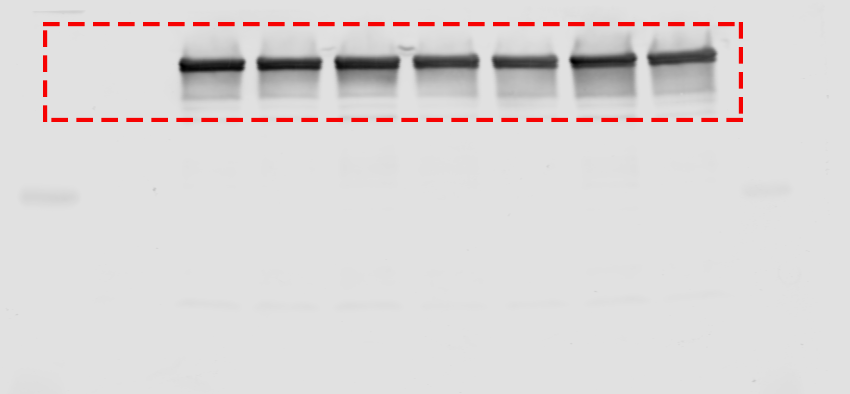

Supplement: Supplementary file 3 — Source data Fig. 1 [file 44319_2025_388_MOESM3_ESM.zip › Figure 1/1F_NP_rb800.tif]

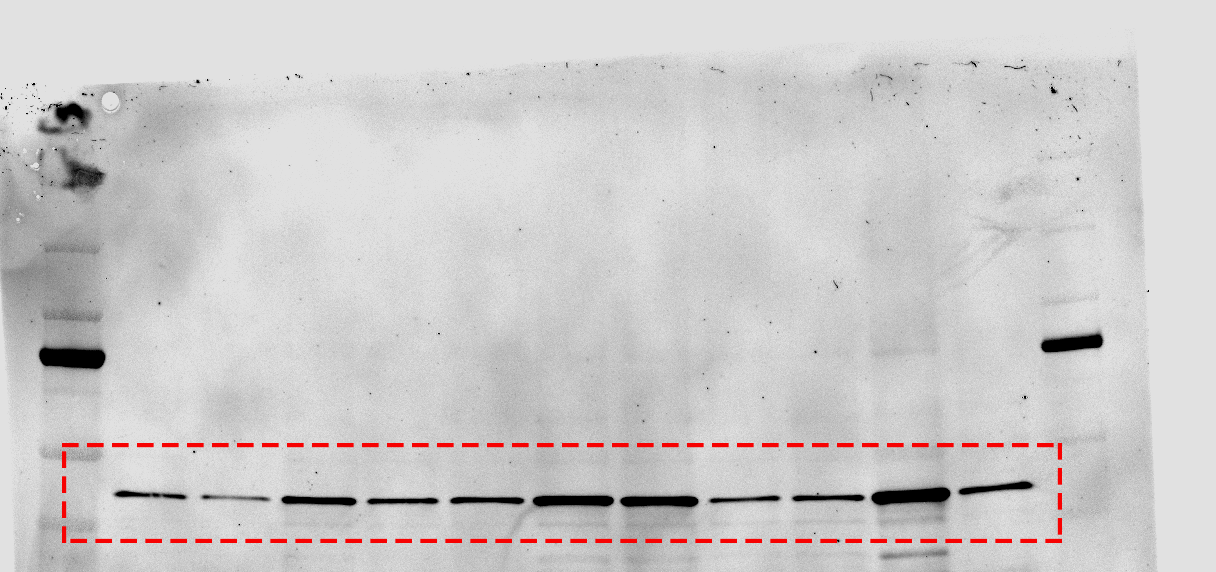

Supplement: Supplementary file 3 — Source data Fig. 1 [file 44319_2025_388_MOESM3_ESM.zip › Figure 1/1G_gel1_Actin_ms800.tif]

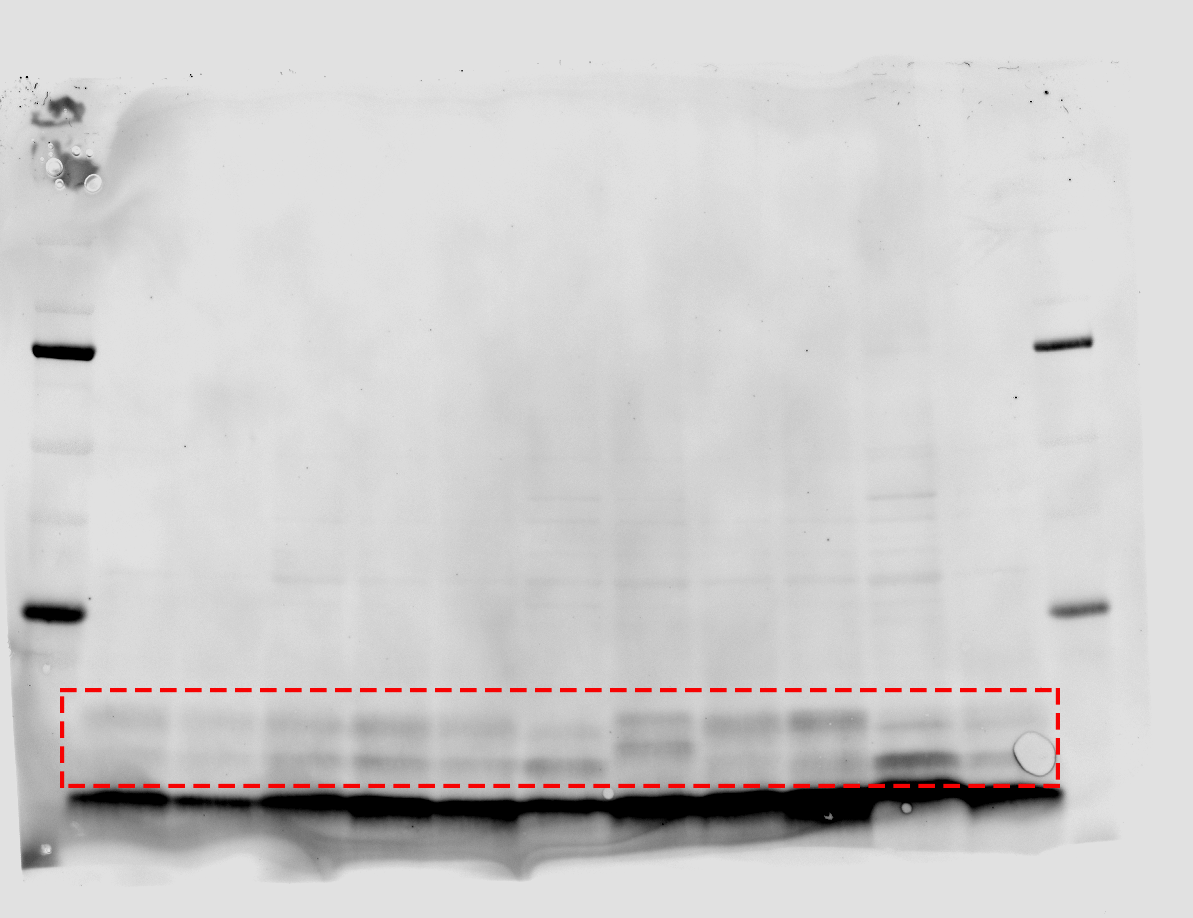

Supplement: Supplementary file 3 — Source data Fig. 1 [file 44319_2025_388_MOESM3_ESM.zip › Figure 1/1G_gel1_M2_ms800.tif]

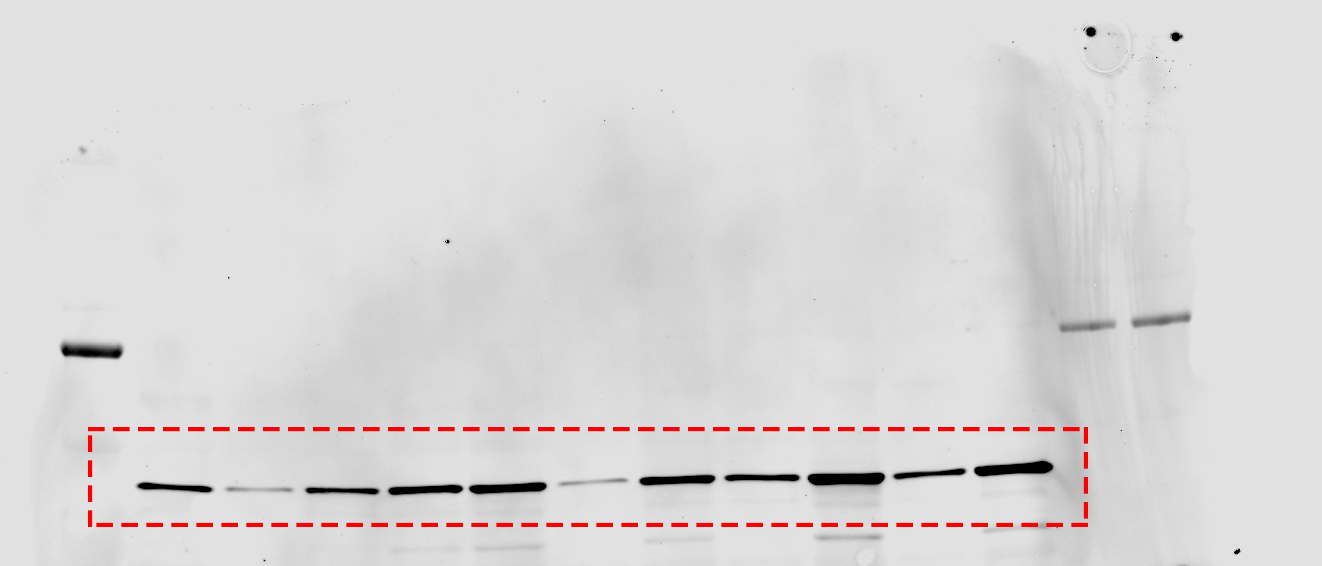

Supplement: Supplementary file 3 — Source data Fig. 1 [file 44319_2025_388_MOESM3_ESM.zip › Figure 1/1G_gel2_Actin_ms800.tif]

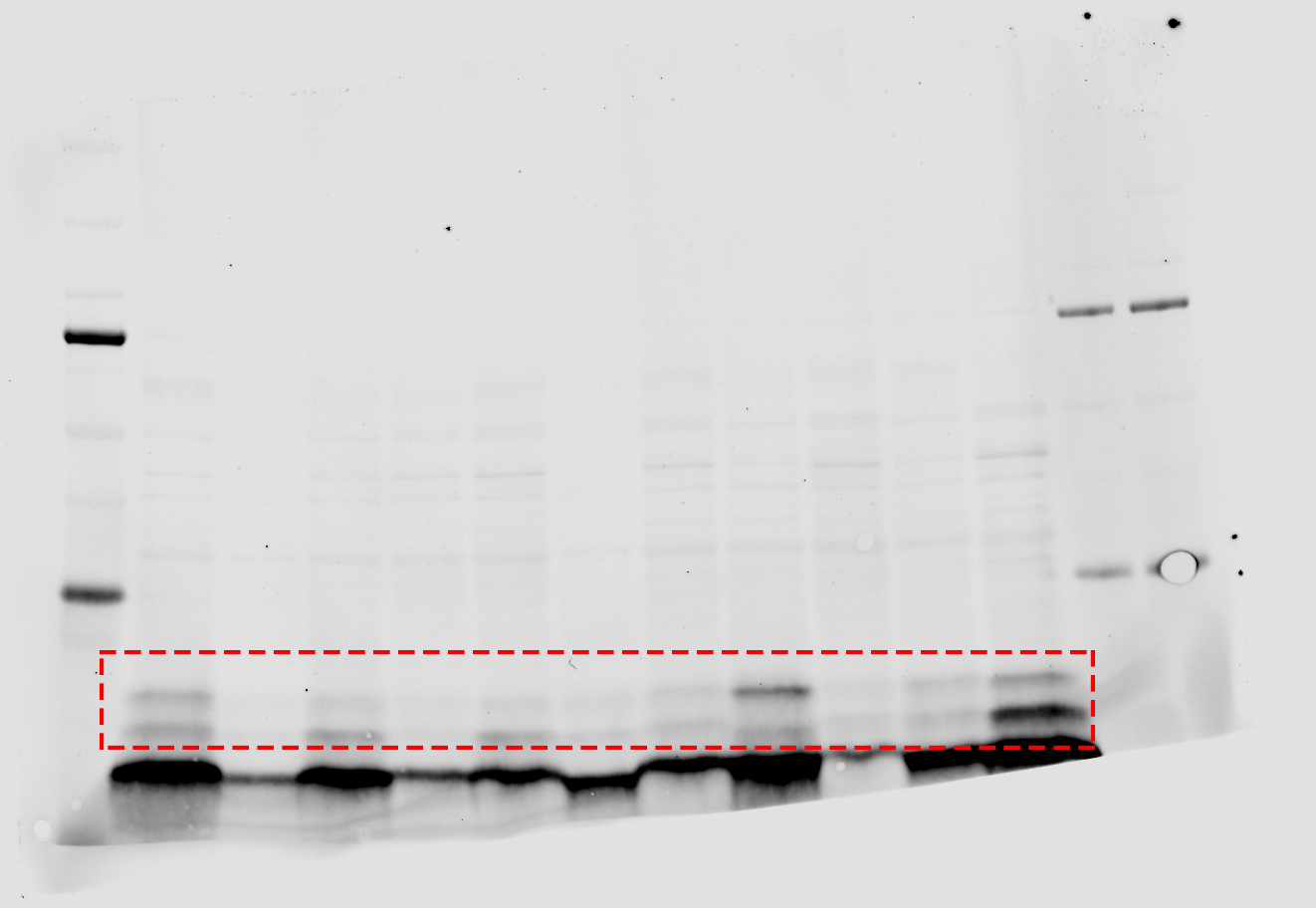

Supplement: Supplementary file 3 — Source data Fig. 1 [file 44319_2025_388_MOESM3_ESM.zip › Figure 1/1G_gel2_M2_ms800.tif]

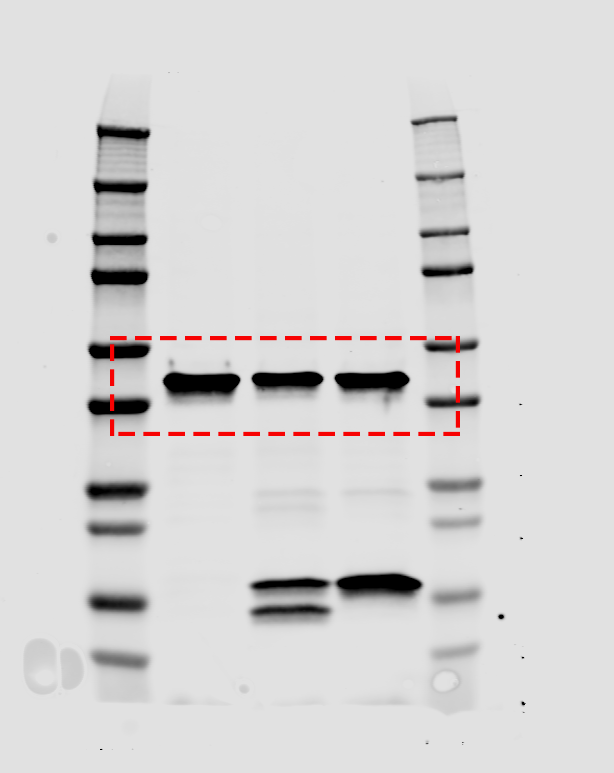

Supplement: Supplementary file 4 — Source data Fig. 2 [file 44319_2025_388_MOESM4_ESM.zip › Figure 2/2A_actin_ms700.tif]

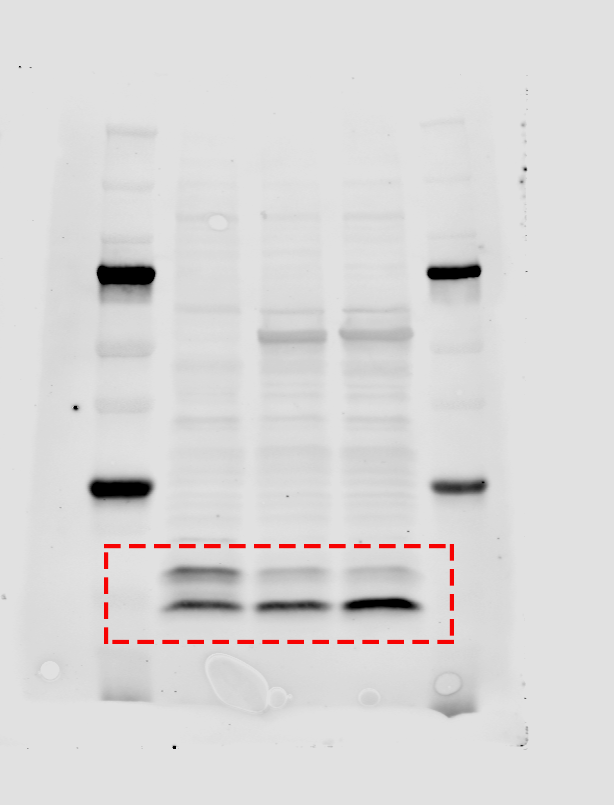

Supplement: Supplementary file 4 — Source data Fig. 2 [file 44319_2025_388_MOESM4_ESM.zip › Figure 2/2A_LC3_rb800.tif]

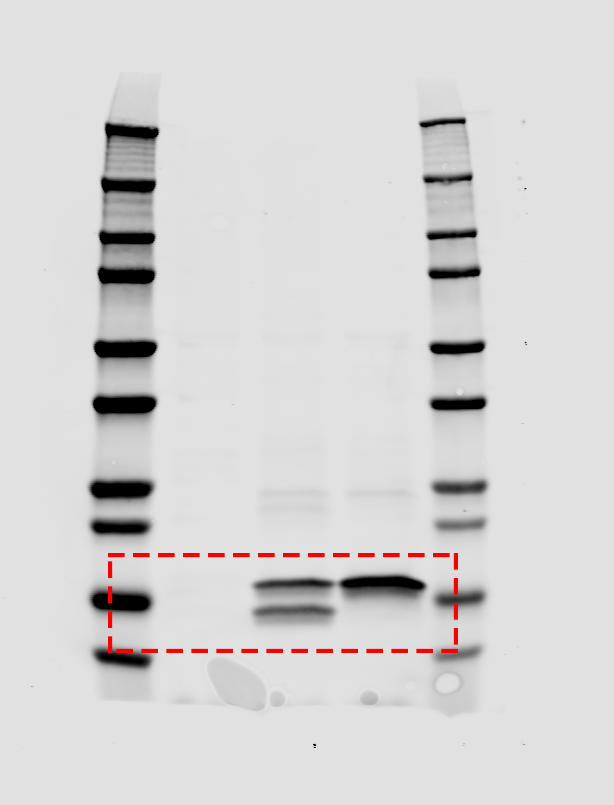

Supplement: Supplementary file 4 — Source data Fig. 2 [file 44319_2025_388_MOESM4_ESM.zip › Figure 2/2A_M2_ms700.tif]

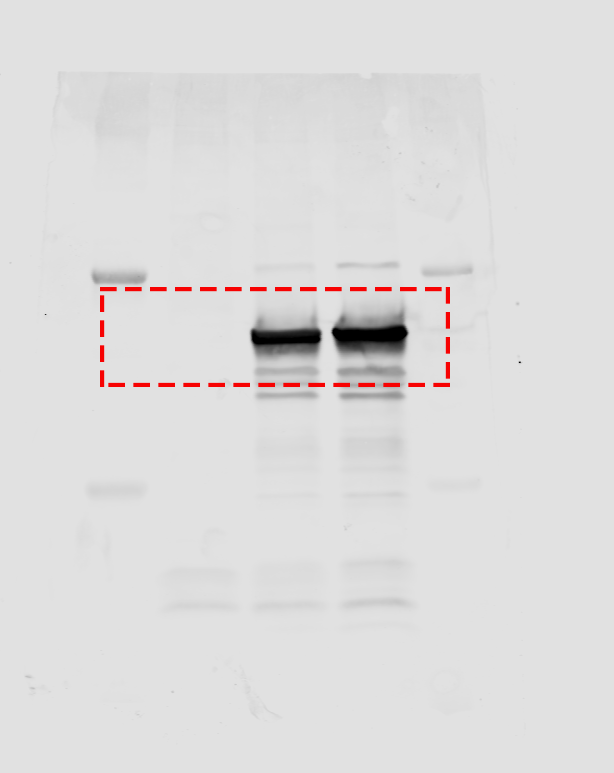

Supplement: Supplementary file 4 — Source data Fig. 2 [file 44319_2025_388_MOESM4_ESM.zip › Figure 2/2A_NP_rb800.tif]

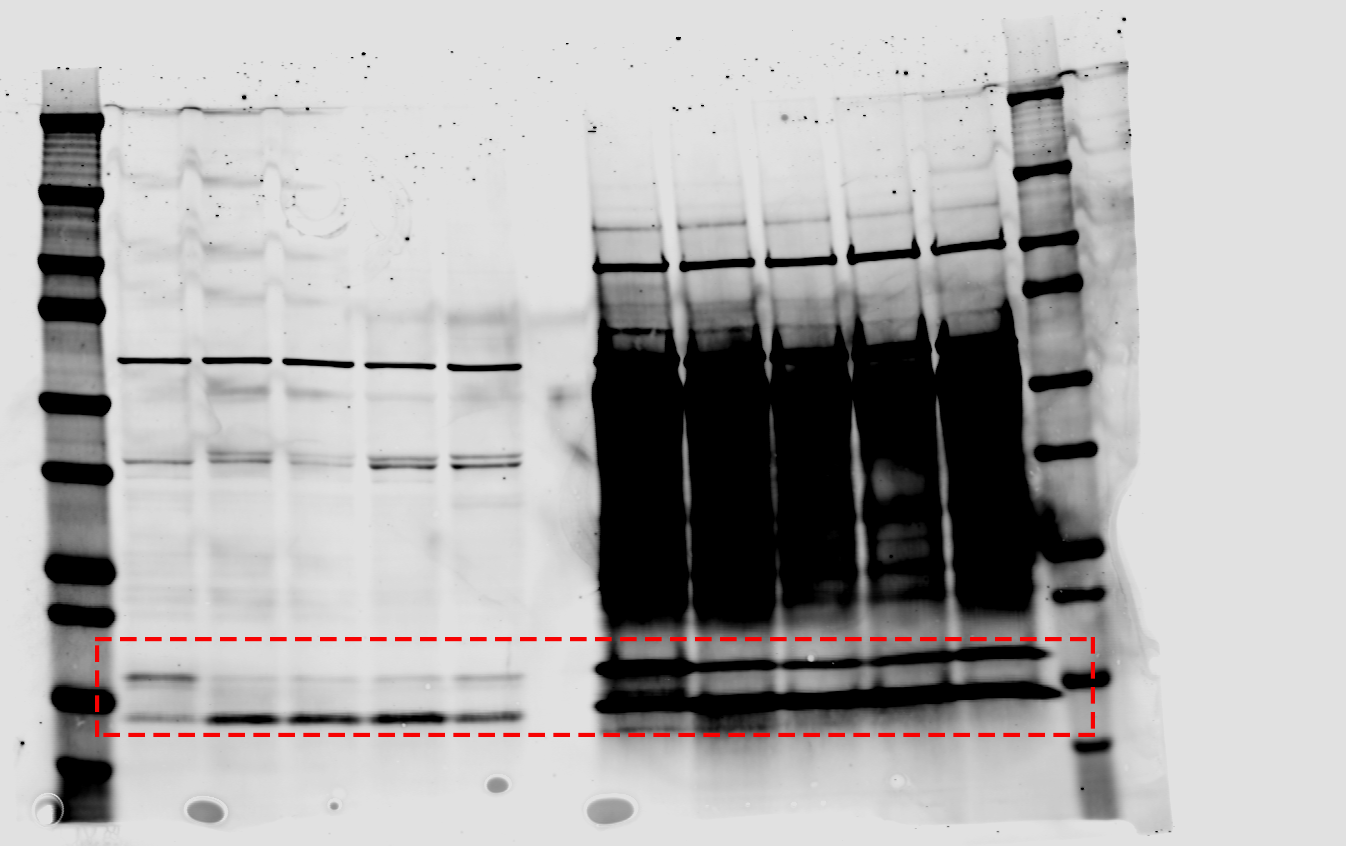

Supplement: Supplementary file 4 — Source data Fig. 2 [file 44319_2025_388_MOESM4_ESM.zip › Figure 2/2C_LC3B_rb700_A_highexp.tif]

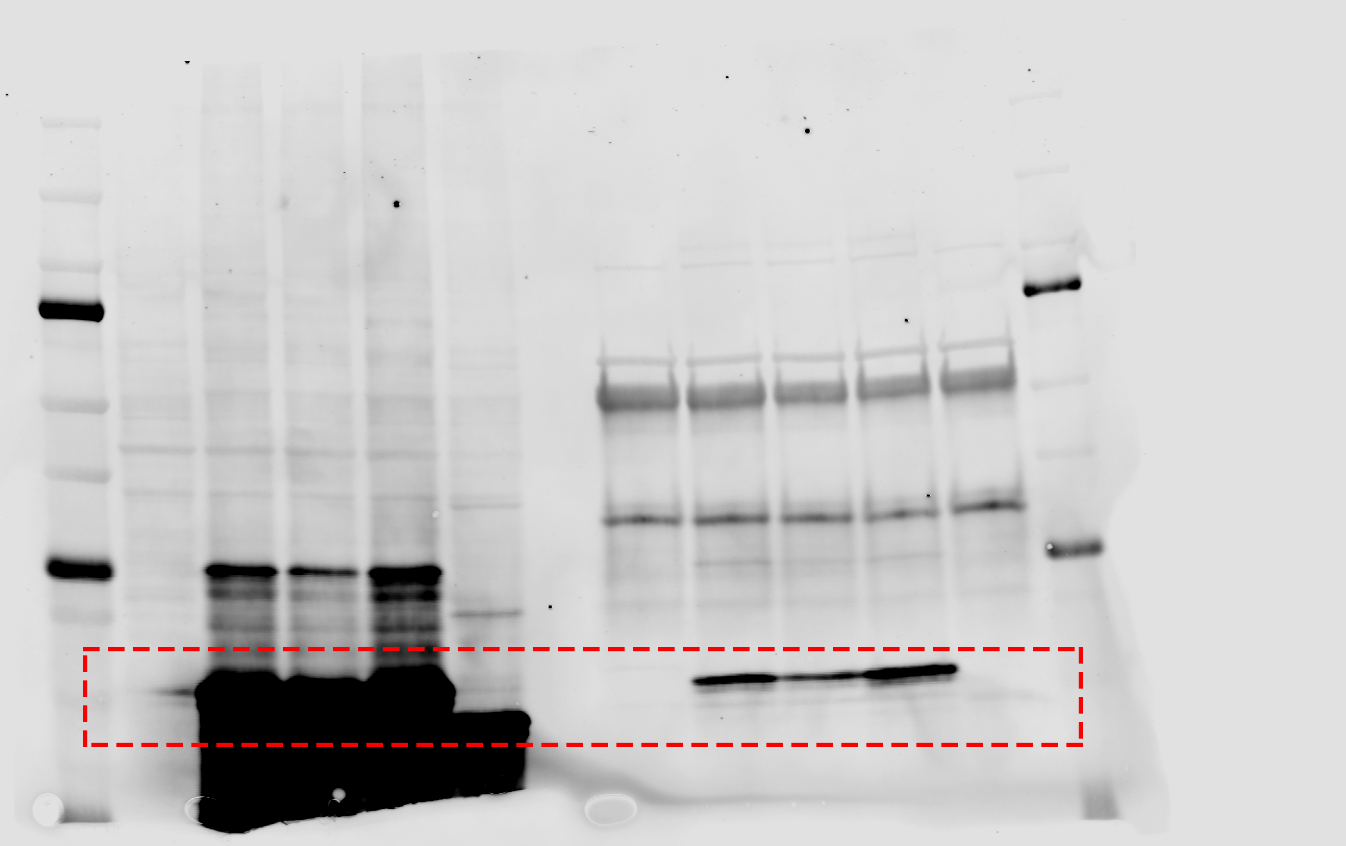

Supplement: Supplementary file 4 — Source data Fig. 2 [file 44319_2025_388_MOESM4_ESM.zip › Figure 2/2C_M2_ms800_A_highexp.tif]

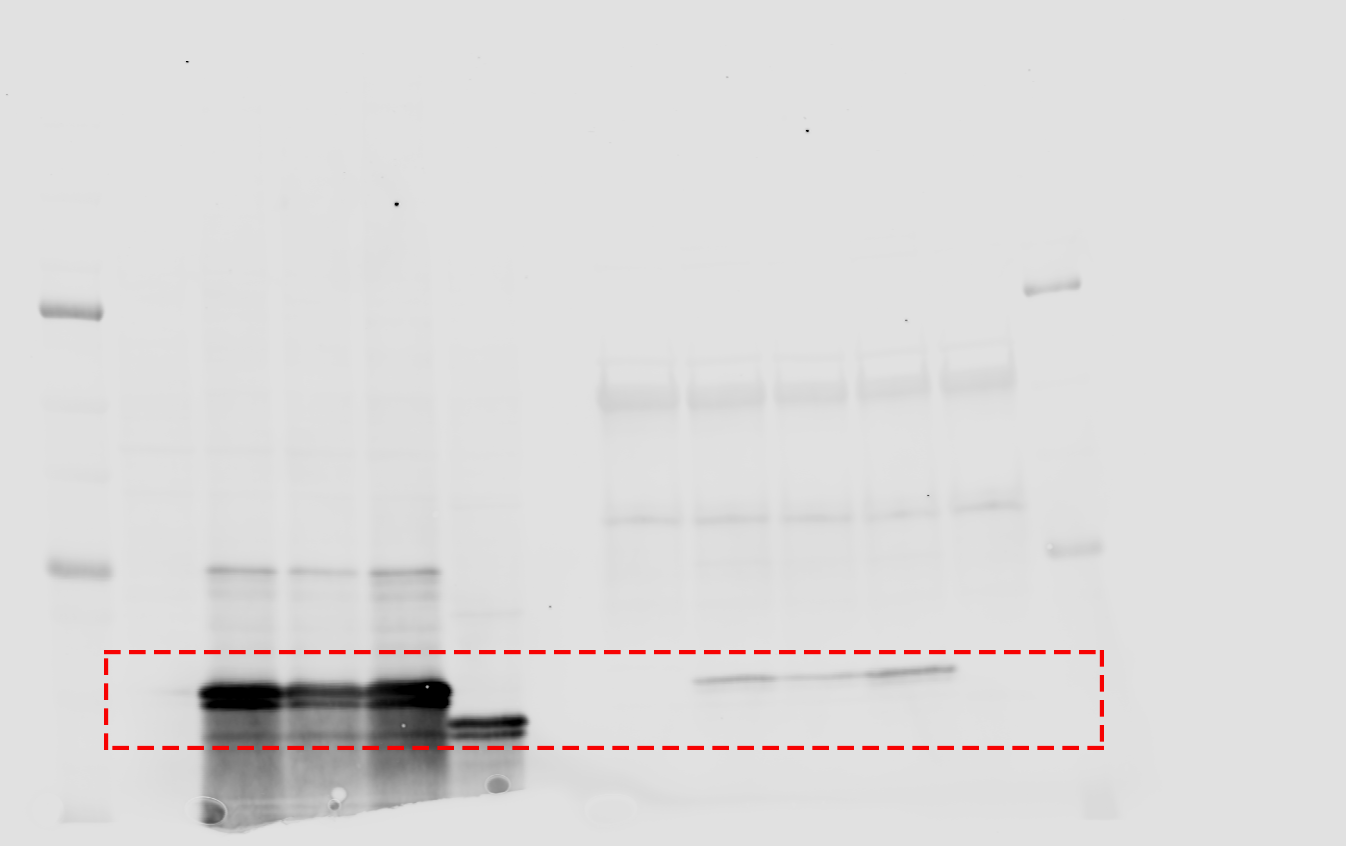

Supplement: Supplementary file 4 — Source data Fig. 2 [file 44319_2025_388_MOESM4_ESM.zip › Figure 2/2C_M2_ms800_A_lowexp.tif]

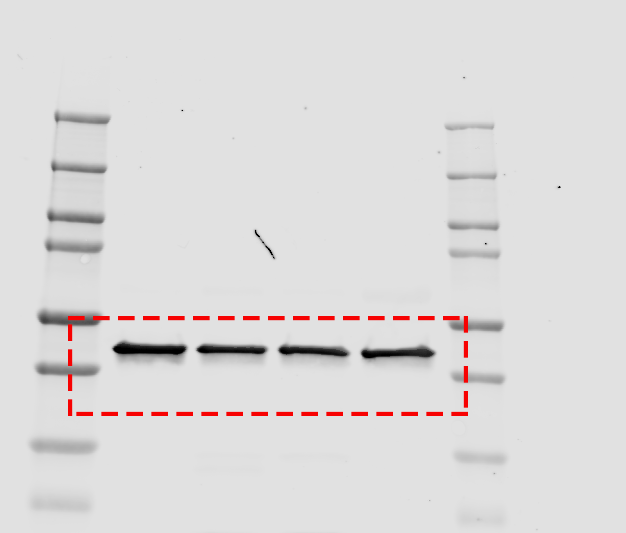

Supplement: Supplementary file 4 — Source data Fig. 2 [file 44319_2025_388_MOESM4_ESM.zip › Figure 2/2D_actin_ms700.tif]

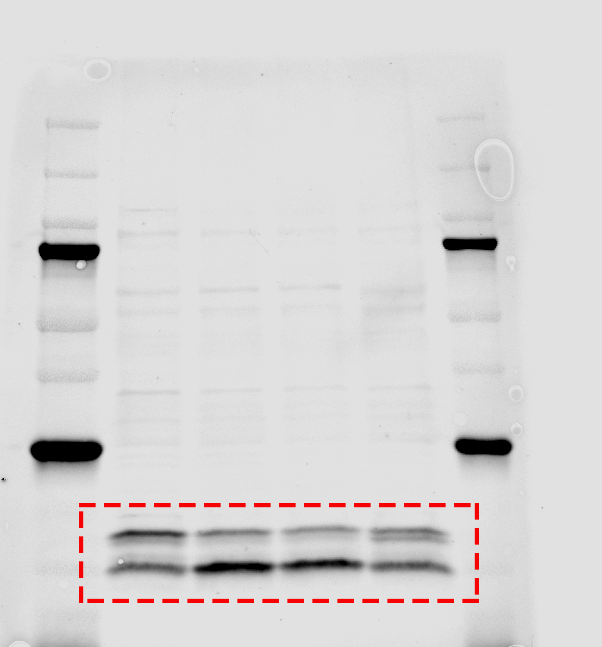

Supplement: Supplementary file 4 — Source data Fig. 2 [file 44319_2025_388_MOESM4_ESM.zip › Figure 2/2D_LC3B_rb800.tif]

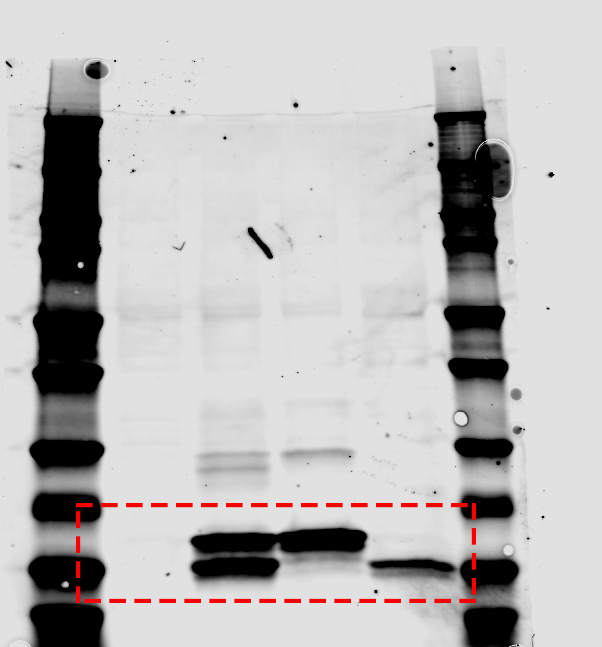

Supplement: Supplementary file 4 — Source data Fig. 2 [file 44319_2025_388_MOESM4_ESM.zip › Figure 2/2D_M2_ms700_A_highexp.tif]

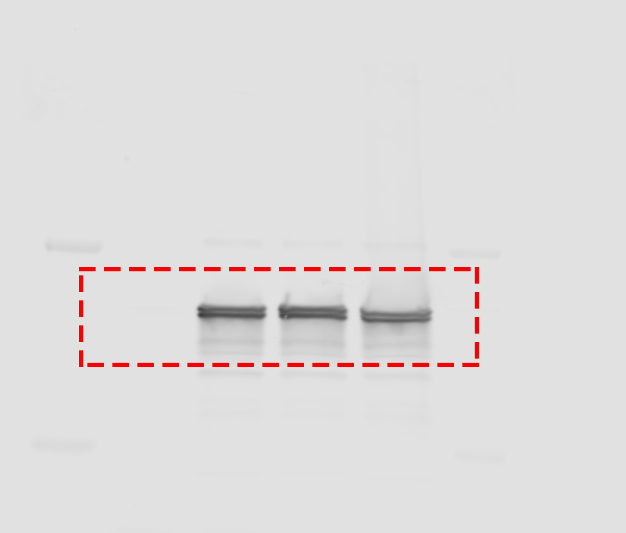

Supplement: Supplementary file 4 — Source data Fig. 2 [file 44319_2025_388_MOESM4_ESM.zip › Figure 2/2D_NP_rb800.tif]

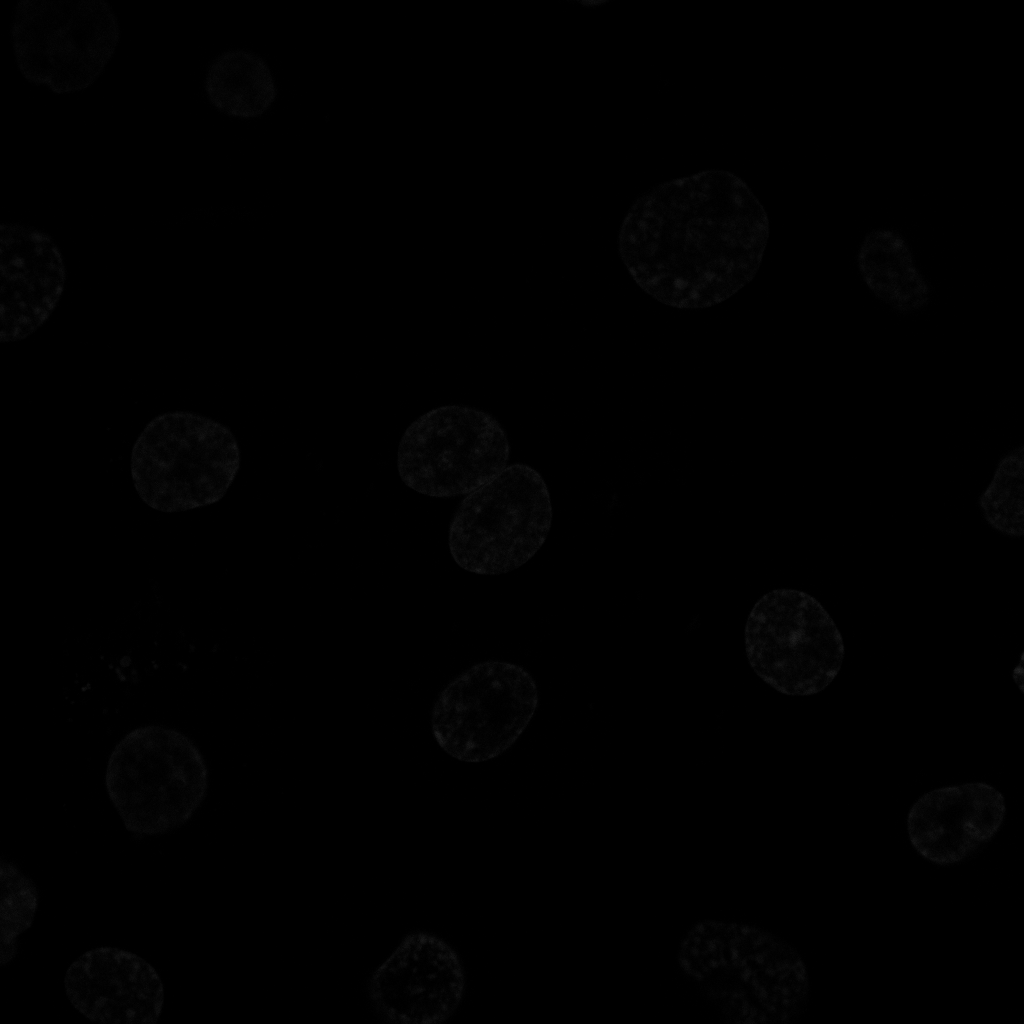

Supplement: Supplementary file 5 — Source data Fig. 3 [file 44319_2025_388_MOESM5_ESM.zip › Figure 3/3A_Hoechst_Perm_D85A.tif]

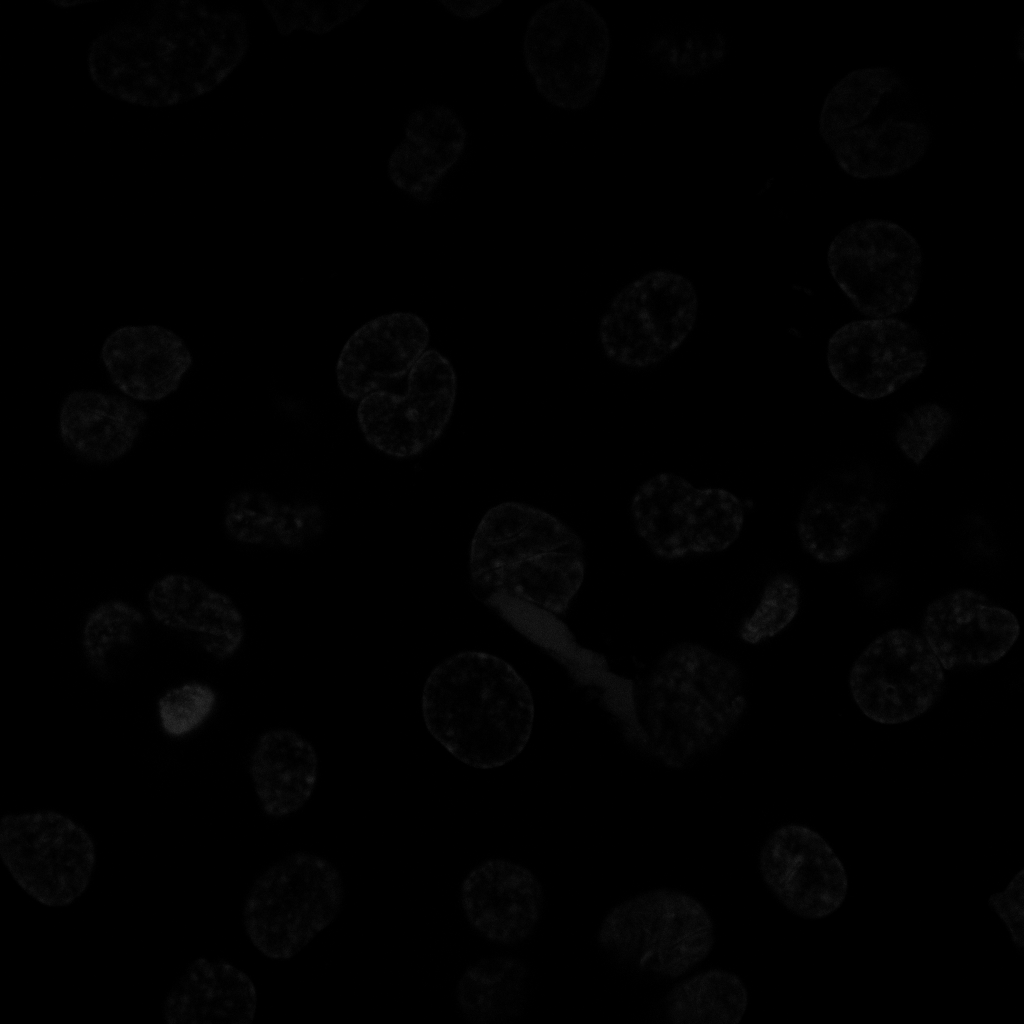

Supplement: Supplementary file 5 — Source data Fig. 3 [file 44319_2025_388_MOESM5_ESM.zip › Figure 3/3A_Hoechst_Perm_delta8697.tif]

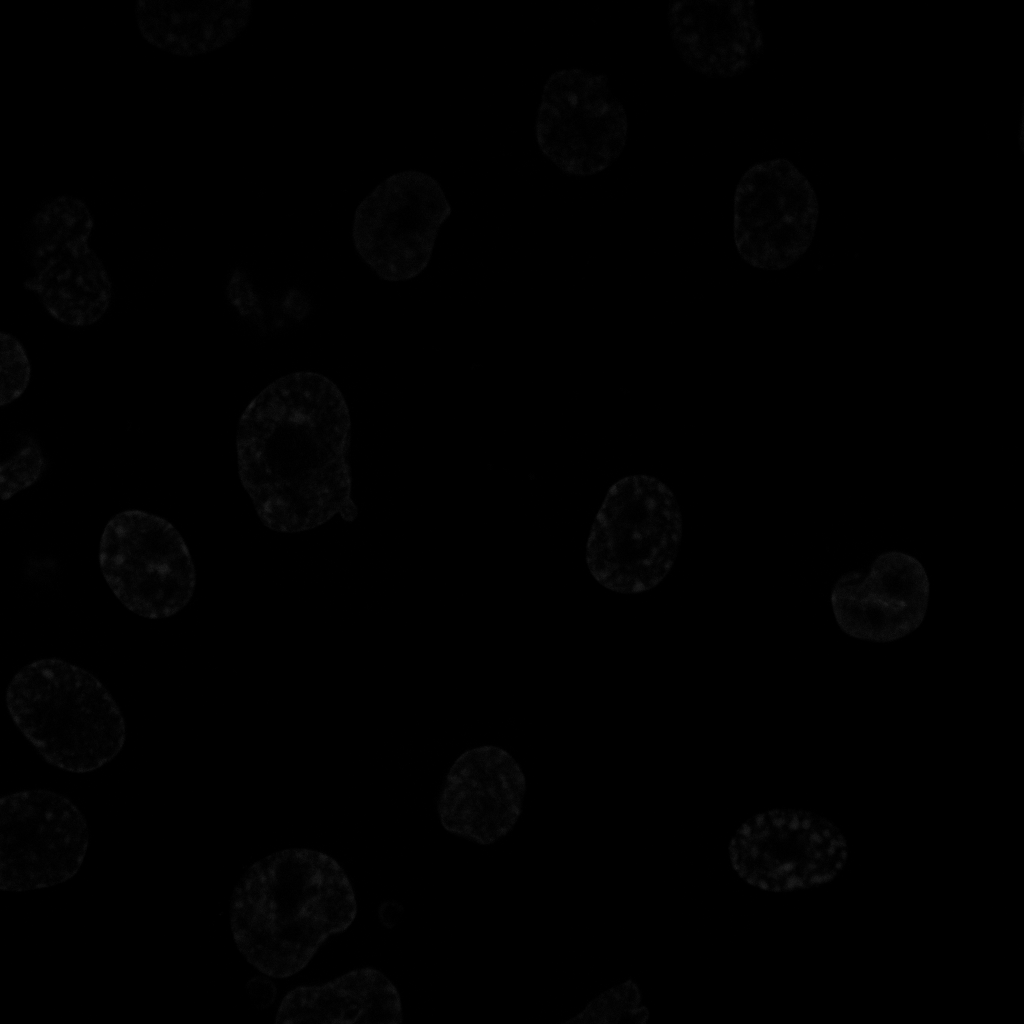

Supplement: Supplementary file 5 — Source data Fig. 3 [file 44319_2025_388_MOESM5_ESM.zip › Figure 3/3A_Hoechst_Perm_WT.tif]

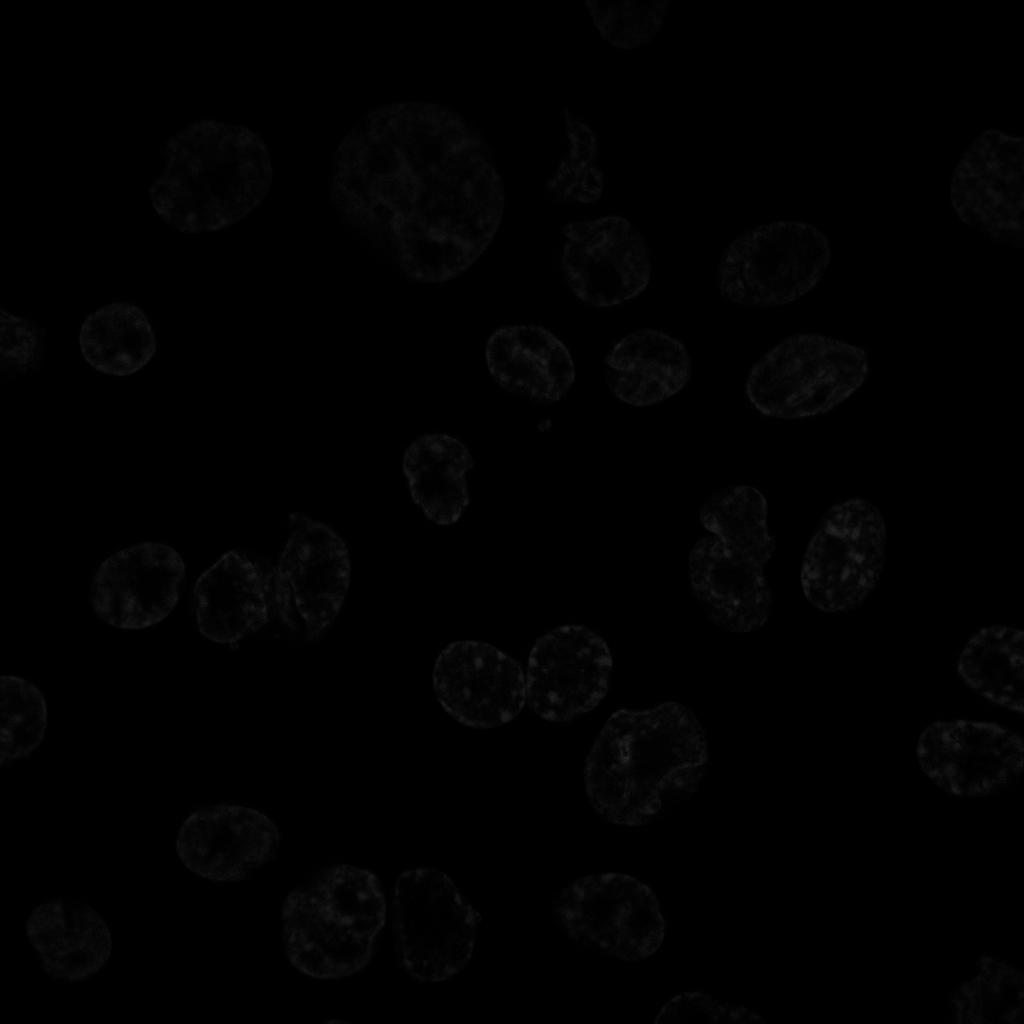

Supplement: Supplementary file 5 — Source data Fig. 3 [file 44319_2025_388_MOESM5_ESM.zip › Figure 3/3A_Hoechst_Surf_D85A.tif]

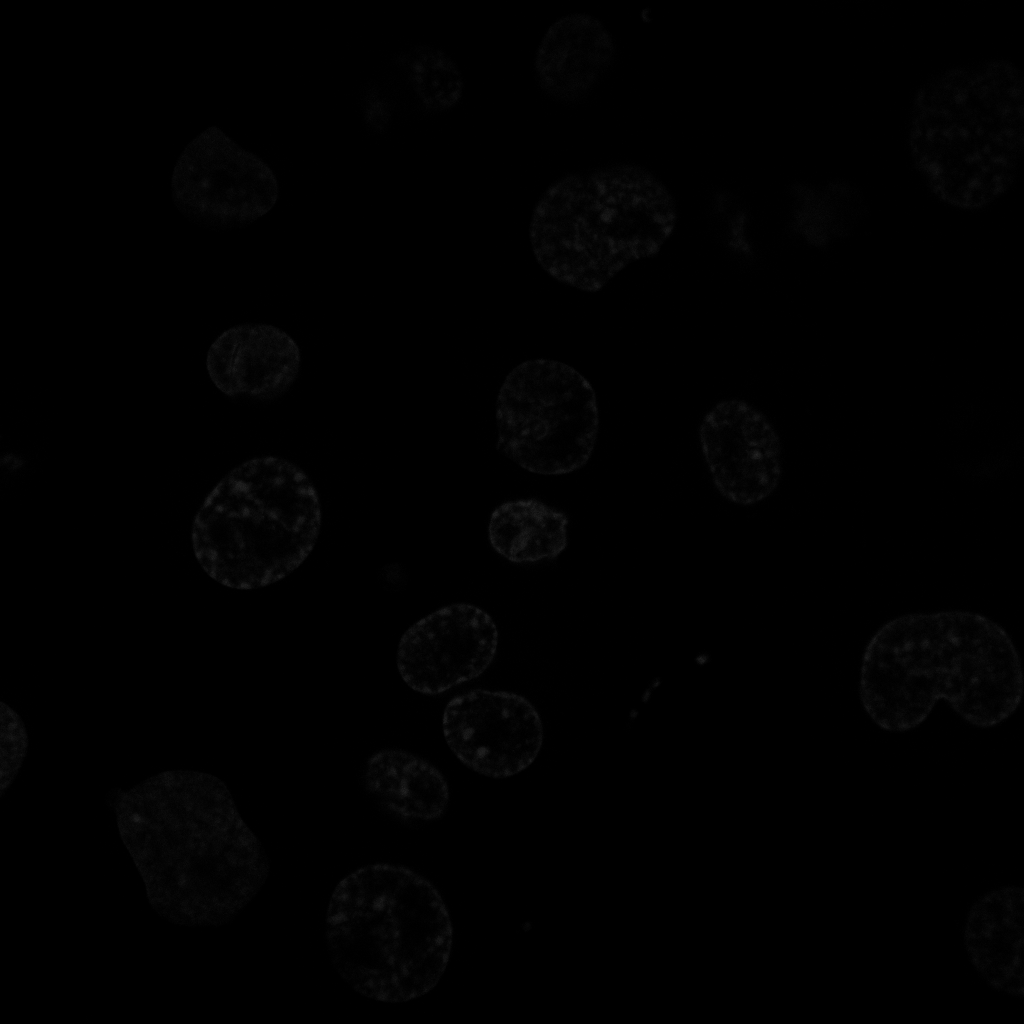

Supplement: Supplementary file 5 — Source data Fig. 3 [file 44319_2025_388_MOESM5_ESM.zip › Figure 3/3A_Hoechst_Surf_delta8697.tif]

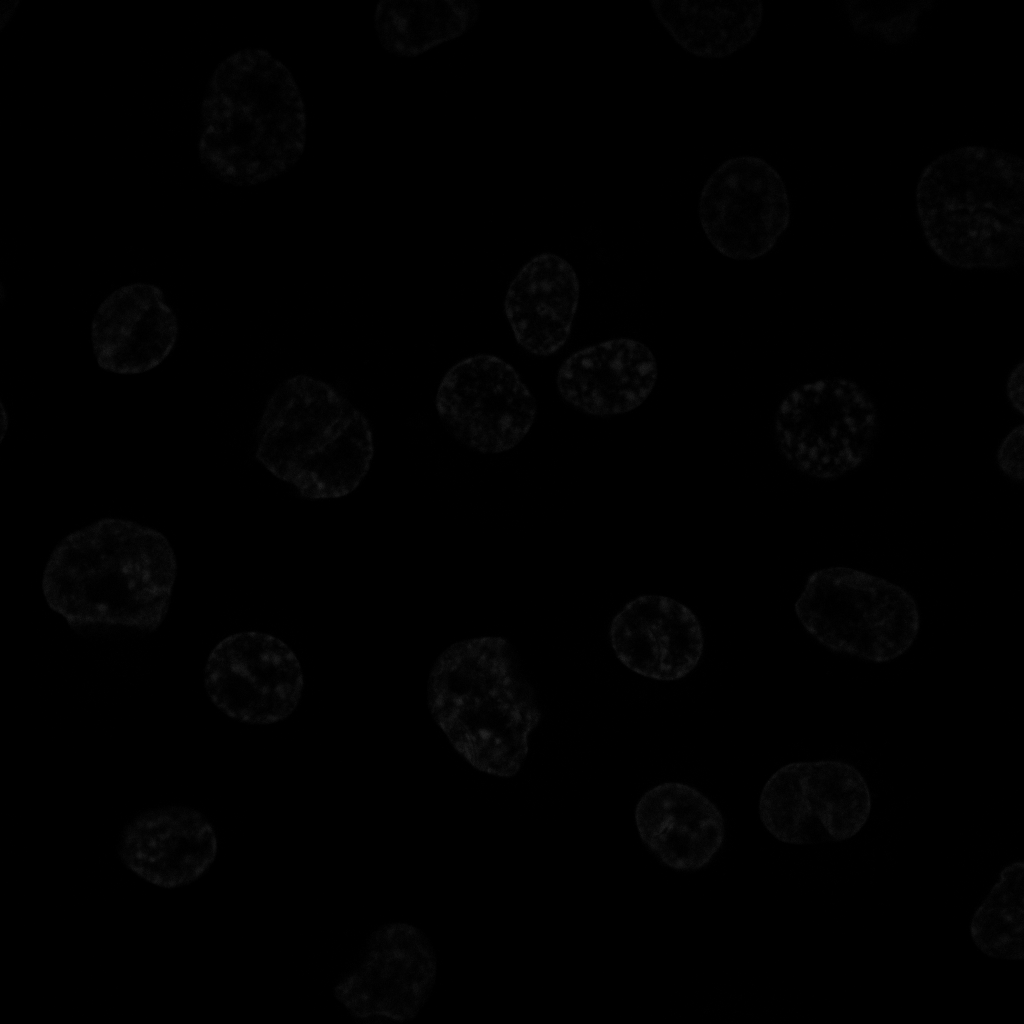

Supplement: Supplementary file 5 — Source data Fig. 3 [file 44319_2025_388_MOESM5_ESM.zip › Figure 3/3A_Hoechst_Surf_WT.tif]

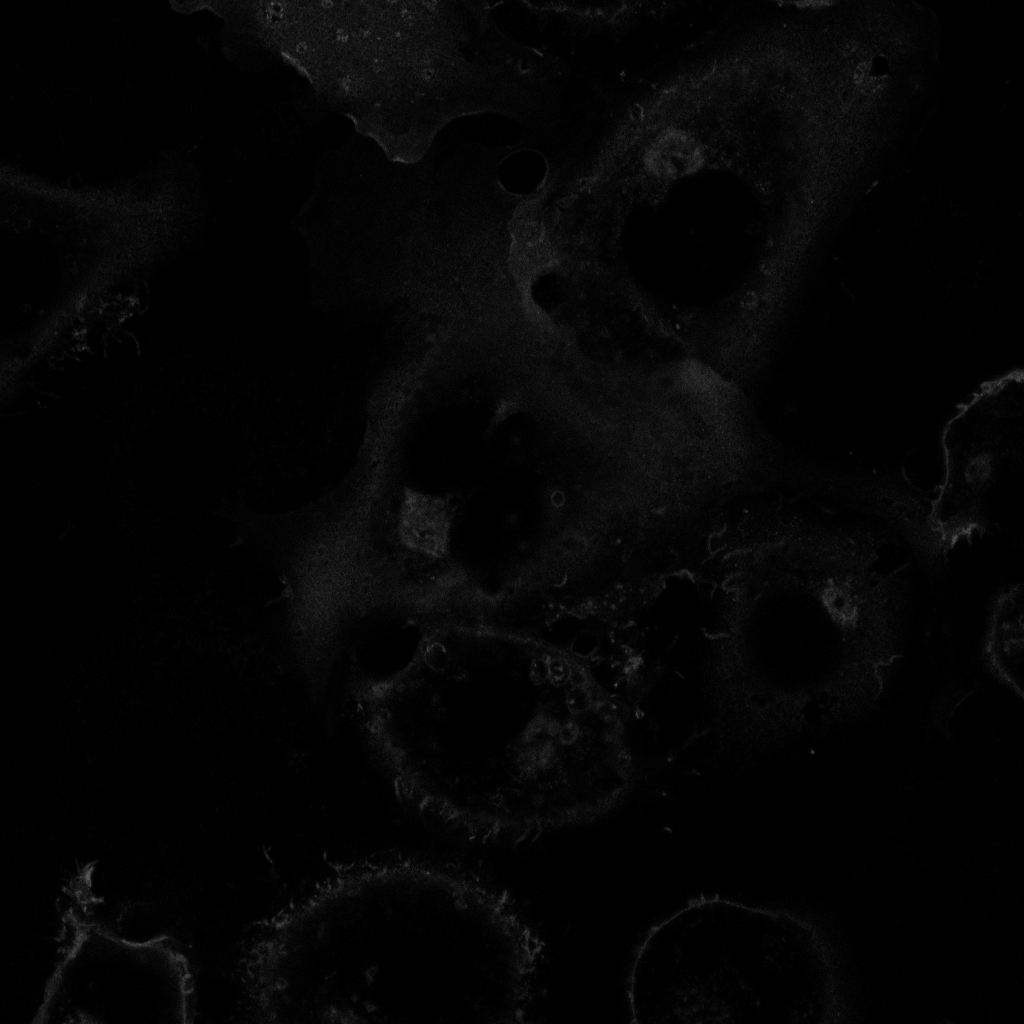

Supplement: Supplementary file 5 — Source data Fig. 3 [file 44319_2025_388_MOESM5_ESM.zip › Figure 3/3A_M2_Perm_D85A.tif]

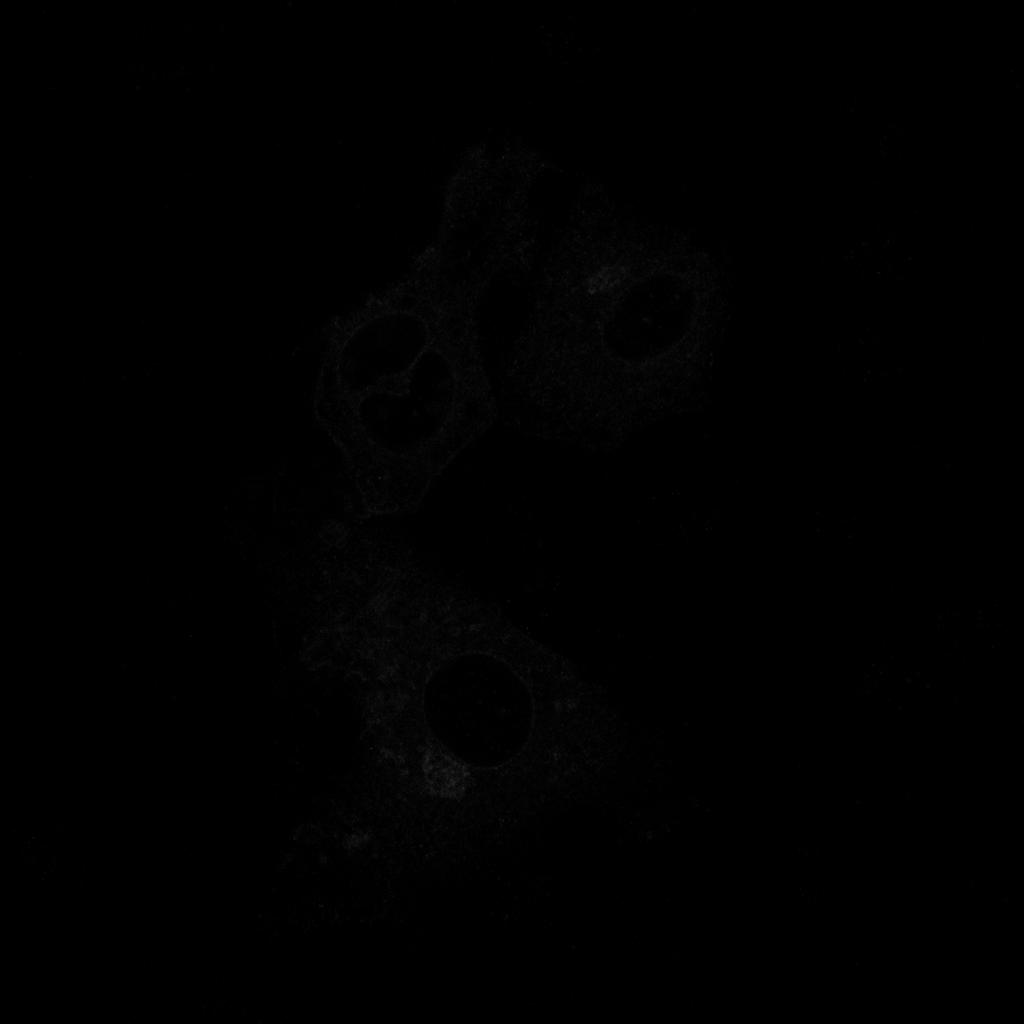

Supplement: Supplementary file 5 — Source data Fig. 3 [file 44319_2025_388_MOESM5_ESM.zip › Figure 3/3A_M2_Perm_delta8697.tif]

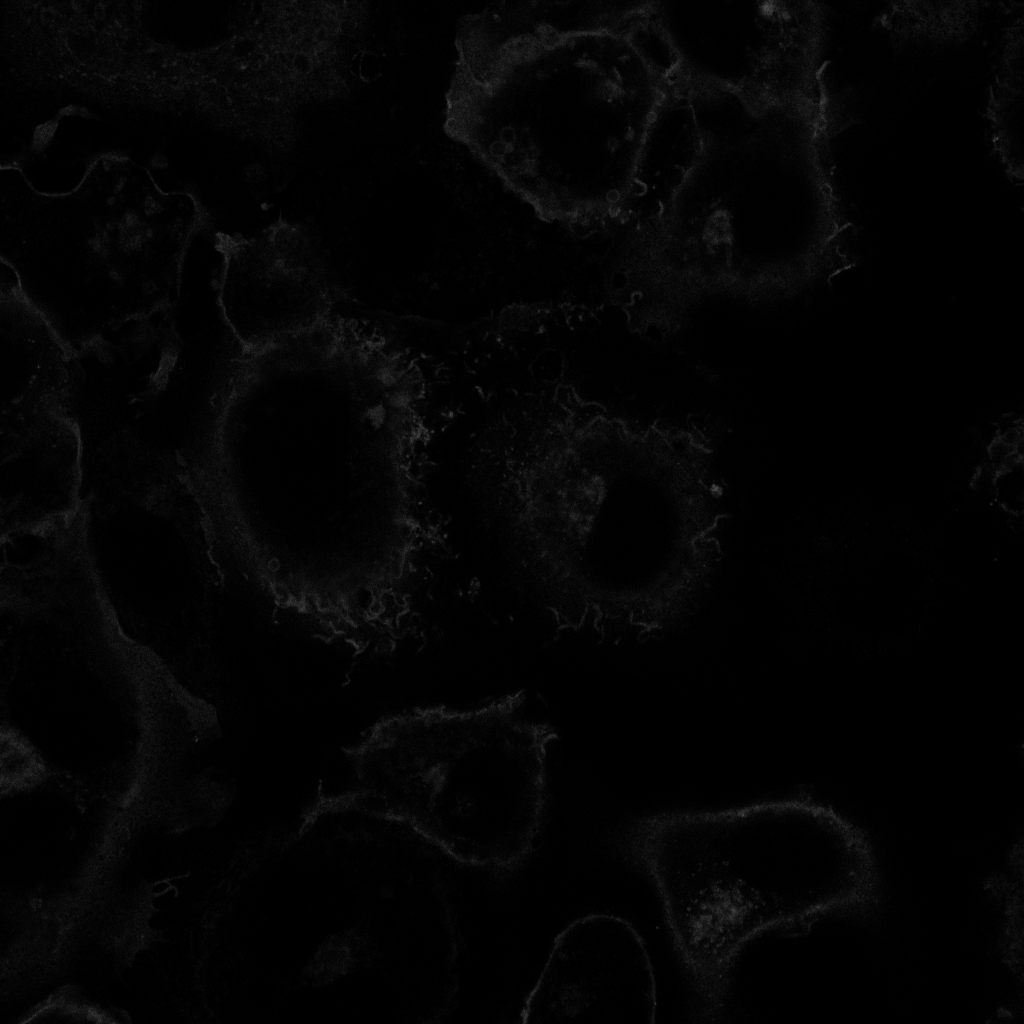

Supplement: Supplementary file 5 — Source data Fig. 3 [file 44319_2025_388_MOESM5_ESM.zip › Figure 3/3A_M2_Perm_WT.tif]

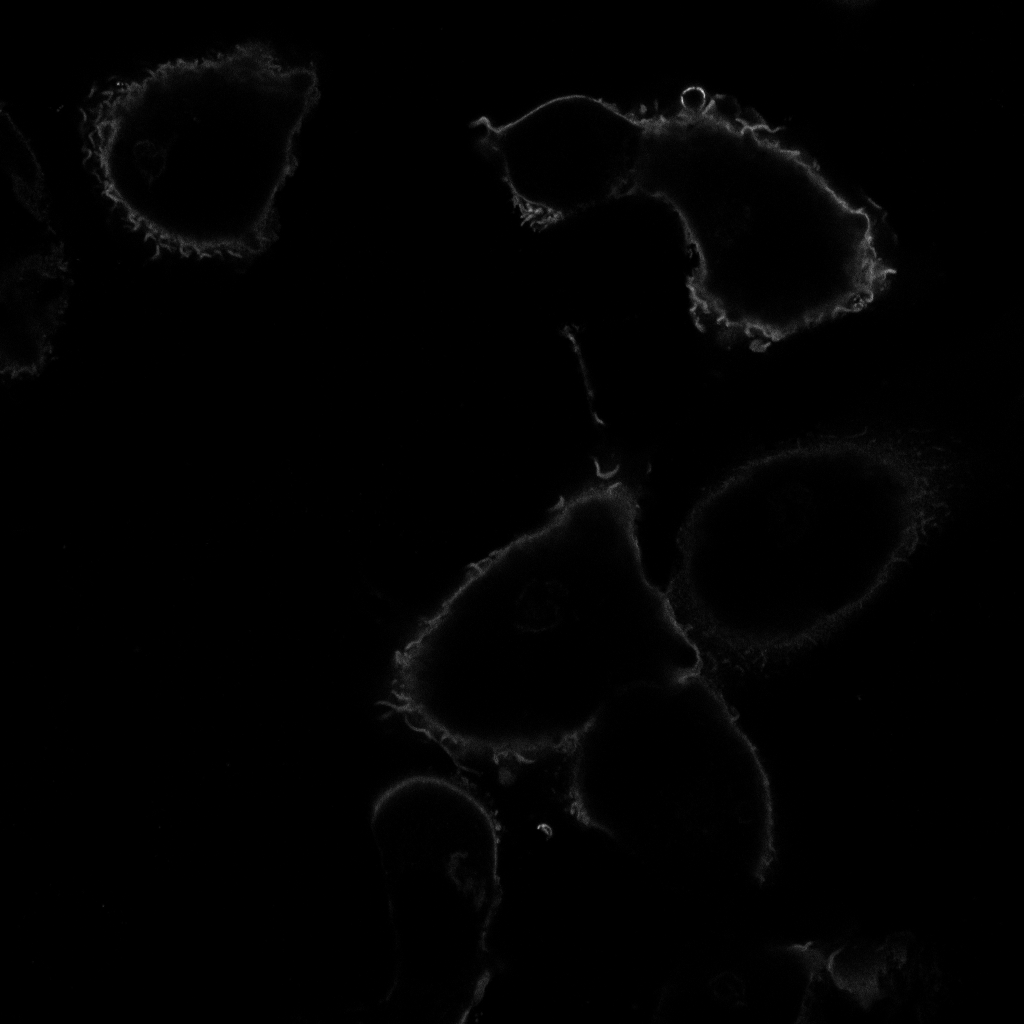

Supplement: Supplementary file 5 — Source data Fig. 3 [file 44319_2025_388_MOESM5_ESM.zip › Figure 3/3A_M2_Surf_D85A.tif]

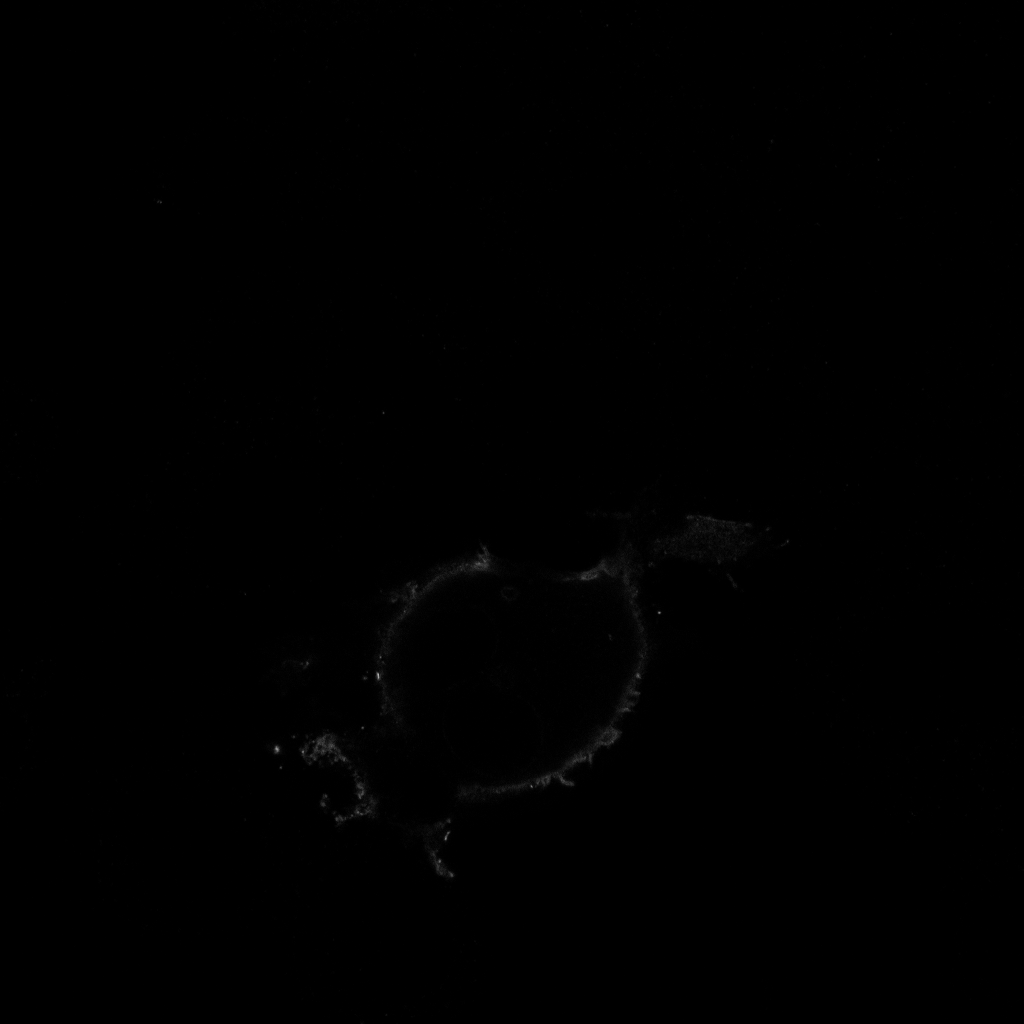

Supplement: Supplementary file 5 — Source data Fig. 3 [file 44319_2025_388_MOESM5_ESM.zip › Figure 3/3A_M2_Surf_delta8697.tif]

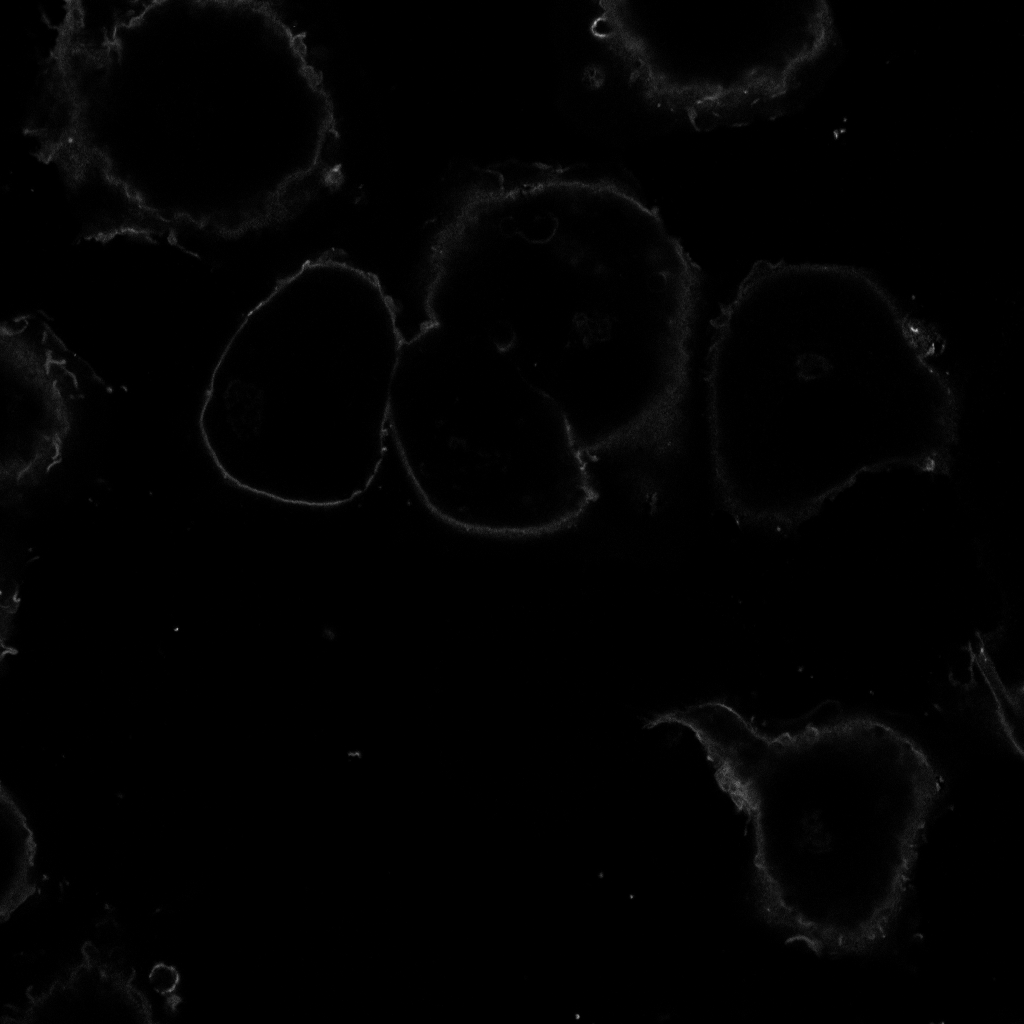

Supplement: Supplementary file 5 — Source data Fig. 3 [file 44319_2025_388_MOESM5_ESM.zip › Figure 3/3A_M2_Surf_WT.tif]

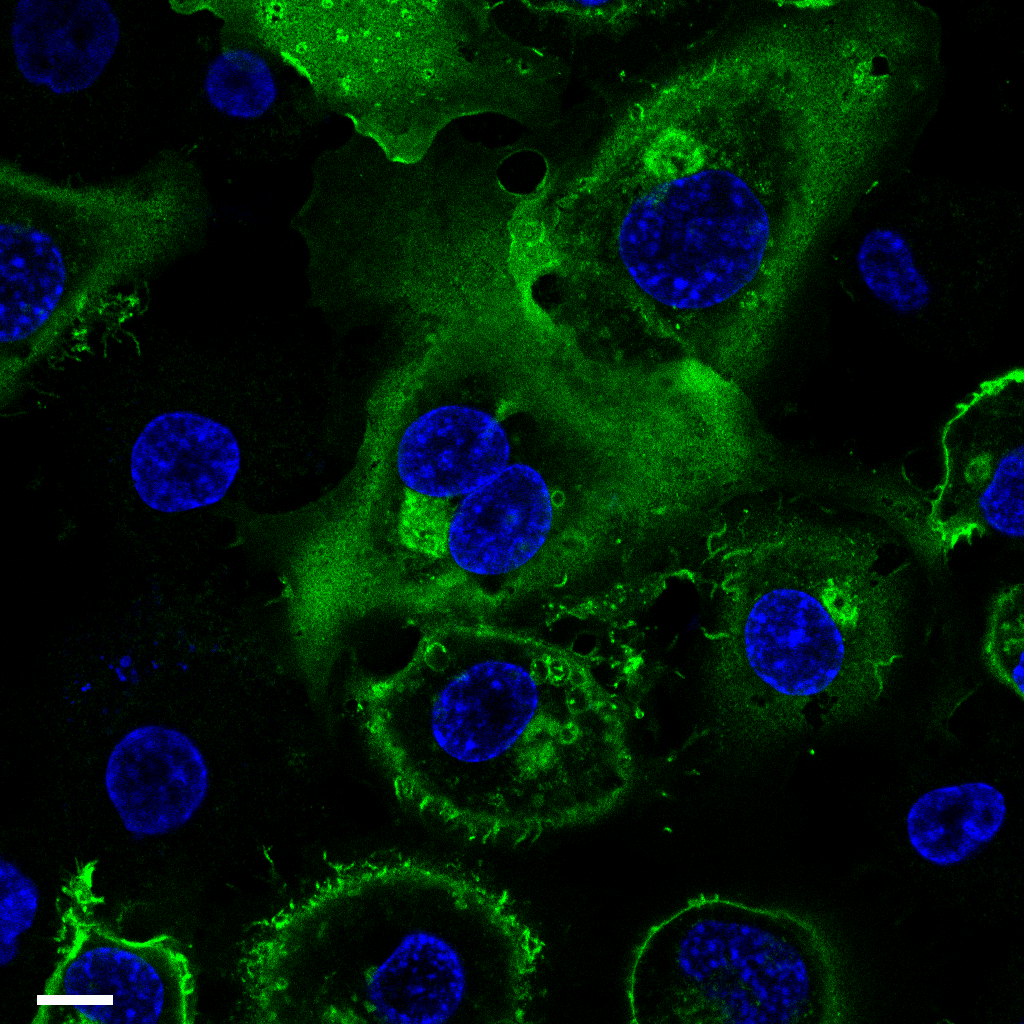

Supplement: Supplementary file 5 — Source data Fig. 3 [file 44319_2025_388_MOESM5_ESM.zip › Figure 3/3A_Merge_Perm_D85A.tif]

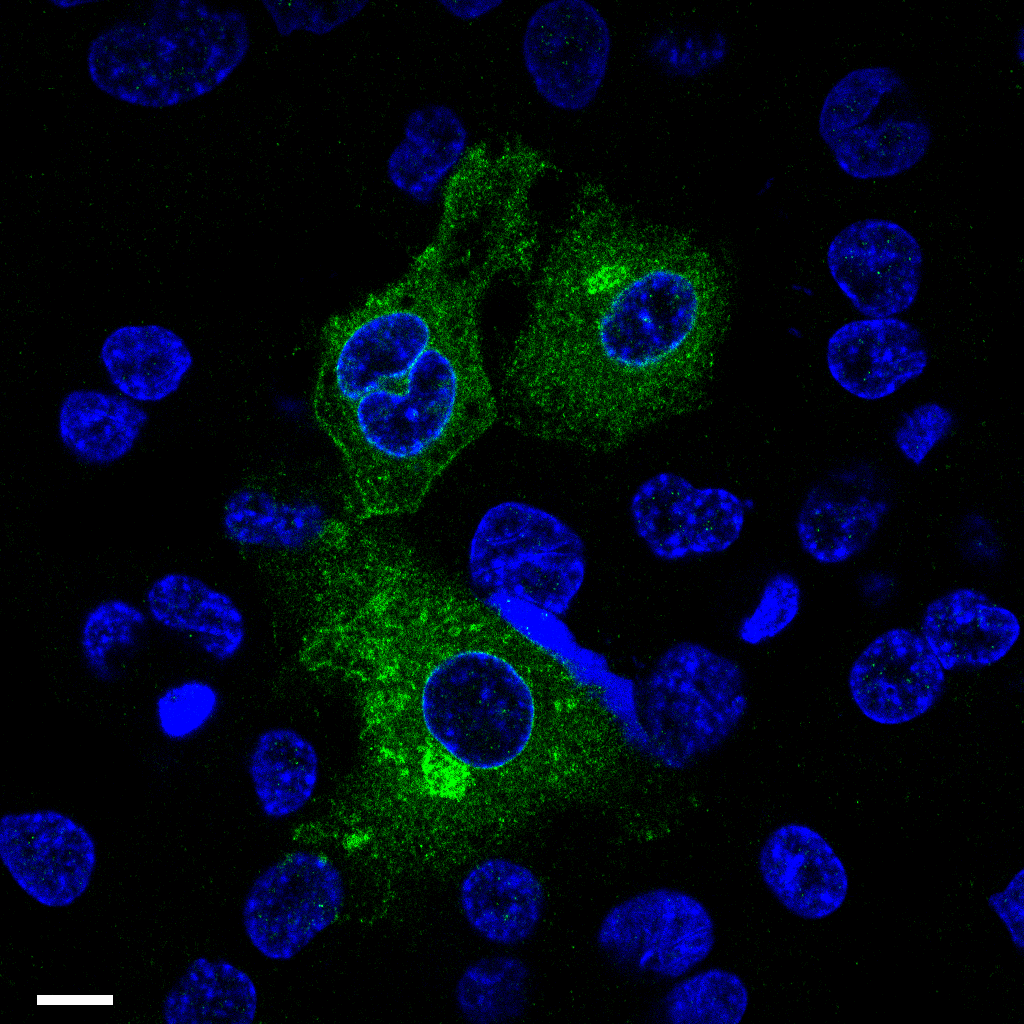

Supplement: Supplementary file 5 — Source data Fig. 3 [file 44319_2025_388_MOESM5_ESM.zip › Figure 3/3A_Merge_Perm_delta8697.tif]

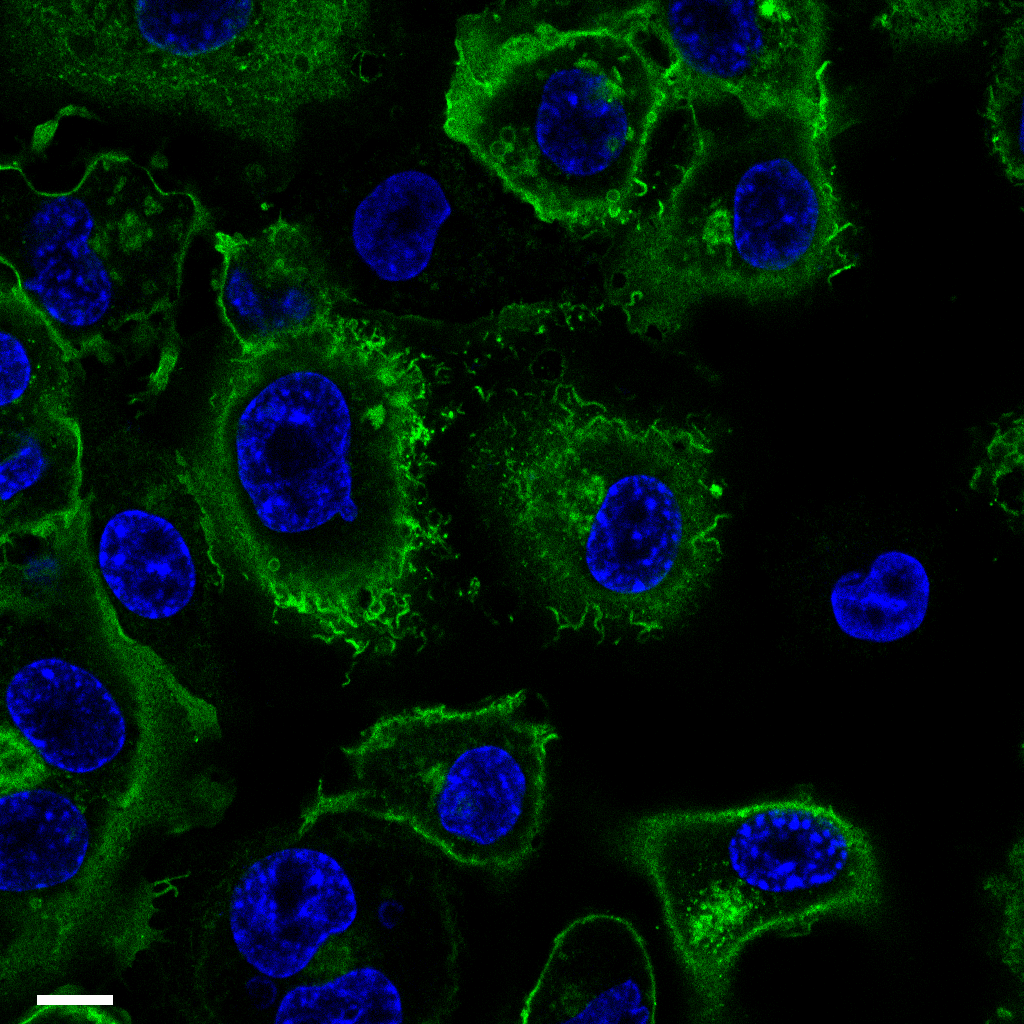

Supplement: Supplementary file 5 — Source data Fig. 3 [file 44319_2025_388_MOESM5_ESM.zip › Figure 3/3A_Merge_Perm_WT.tif]

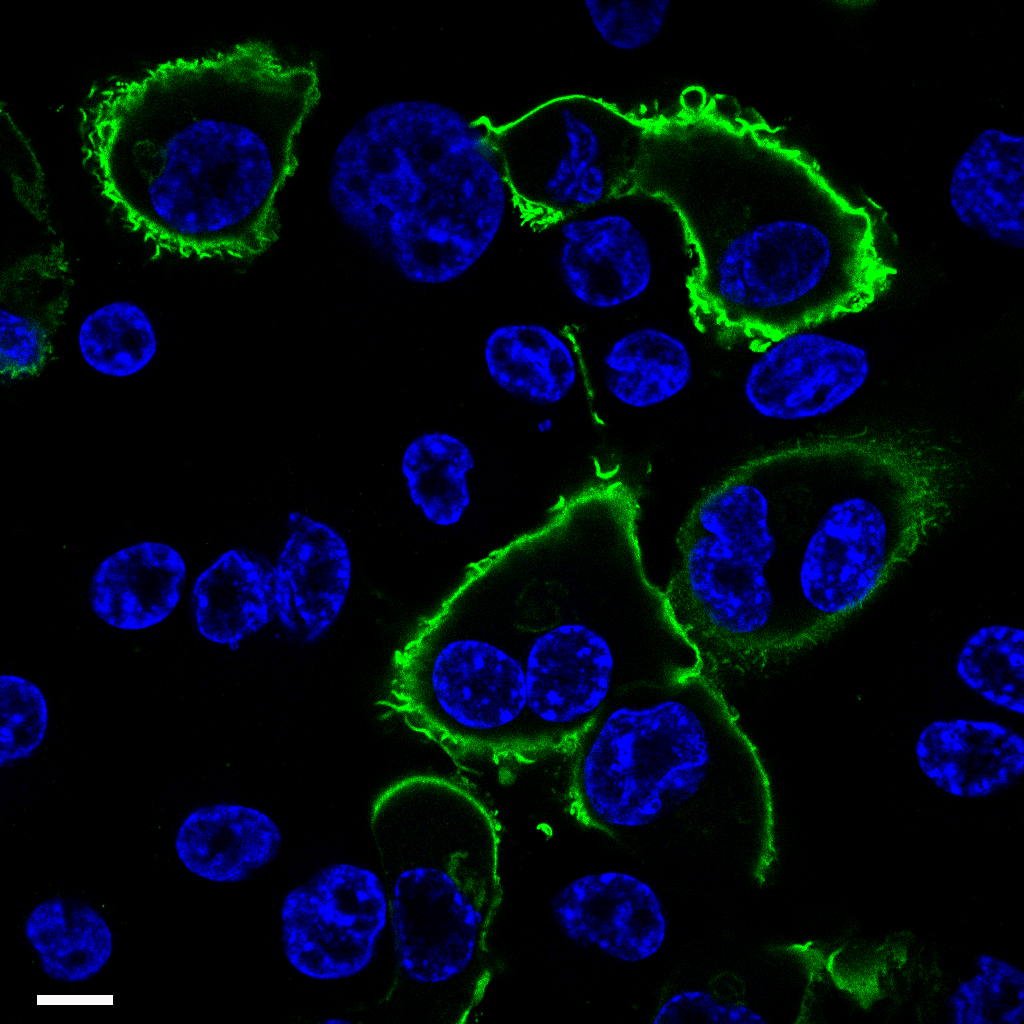

Supplement: Supplementary file 5 — Source data Fig. 3 [file 44319_2025_388_MOESM5_ESM.zip › Figure 3/3A_Merge_Surf_D85A.tif]

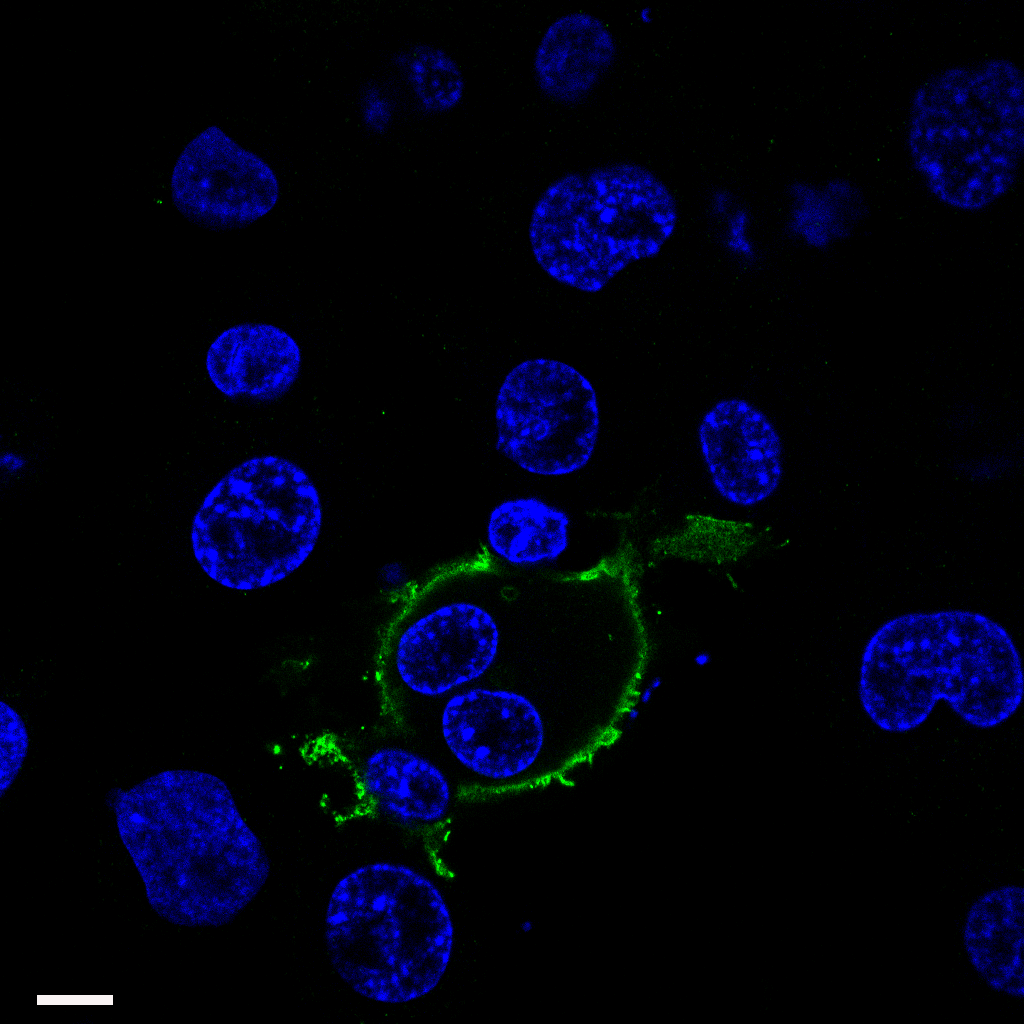

Supplement: Supplementary file 5 — Source data Fig. 3 [file 44319_2025_388_MOESM5_ESM.zip › Figure 3/3A_Merge_Surf_delta8697.tif]

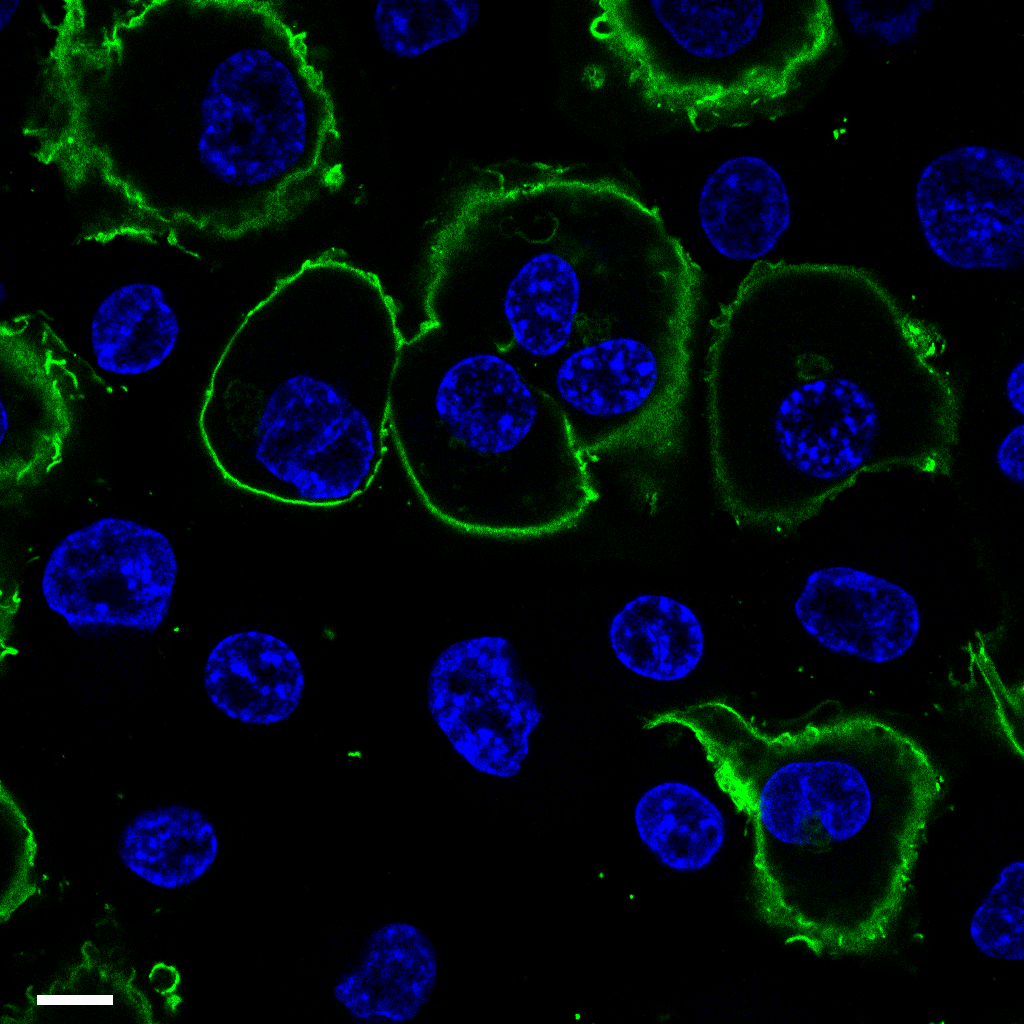

Supplement: Supplementary file 5 — Source data Fig. 3 [file 44319_2025_388_MOESM5_ESM.zip › Figure 3/3A_Merge_Surf_WT.tif]

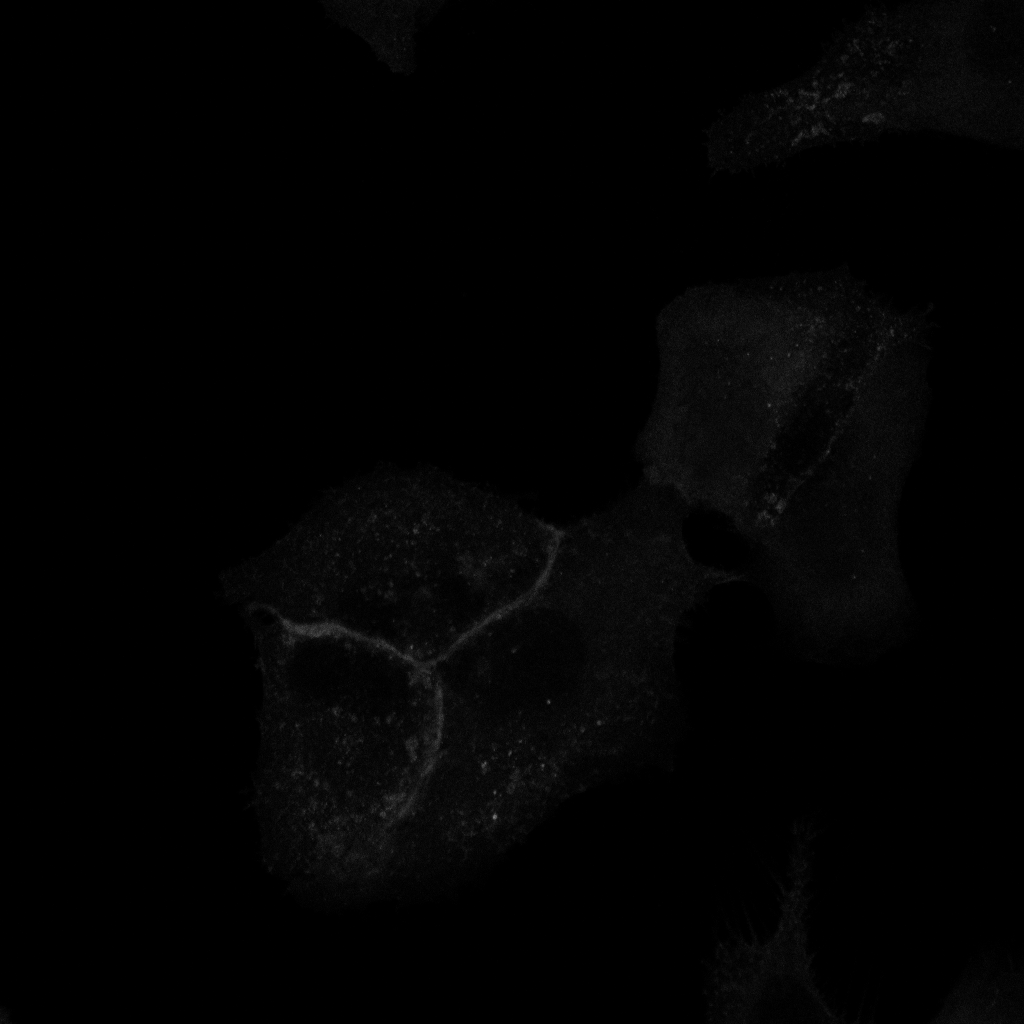

Supplement: Supplementary file 5 — Source data Fig. 3 [file 44319_2025_388_MOESM5_ESM.zip › Figure 3/3C_GFPLC3_Perm_D85A.tif]

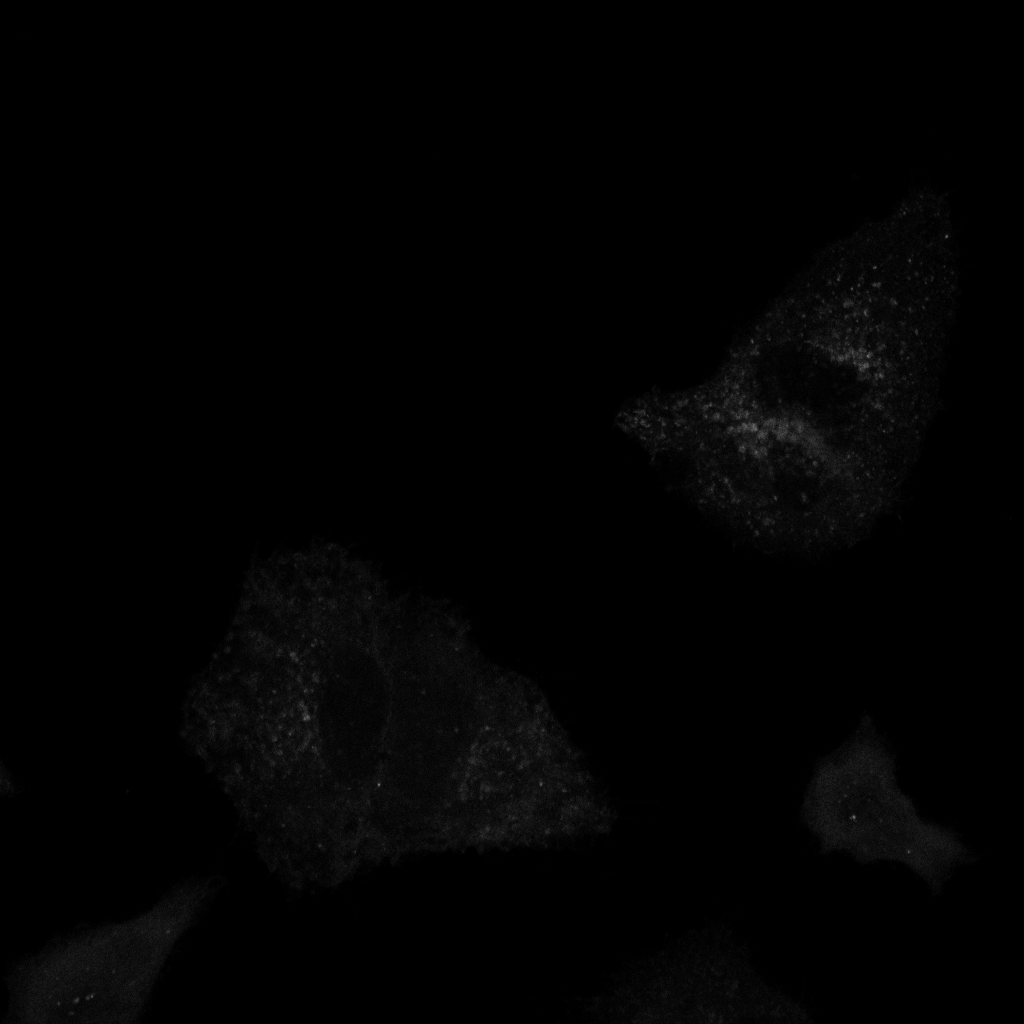

Supplement: Supplementary file 5 — Source data Fig. 3 [file 44319_2025_388_MOESM5_ESM.zip › Figure 3/3C_GFPLC3_Perm_delta8697.tif]

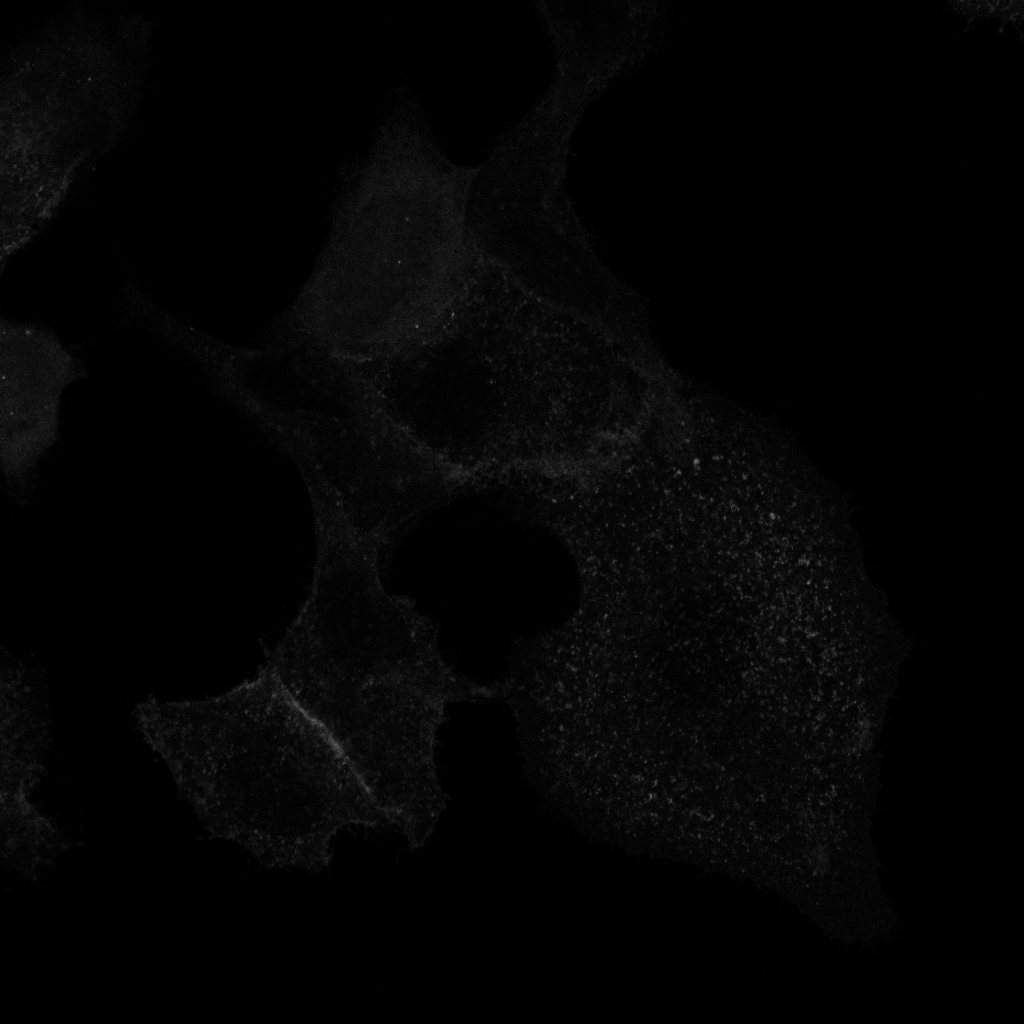

Supplement: Supplementary file 5 — Source data Fig. 3 [file 44319_2025_388_MOESM5_ESM.zip › Figure 3/3C_GFPLC3_Perm_WT.tif]

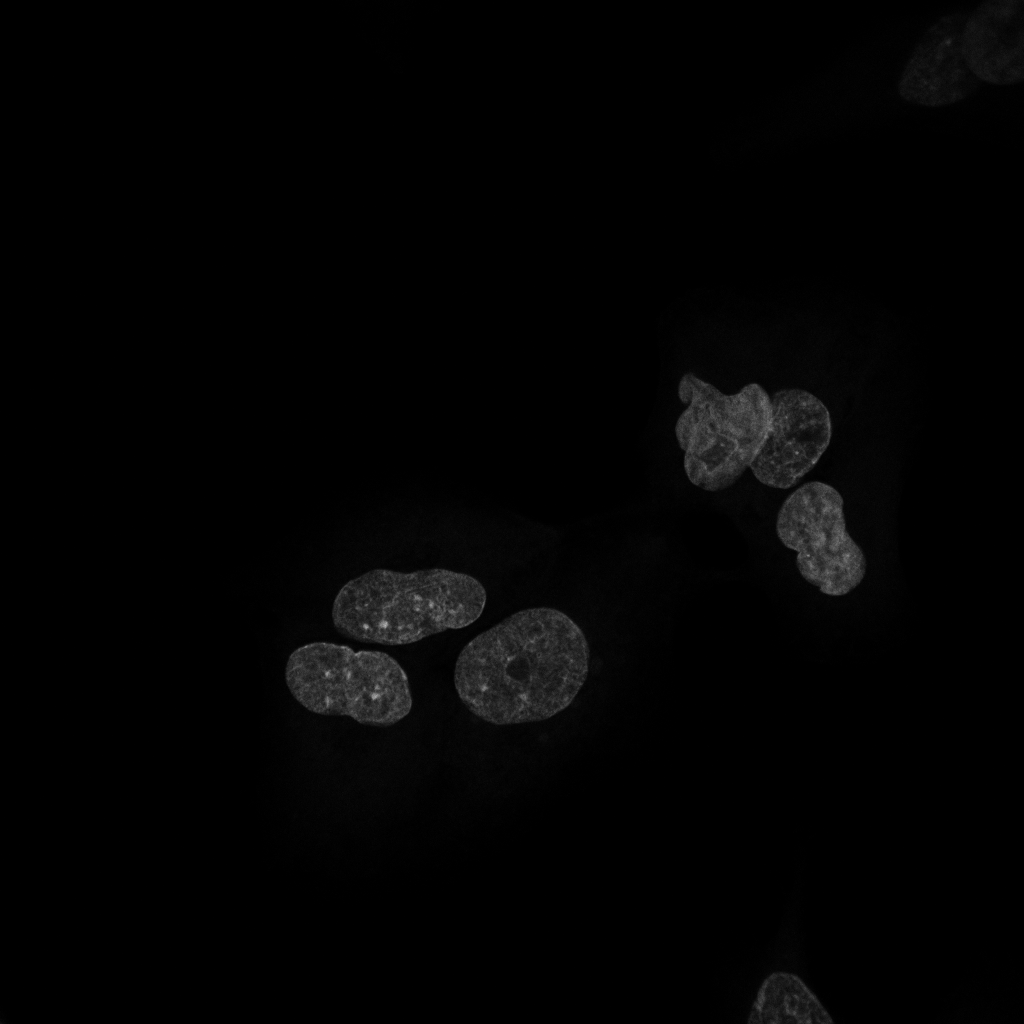

Supplement: Supplementary file 5 — Source data Fig. 3 [file 44319_2025_388_MOESM5_ESM.zip › Figure 3/3C_Hoechst_Perm_D85A.tif]

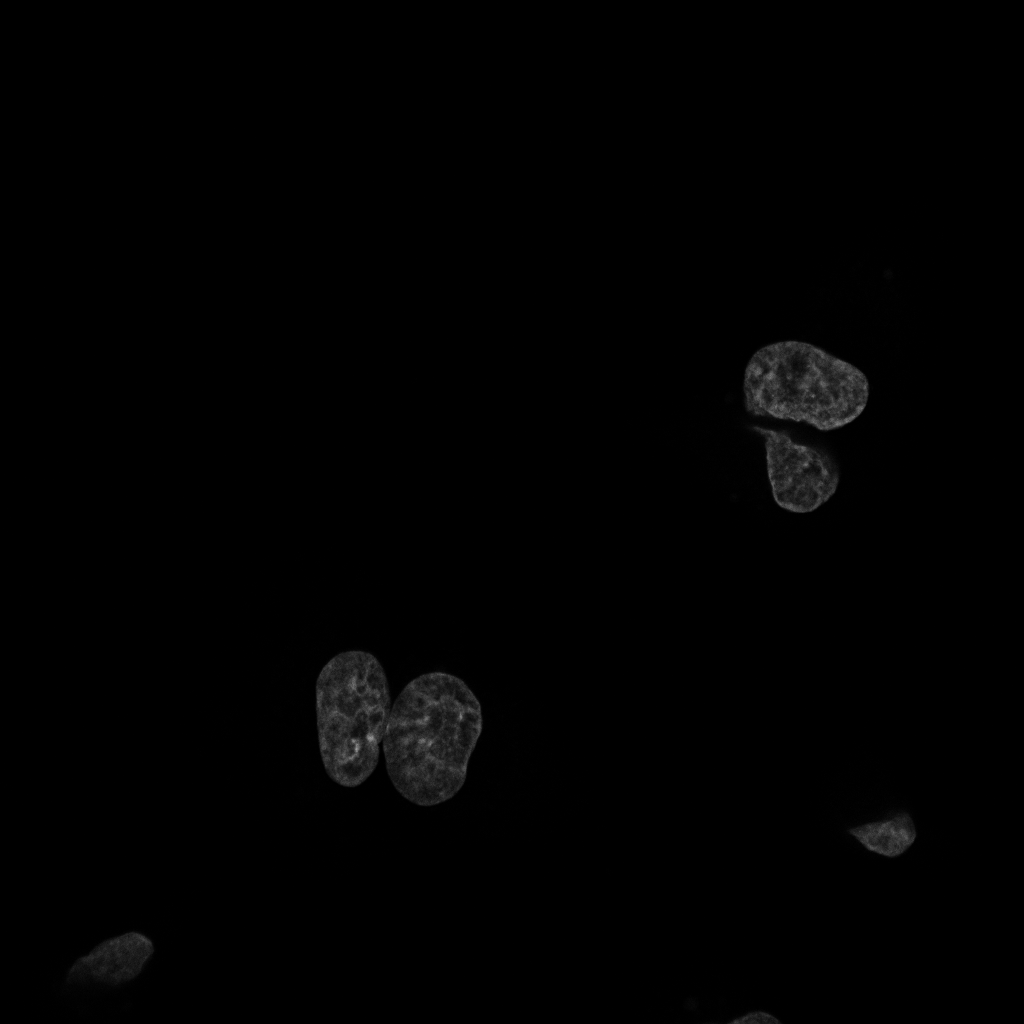

Supplement: Supplementary file 5 — Source data Fig. 3 [file 44319_2025_388_MOESM5_ESM.zip › Figure 3/3C_Hoechst_Perm_delta8697.tif]

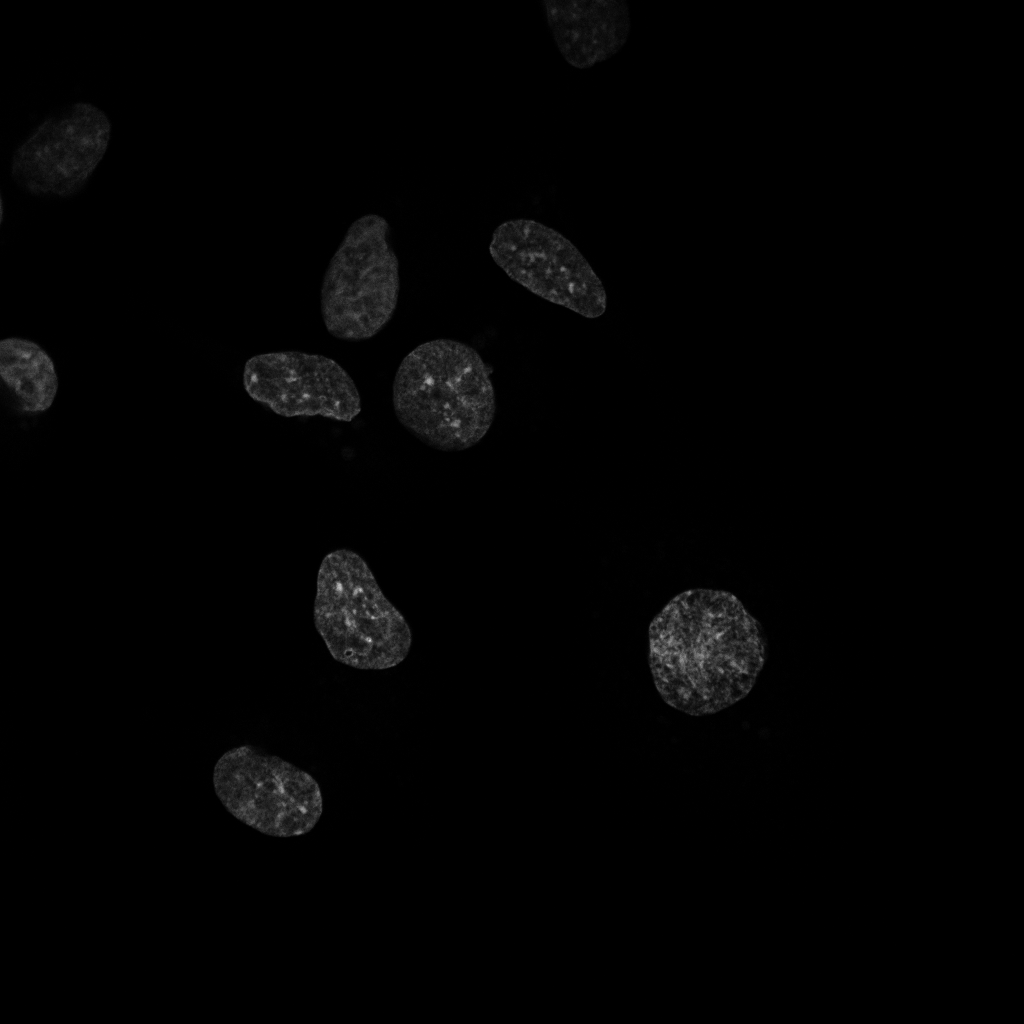

Supplement: Supplementary file 5 — Source data Fig. 3 [file 44319_2025_388_MOESM5_ESM.zip › Figure 3/3C_Hoechst_Perm_WT.tif]

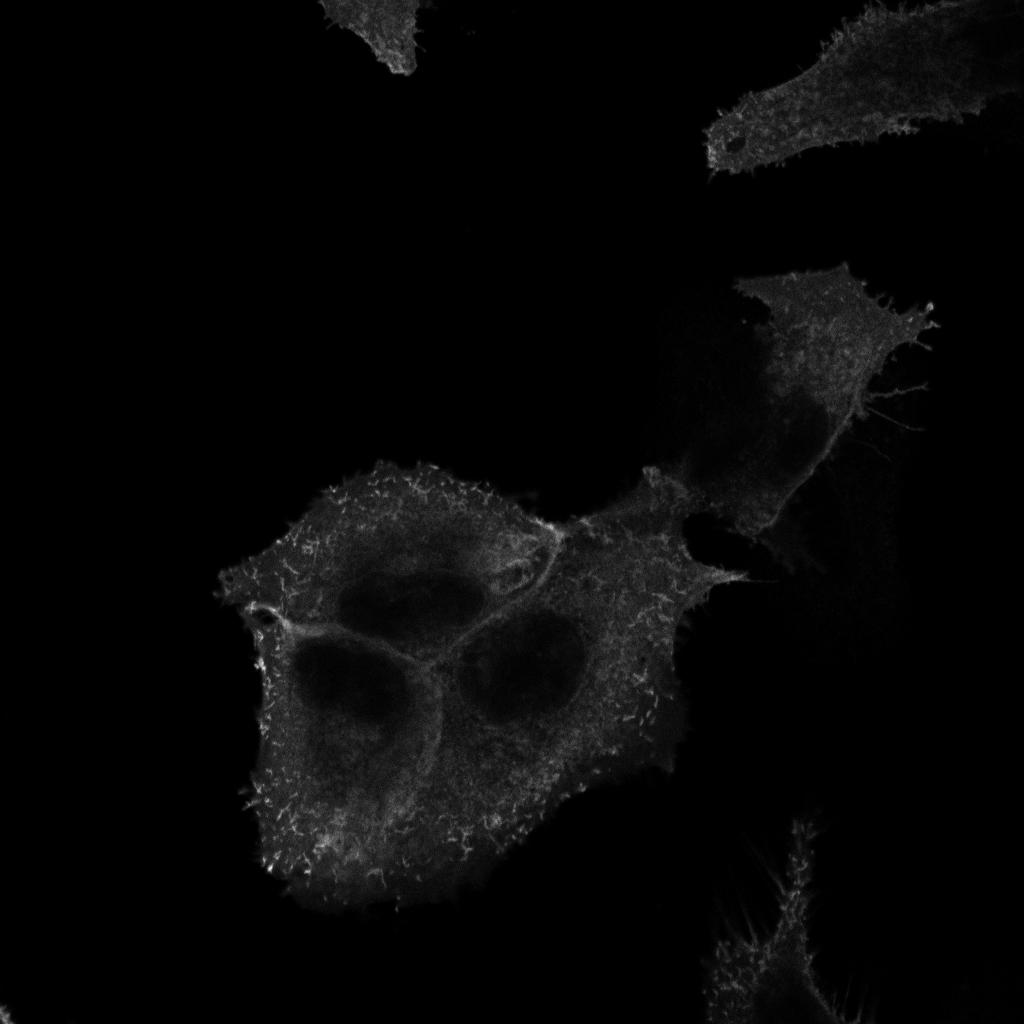

Supplement: Supplementary file 5 — Source data Fig. 3 [file 44319_2025_388_MOESM5_ESM.zip › Figure 3/3C_M2_Perm_D85A.tif]

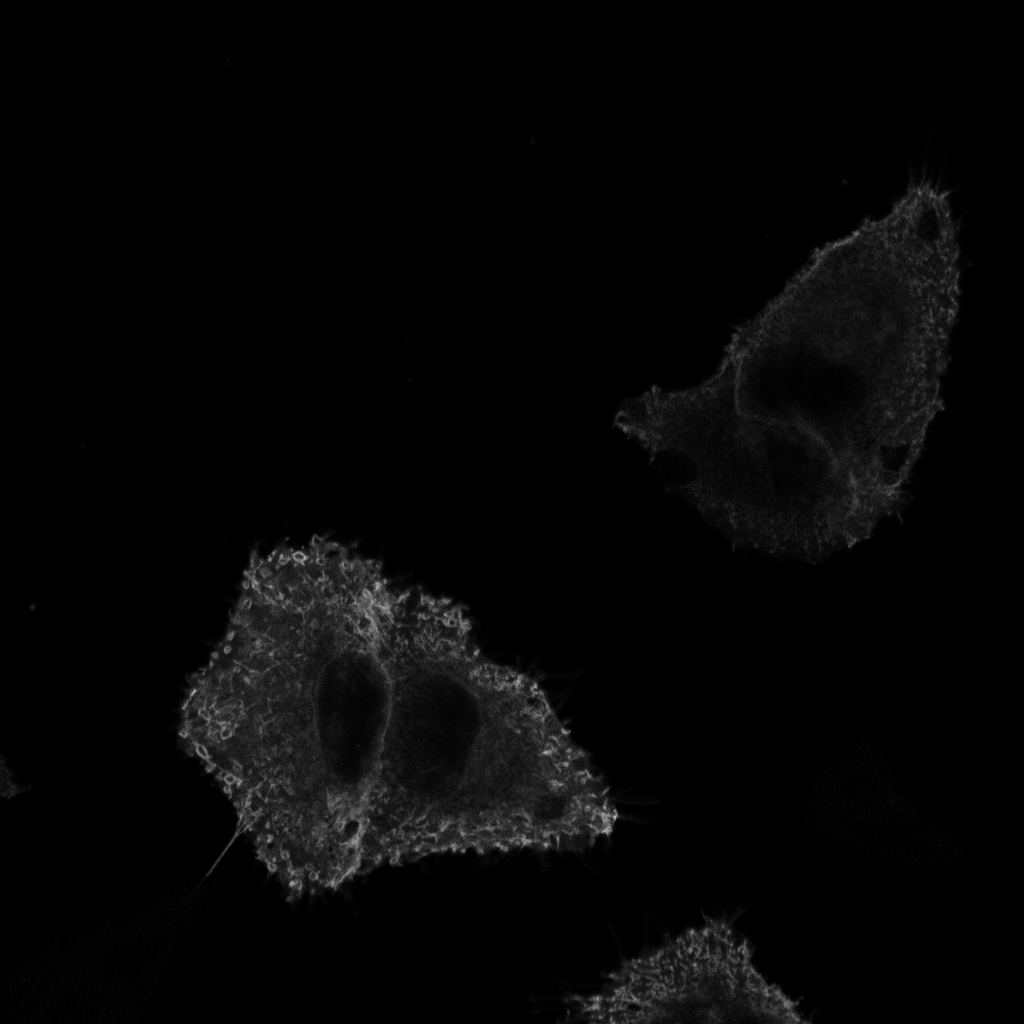

Supplement: Supplementary file 5 — Source data Fig. 3 [file 44319_2025_388_MOESM5_ESM.zip › Figure 3/3C_M2_Perm_delta8697.tif]

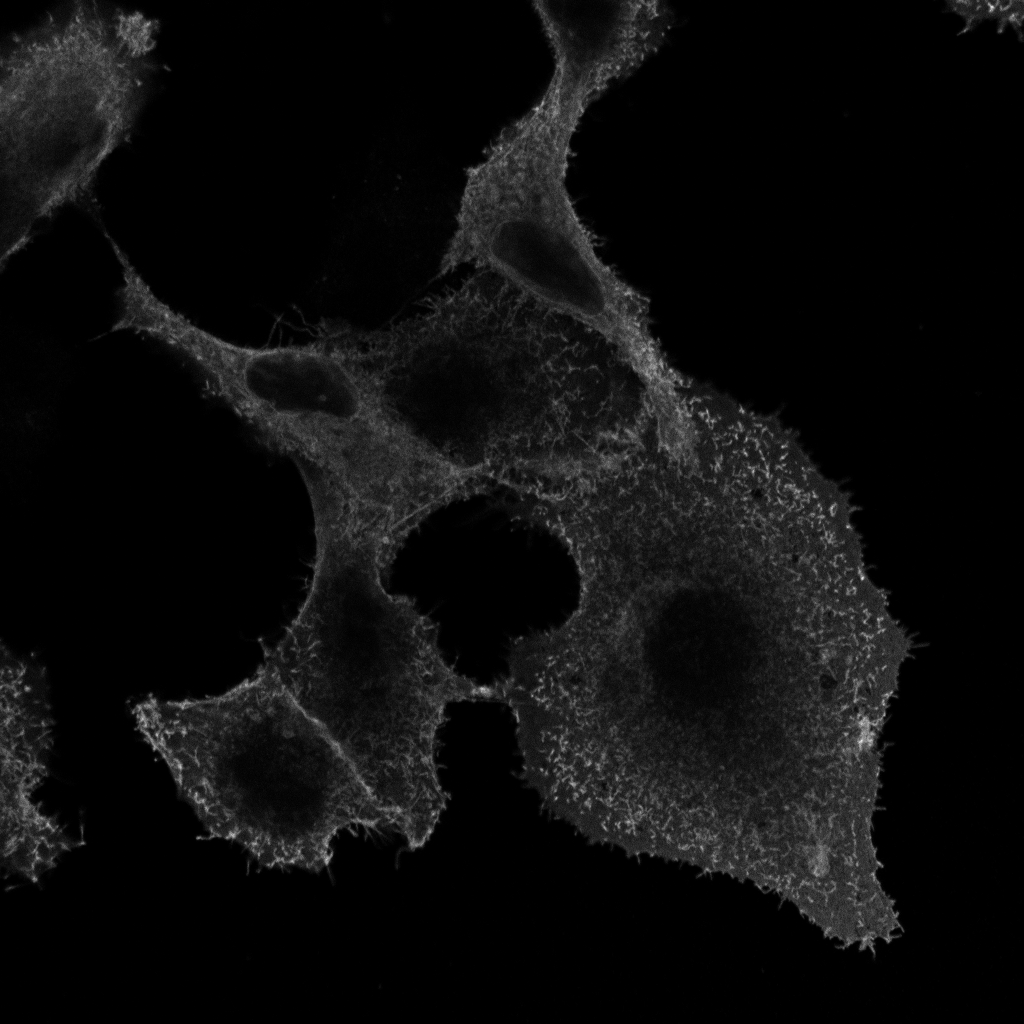

Supplement: Supplementary file 5 — Source data Fig. 3 [file 44319_2025_388_MOESM5_ESM.zip › Figure 3/3C_M2_Perm_WT.tif]

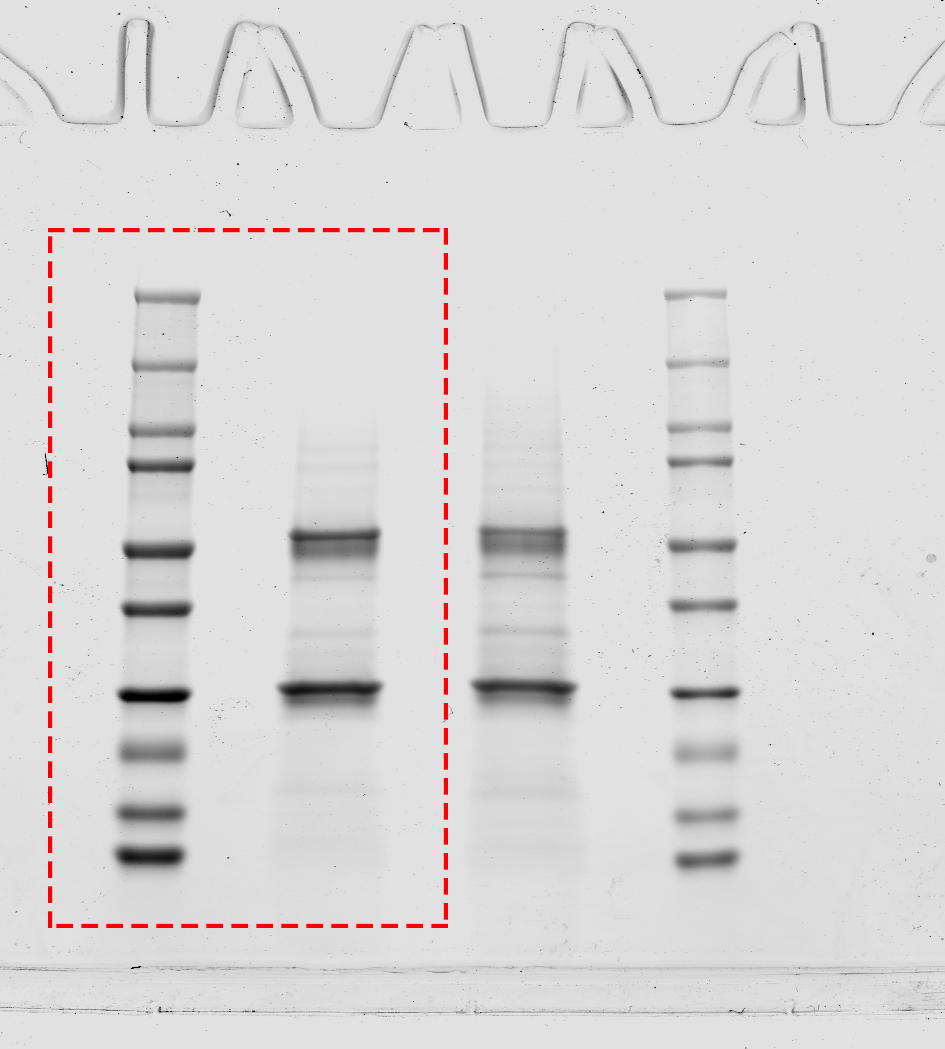

Supplement: Supplementary file 6 — Source data Fig. 4 [file 44319_2025_388_MOESM6_ESM.zip › Figure 4/4A_purification_coomassie_A.tif]

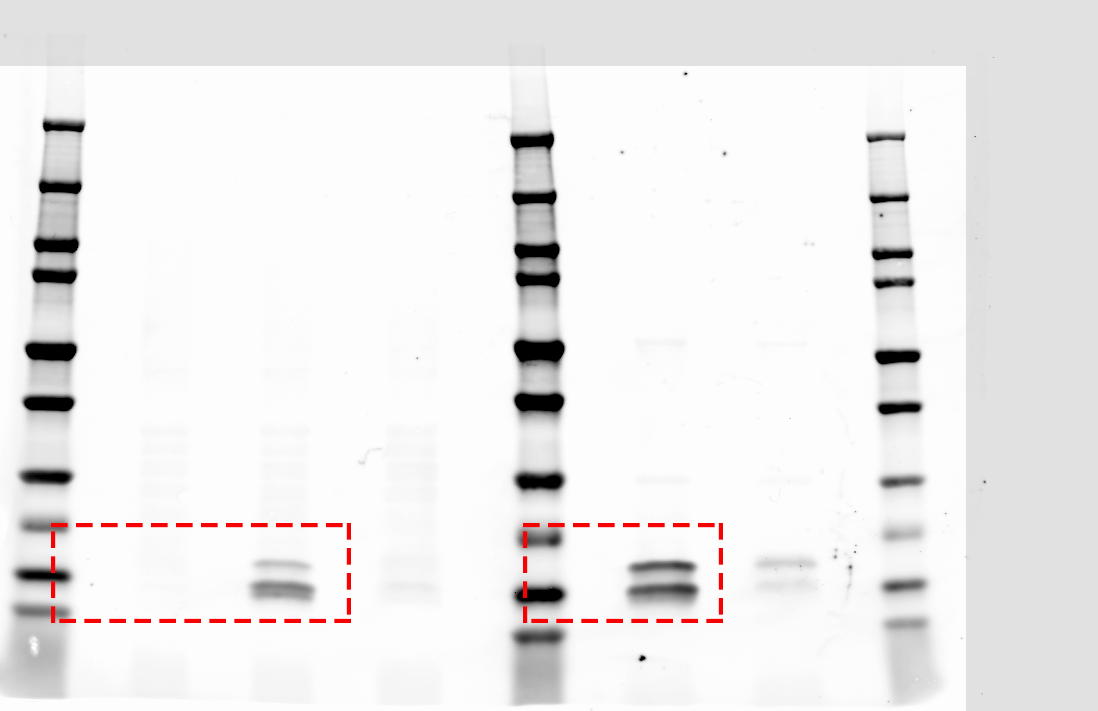

Supplement: Supplementary file 6 — Source data Fig. 4 [file 44319_2025_388_MOESM6_ESM.zip › Figure 4/4B_M2_ms700.tif]

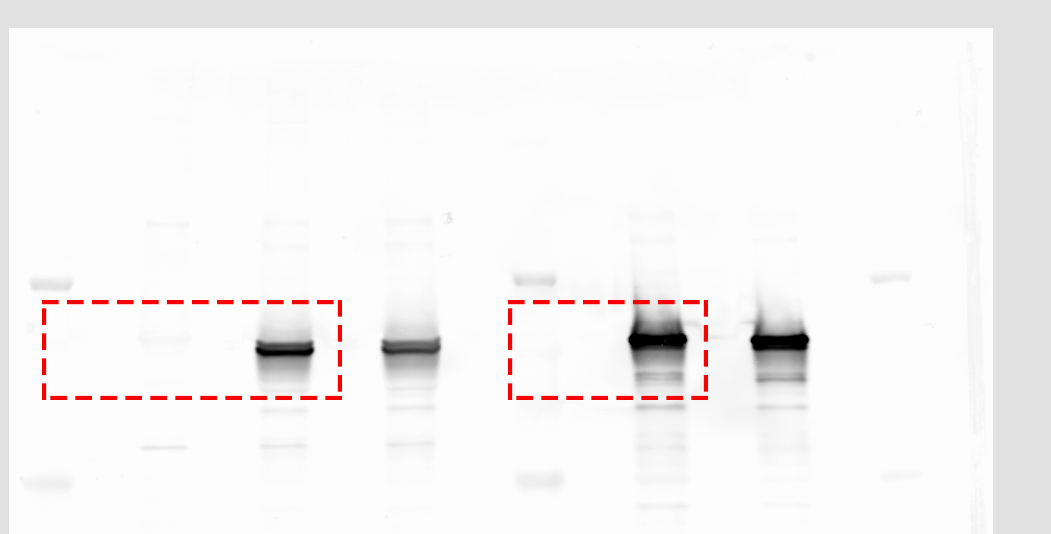

Supplement: Supplementary file 6 — Source data Fig. 4 [file 44319_2025_388_MOESM6_ESM.zip › Figure 4/4B_NP_rb800.tif]

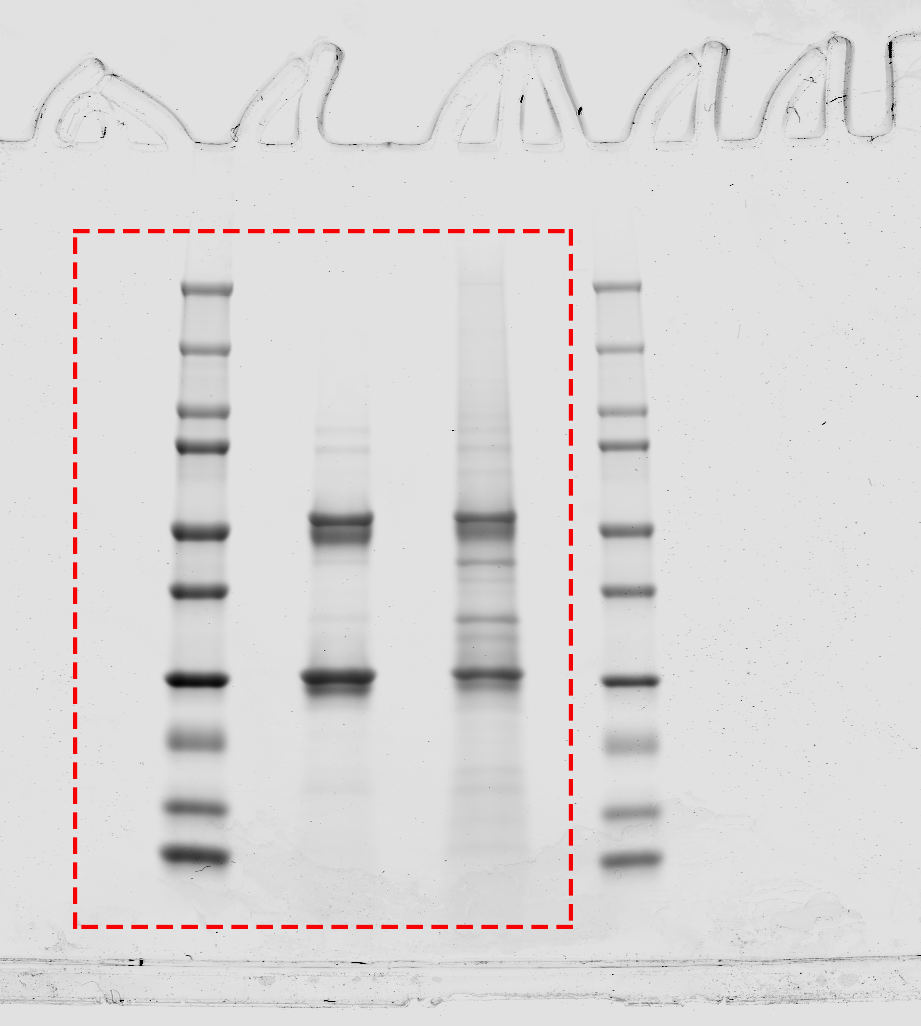

Supplement: Supplementary file 6 — Source data Fig. 4 [file 44319_2025_388_MOESM6_ESM.zip › Figure 4/4D_D85A_Trunc_coomassie1.2_A.tif]

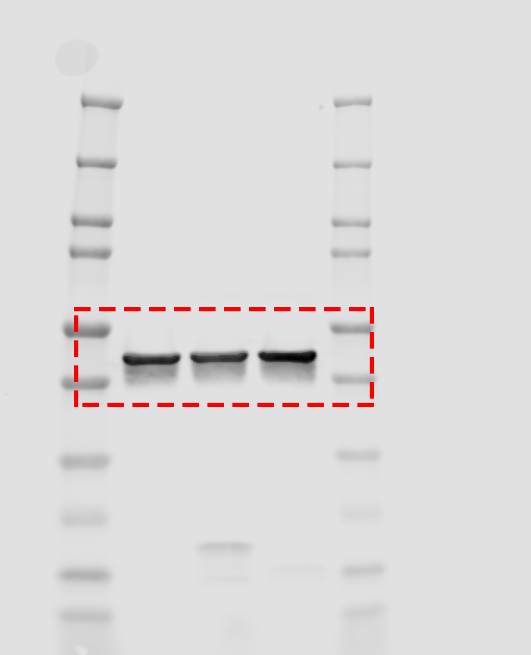

Supplement: Supplementary file 6 — Source data Fig. 4 [file 44319_2025_388_MOESM6_ESM.zip › Figure 4/4E_CellLysate_actin_ms700.tif]

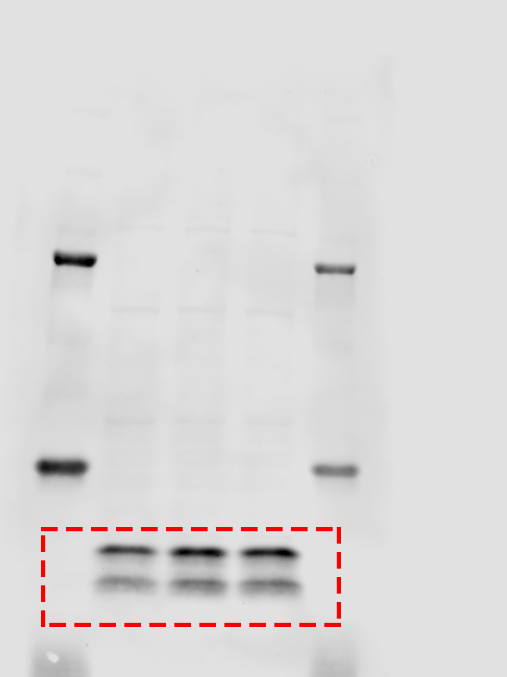

Supplement: Supplementary file 6 — Source data Fig. 4 [file 44319_2025_388_MOESM6_ESM.zip › Figure 4/4E_CellLysate_LC3B_rb800.tif]

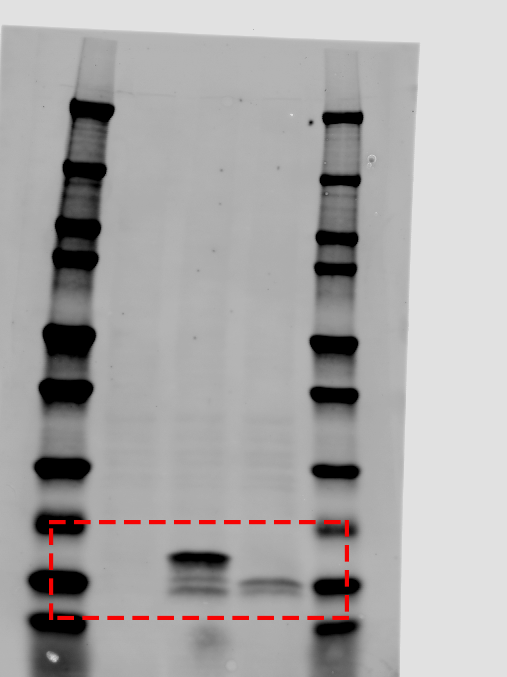

Supplement: Supplementary file 6 — Source data Fig. 4 [file 44319_2025_388_MOESM6_ESM.zip › Figure 4/4E_CellLysate_M2_ms700.tif]

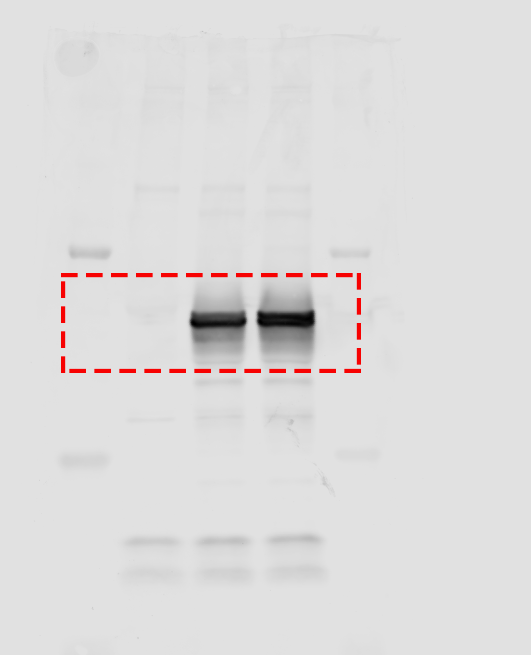

Supplement: Supplementary file 6 — Source data Fig. 4 [file 44319_2025_388_MOESM6_ESM.zip › Figure 4/4E_CellLysate_NP_rb800.tif]

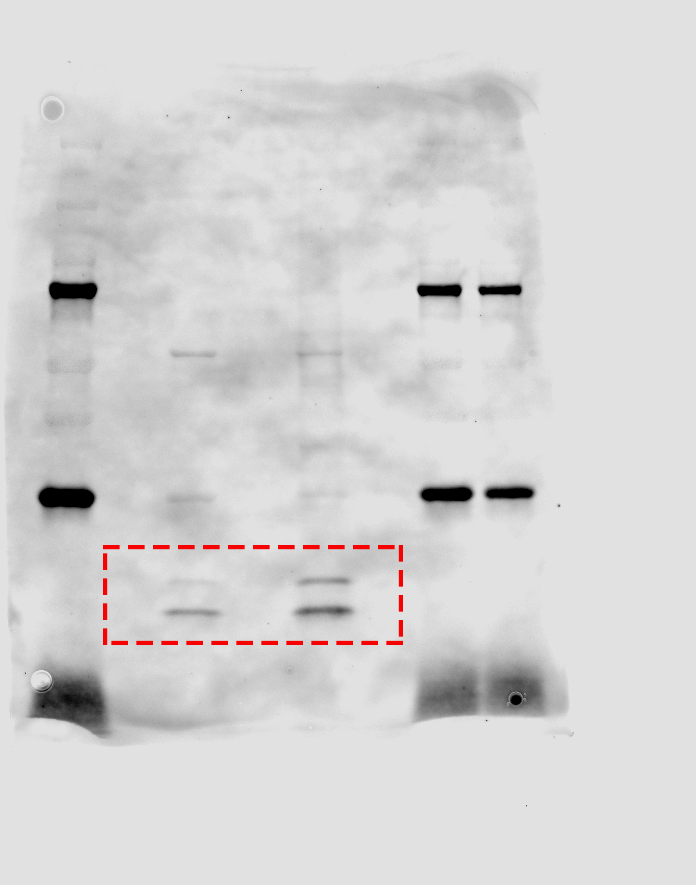

Supplement: Supplementary file 6 — Source data Fig. 4 [file 44319_2025_388_MOESM6_ESM.zip › Figure 4/4E_D85A_Trunc_LC3B_rb800.tif]

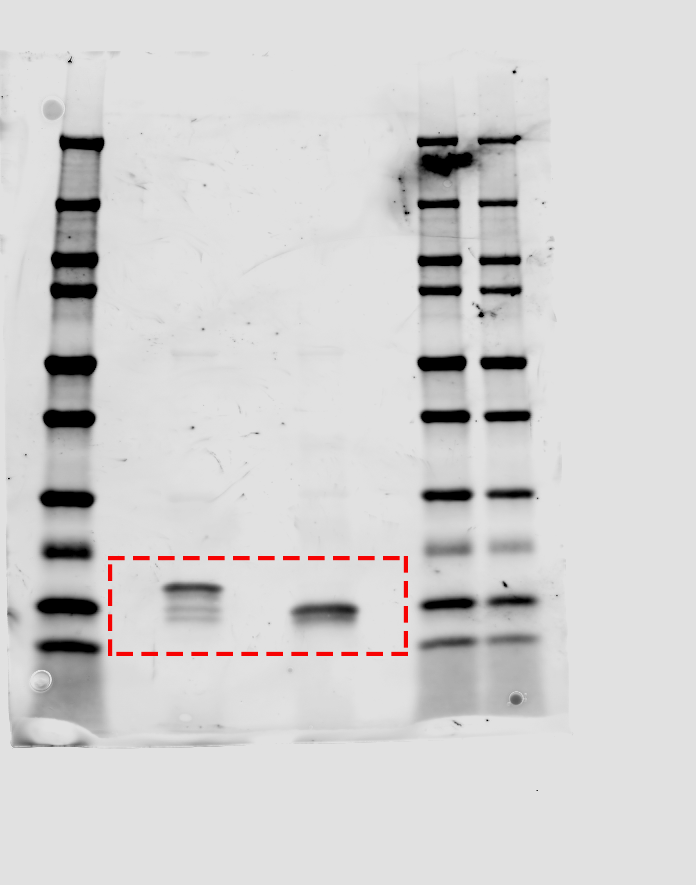

Supplement: Supplementary file 6 — Source data Fig. 4 [file 44319_2025_388_MOESM6_ESM.zip › Figure 4/4E_D85A_Trunc_M2_ms700.tif]

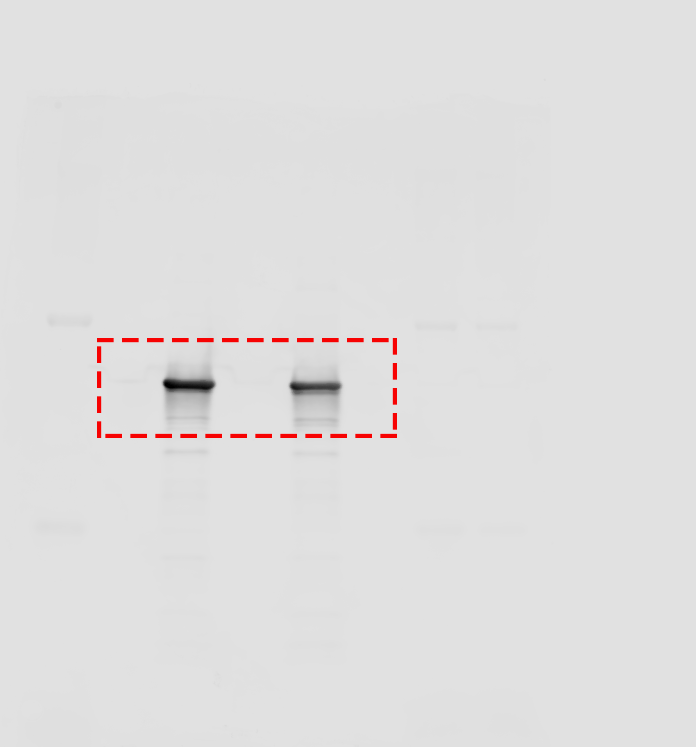

Supplement: Supplementary file 6 — Source data Fig. 4 [file 44319_2025_388_MOESM6_ESM.zip › Figure 4/4E_D85A_Trunc_NP_rb800.tif]

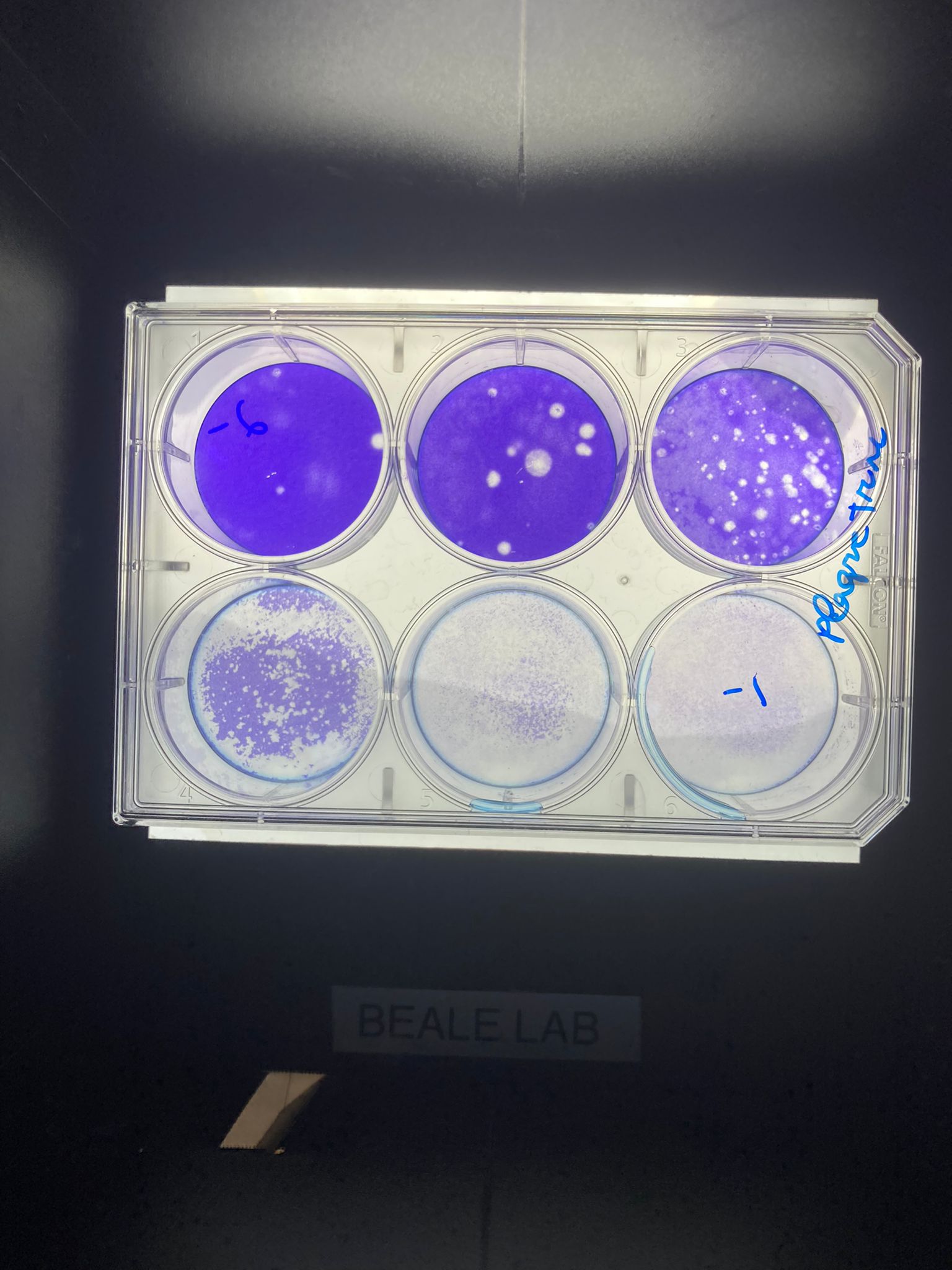

Supplement: Supplementary file 7 — Source data Fig. 5 [file 44319_2025_388_MOESM7_ESM.zip › Figure 5/5B_M2Delta86_97_whole plate.jpeg]

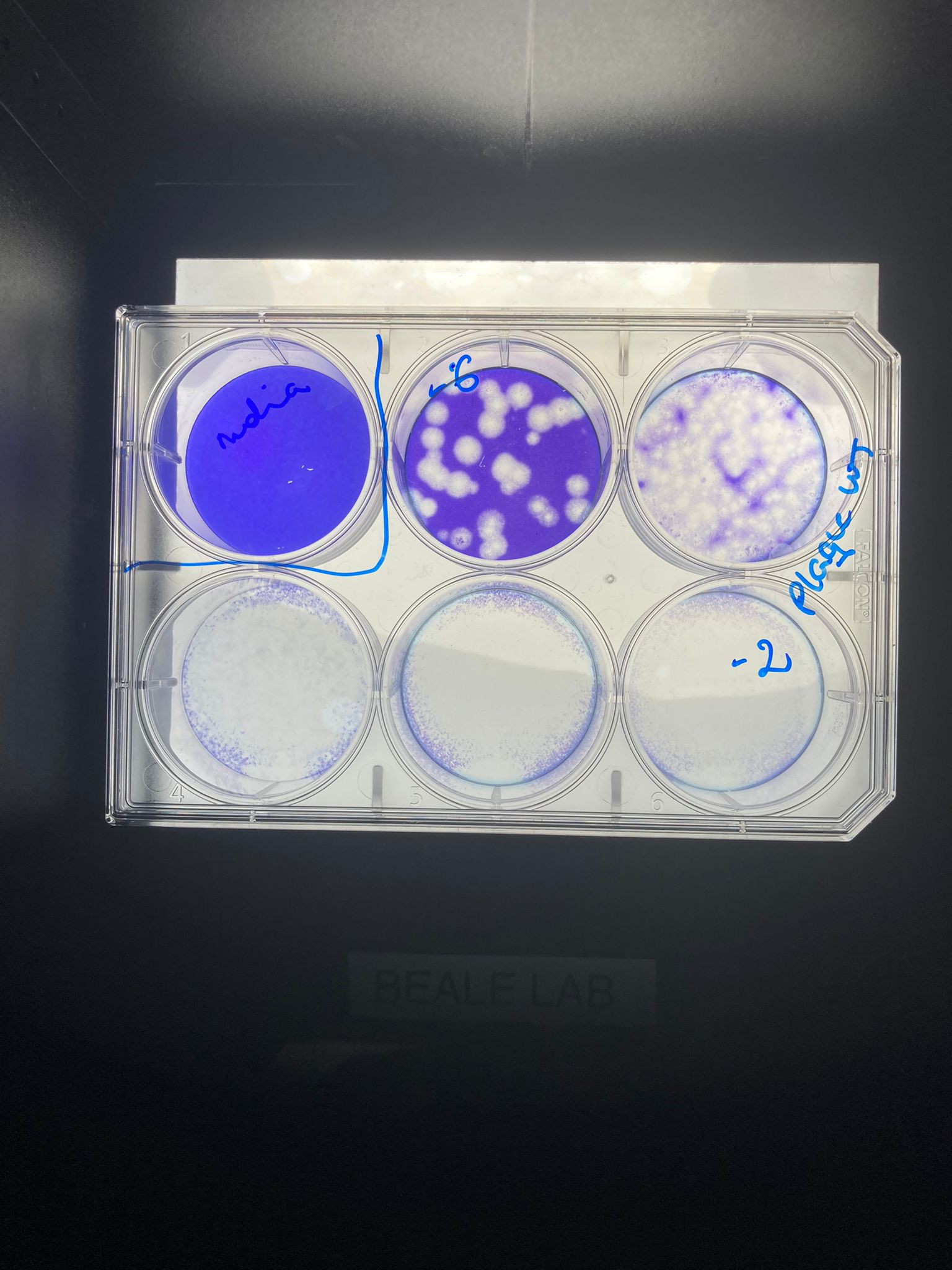

Supplement: Supplementary file 7 — Source data Fig. 5 [file 44319_2025_388_MOESM7_ESM.zip › Figure 5/5B_M2WT_whole plate.jpeg]

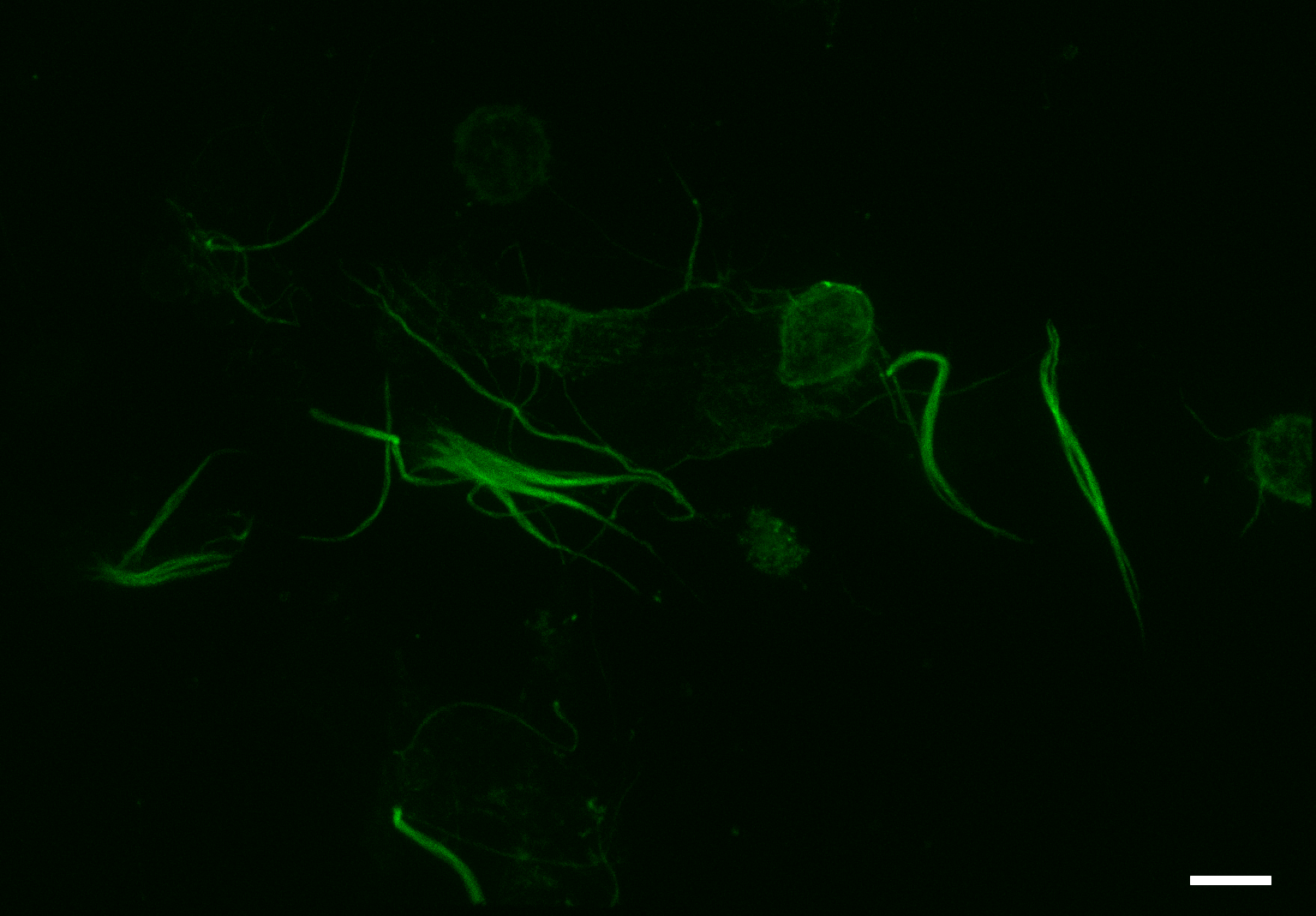

Supplement: Supplementary file 8 — Source data Fig. 6 [file 44319_2025_388_MOESM8_ESM.zip › Figure 6/6A_HA_D85A.tif]

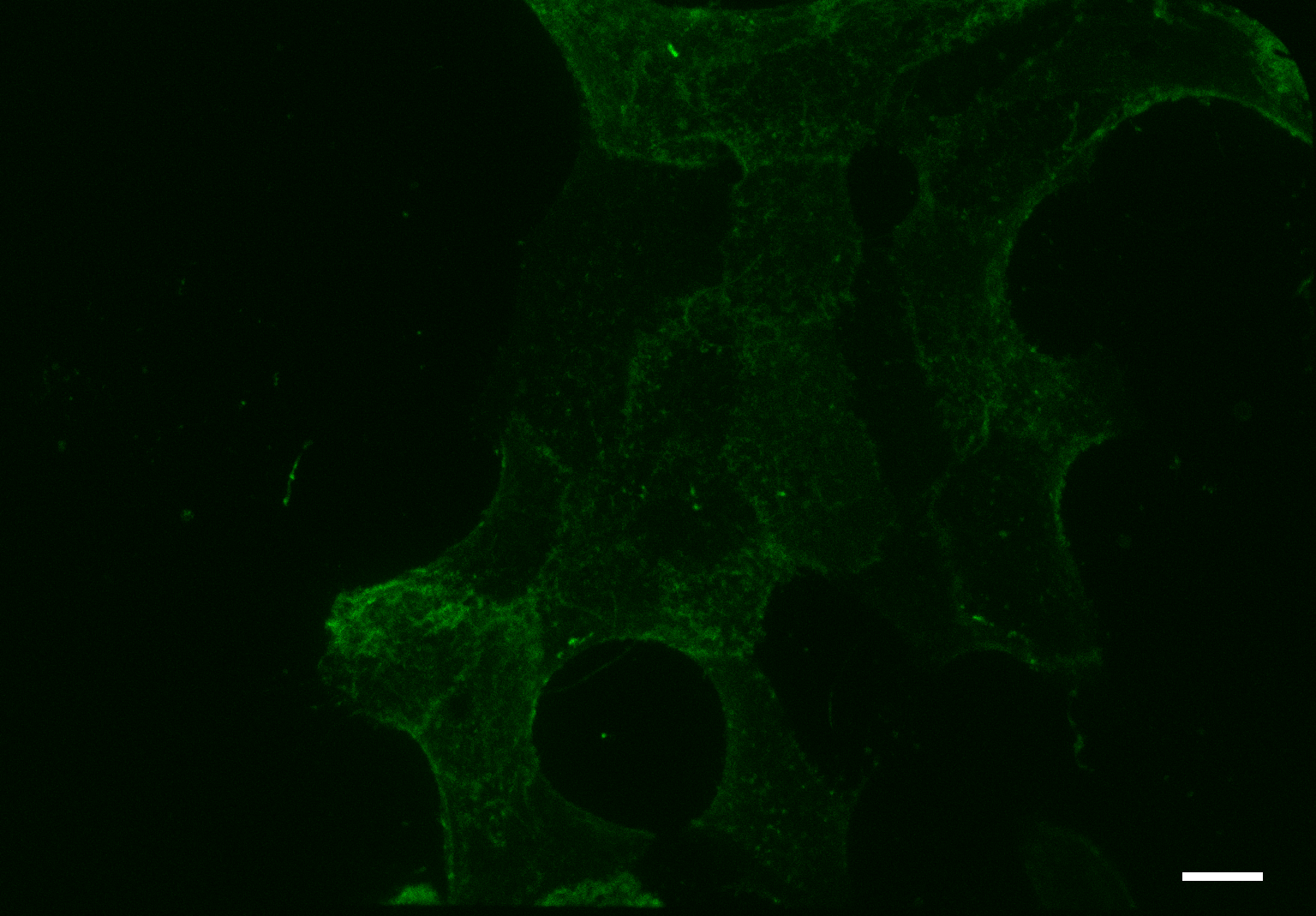

Supplement: Supplementary file 8 — Source data Fig. 6 [file 44319_2025_388_MOESM8_ESM.zip › Figure 6/6A_HA_delta8697_M2cells.tif]

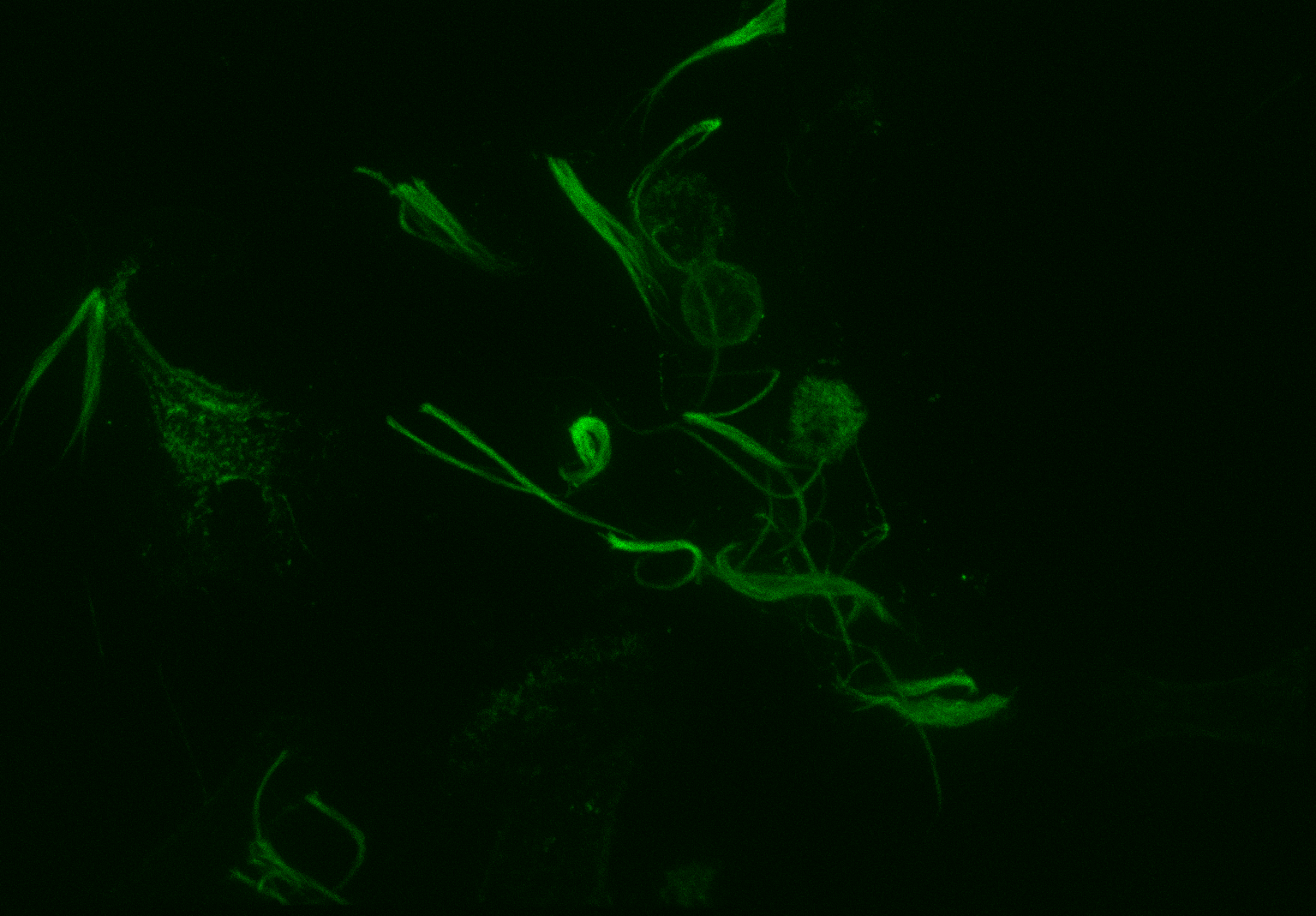

Supplement: Supplementary file 8 — Source data Fig. 6 [file 44319_2025_388_MOESM8_ESM.zip › Figure 6/6A_HA_WT.tif]

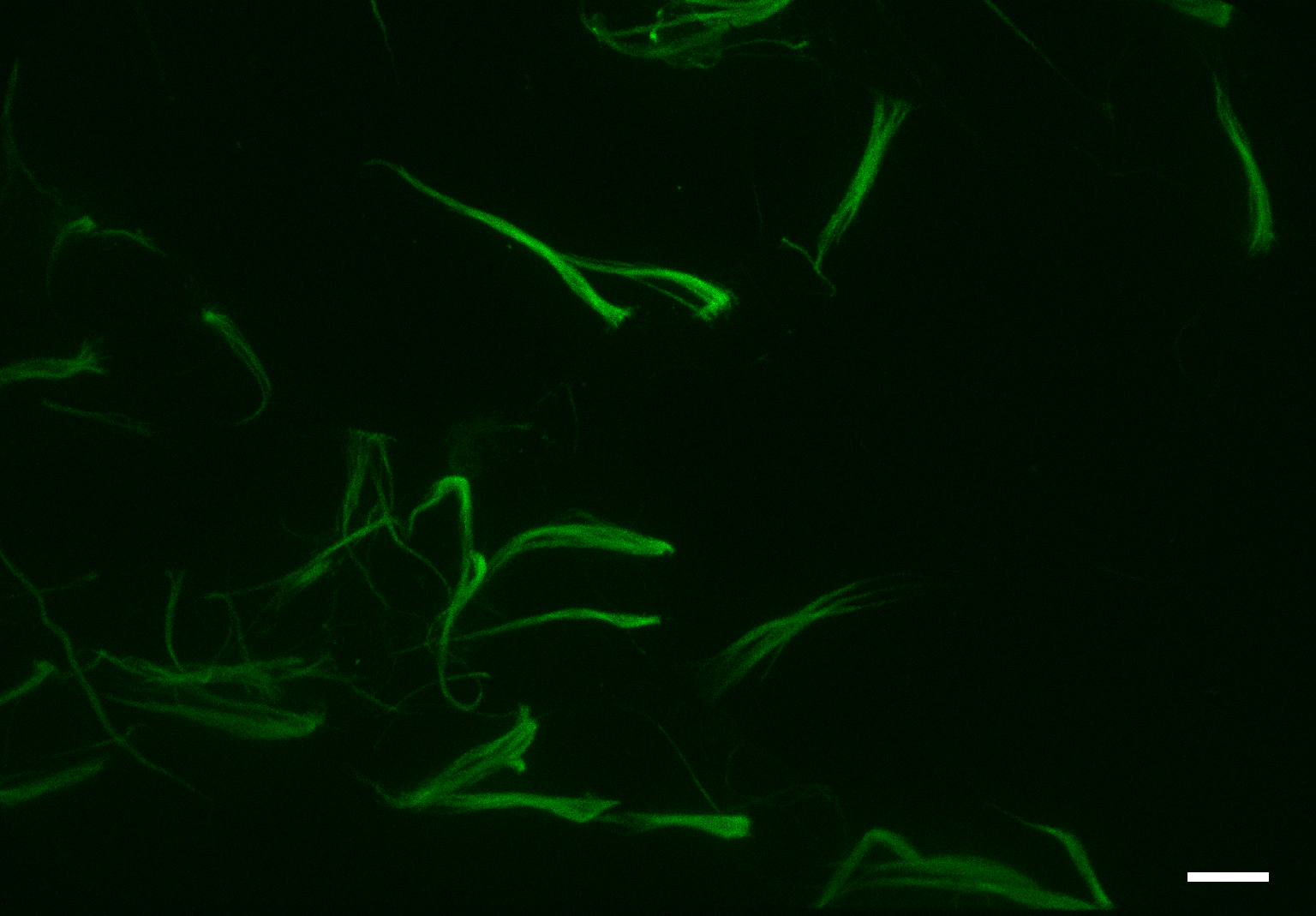

Supplement: Supplementary file 8 — Source data Fig. 6 [file 44319_2025_388_MOESM8_ESM.zip › Figure 6/6A_HA_WT_M2cells.tif]

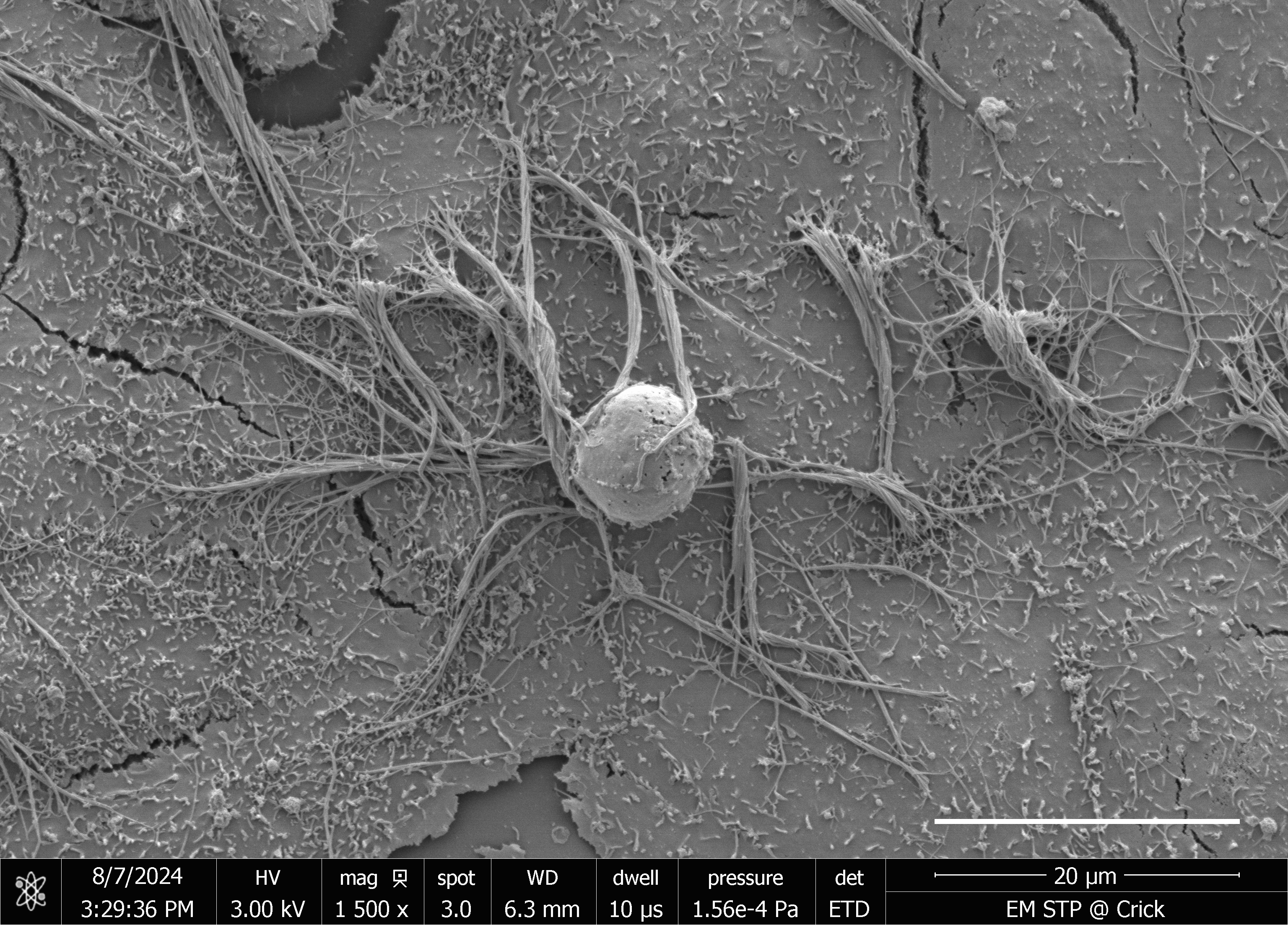

Supplement: Supplementary file 8 — Source data Fig. 6 [file 44319_2025_388_MOESM8_ESM.zip › Figure 6/6B_D85A_20sb.tif]

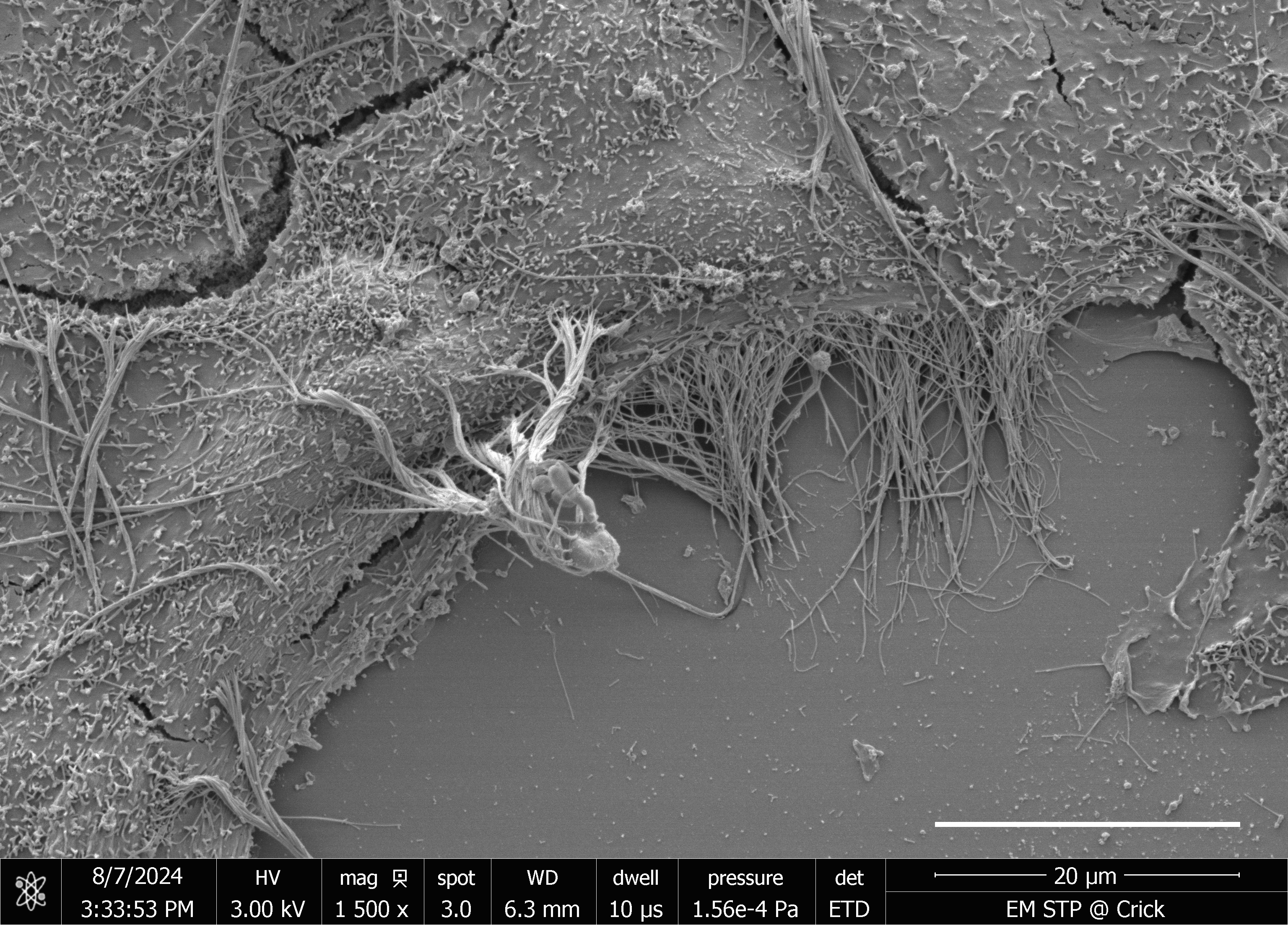

Supplement: Supplementary file 8 — Source data Fig. 6 [file 44319_2025_388_MOESM8_ESM.zip › Figure 6/6B_D85A_20sb2.tif]

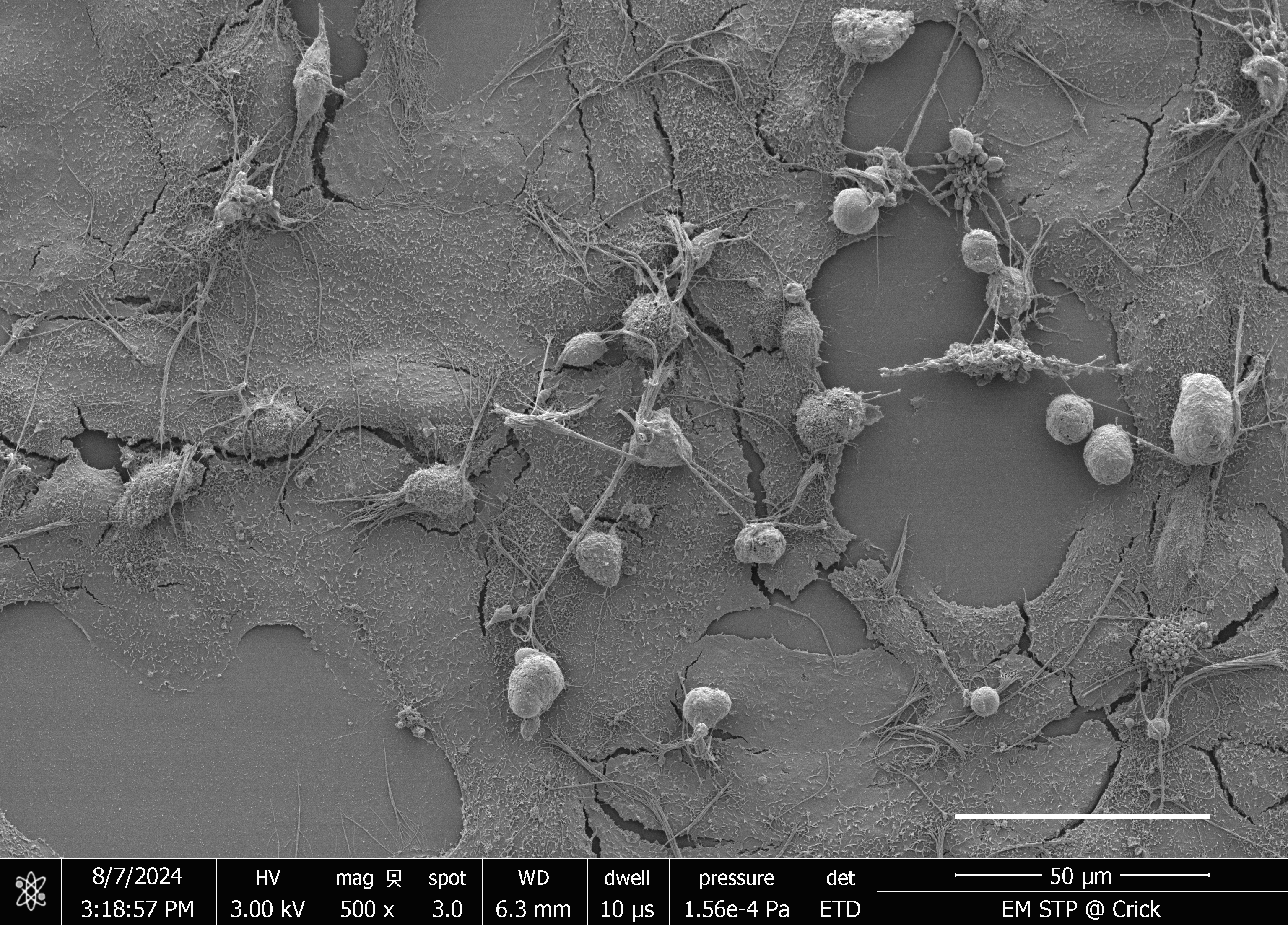

Supplement: Supplementary file 8 — Source data Fig. 6 [file 44319_2025_388_MOESM8_ESM.zip › Figure 6/6B_D85A_50sb.tif]

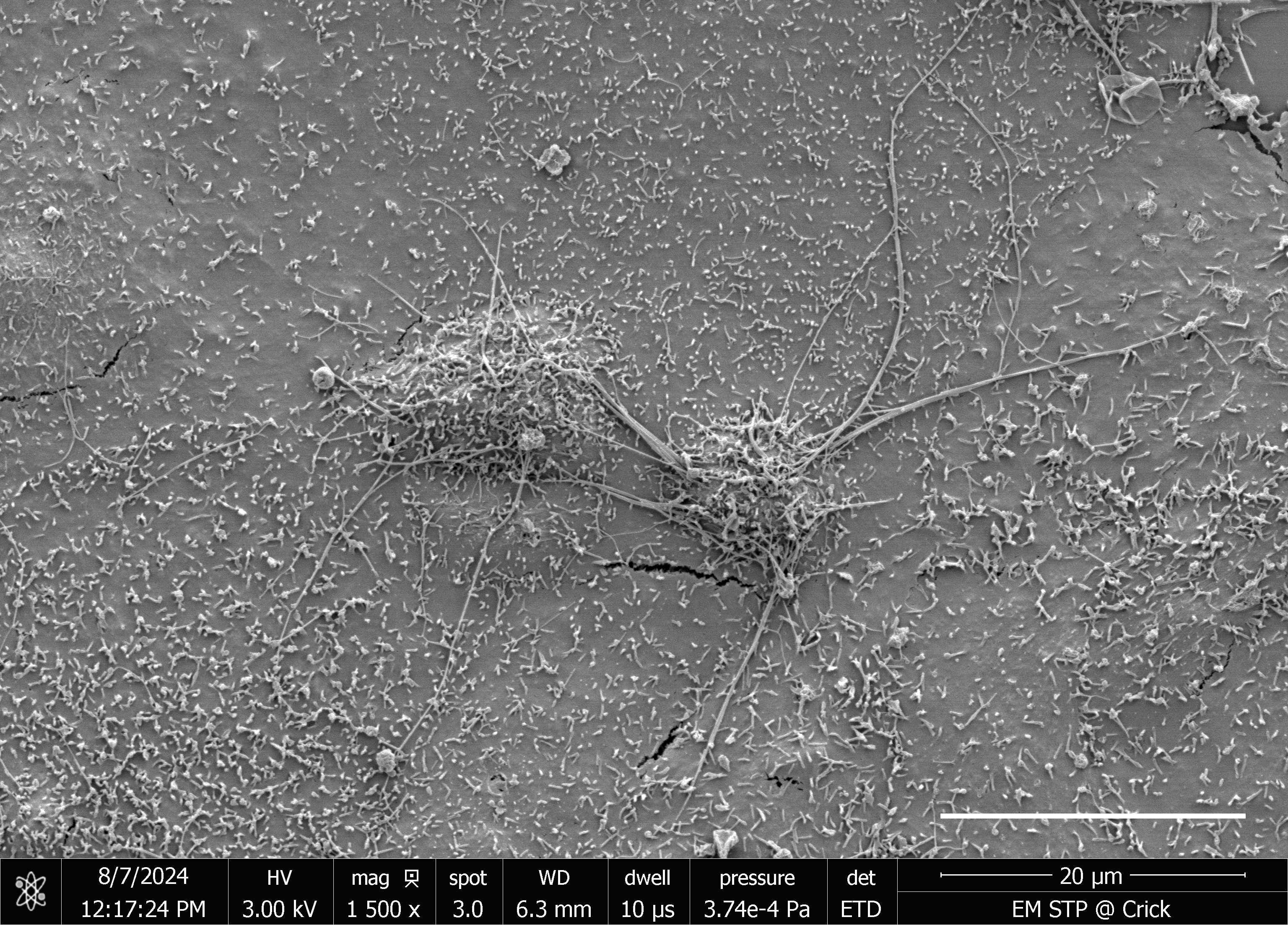

Supplement: Supplementary file 8 — Source data Fig. 6 [file 44319_2025_388_MOESM8_ESM.zip › Figure 6/6B_delta8697_20sb.tif]

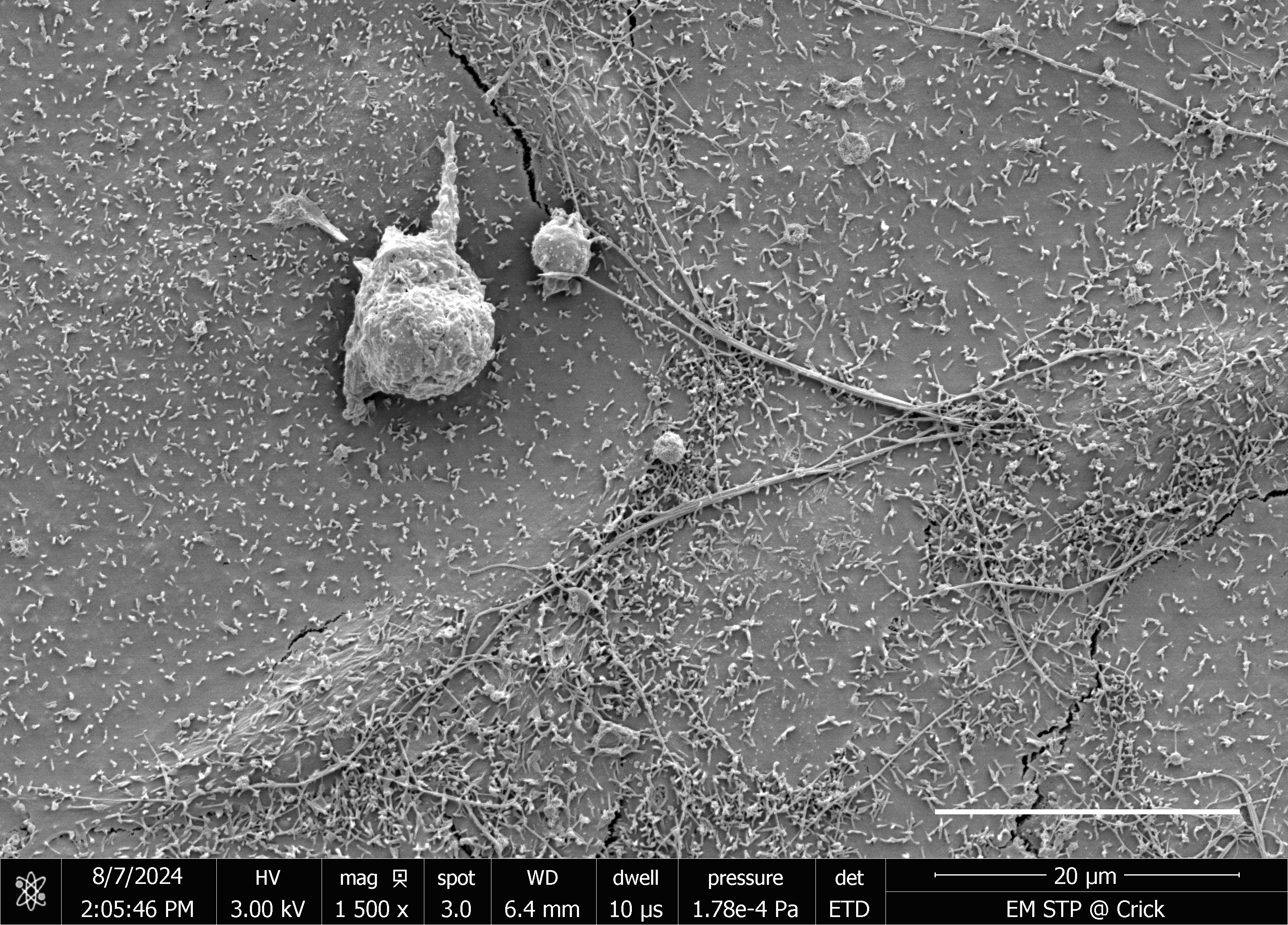

Supplement: Supplementary file 8 — Source data Fig. 6 [file 44319_2025_388_MOESM8_ESM.zip › Figure 6/6B_delta8697_20sb2.tif]

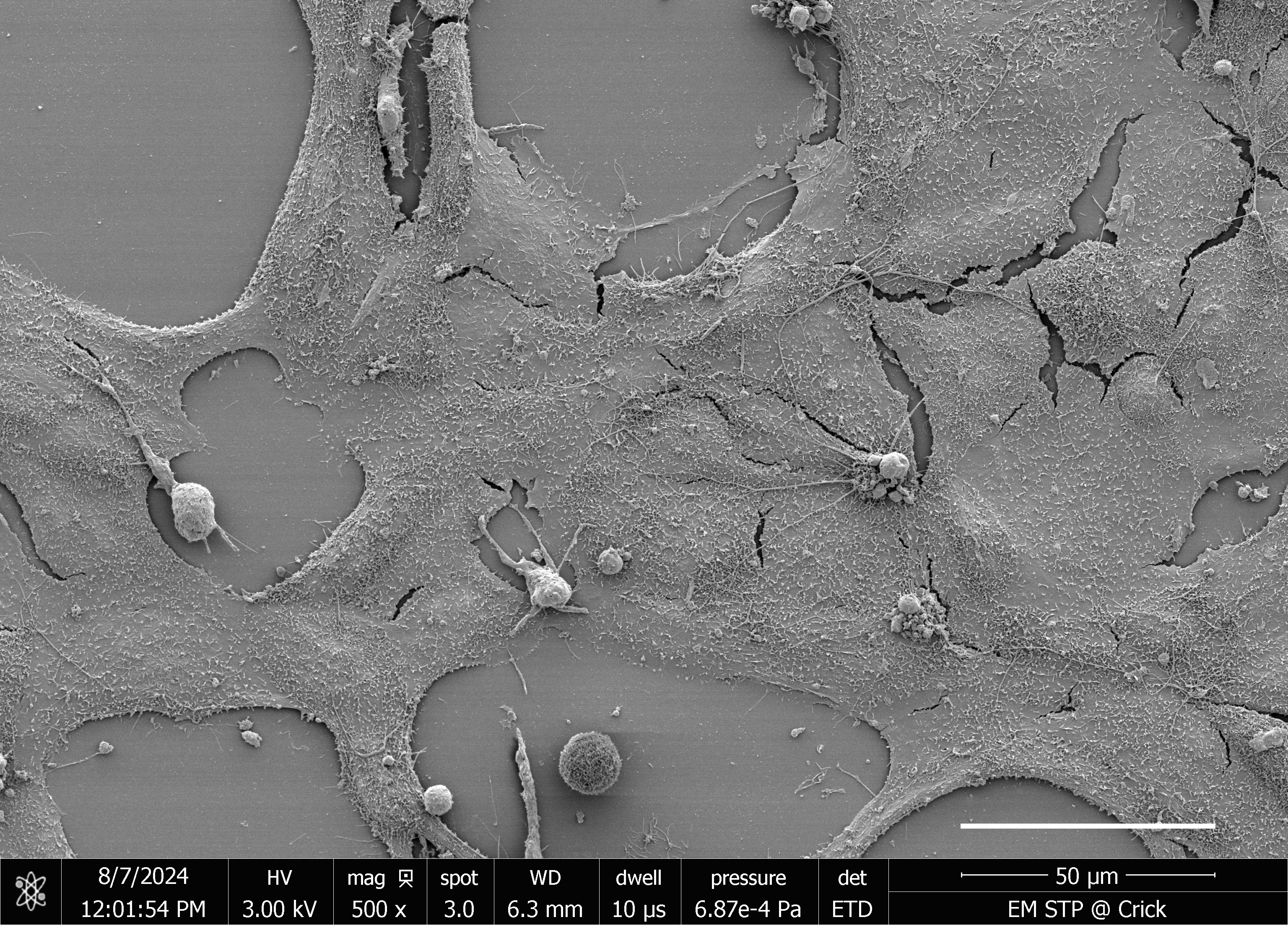

Supplement: Supplementary file 8 — Source data Fig. 6 [file 44319_2025_388_MOESM8_ESM.zip › Figure 6/6B_delta8697_50sb.tif]

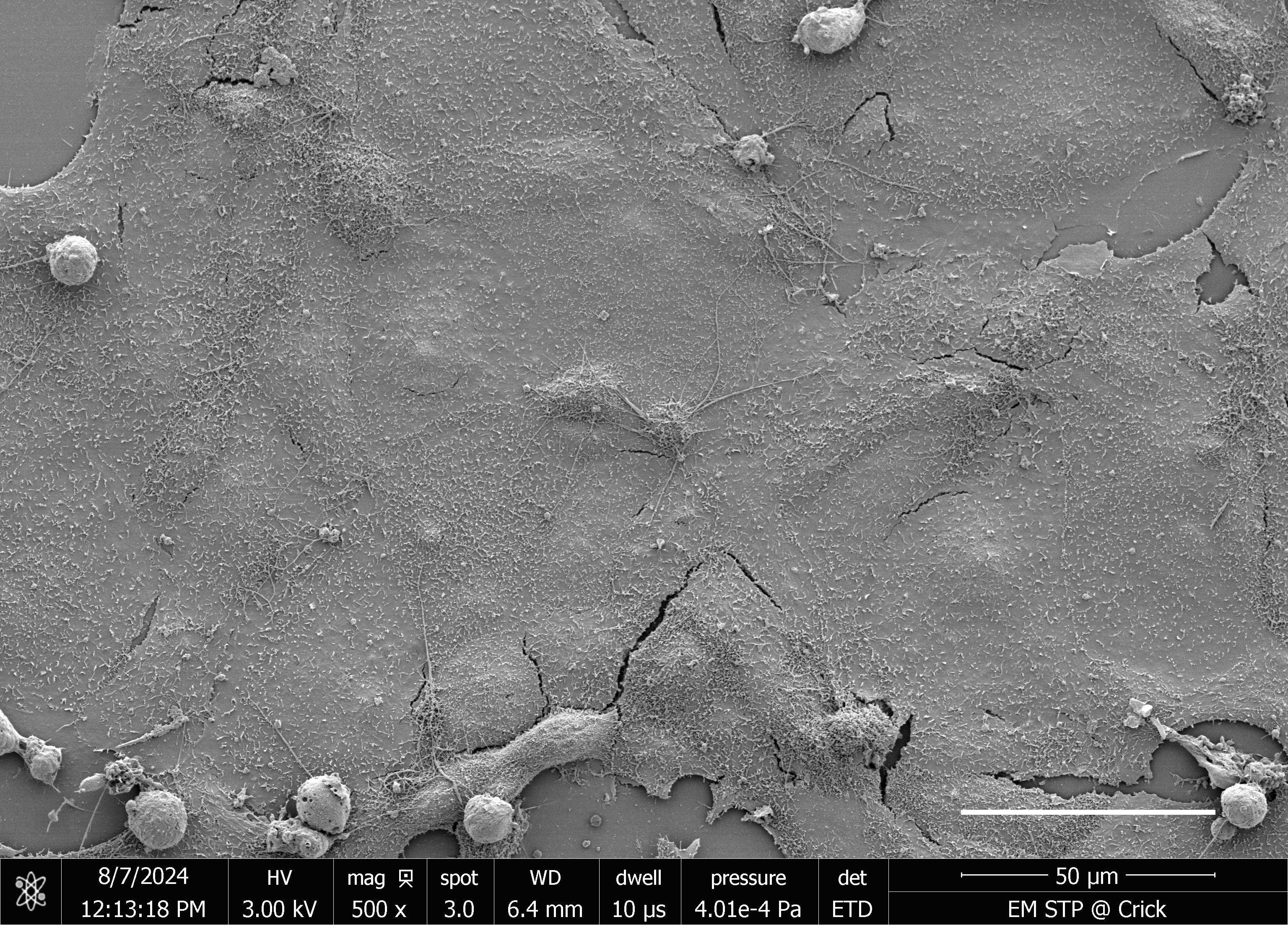

Supplement: Supplementary file 8 — Source data Fig. 6 [file 44319_2025_388_MOESM8_ESM.zip › Figure 6/6B_delta8697_50sb2.tif]

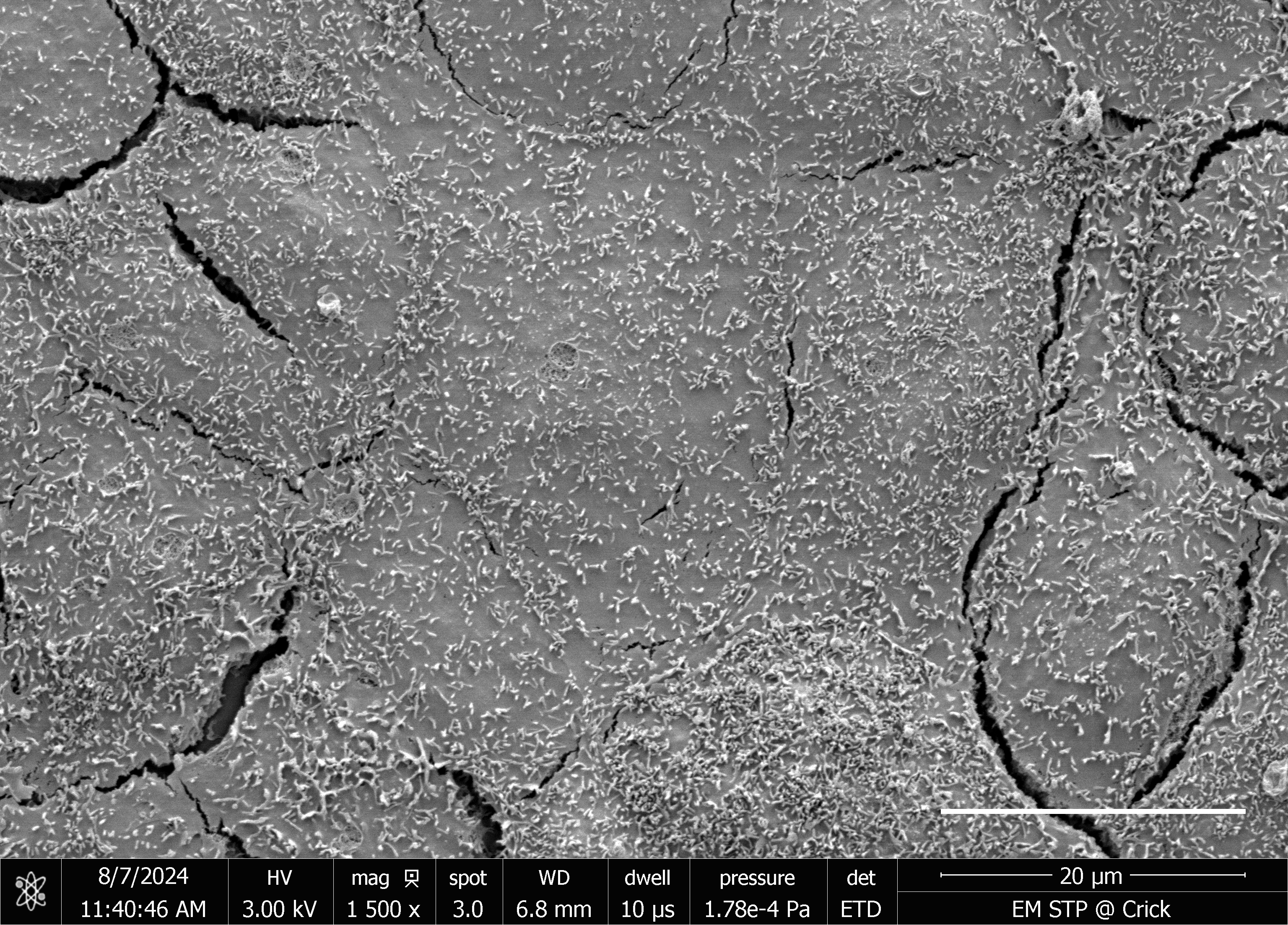

Supplement: Supplementary file 8 — Source data Fig. 6 [file 44319_2025_388_MOESM8_ESM.zip › Figure 6/6B_MOCK_20sb.tif]

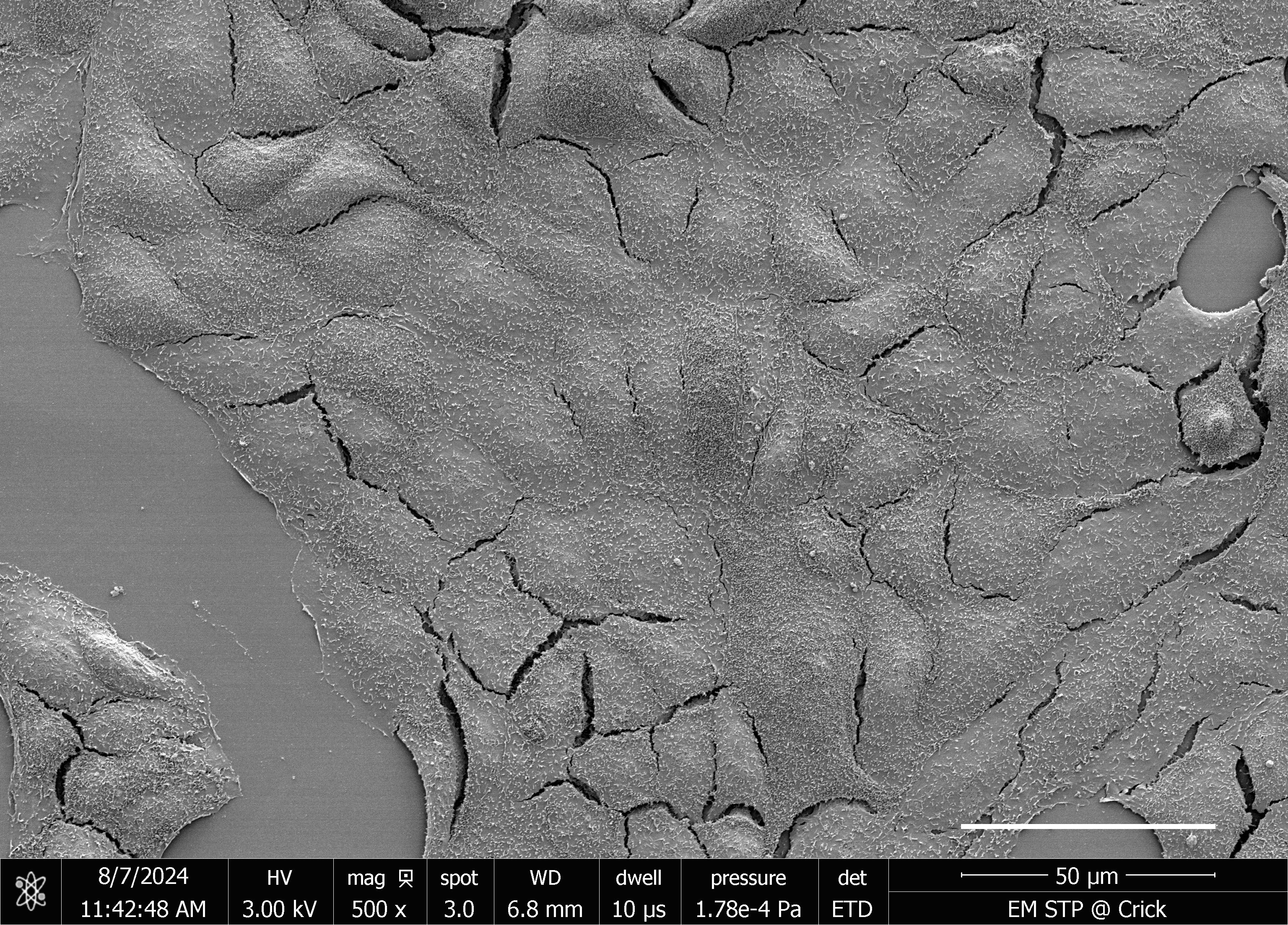

Supplement: Supplementary file 8 — Source data Fig. 6 [file 44319_2025_388_MOESM8_ESM.zip › Figure 6/6B_MOCK_50sb.tif]

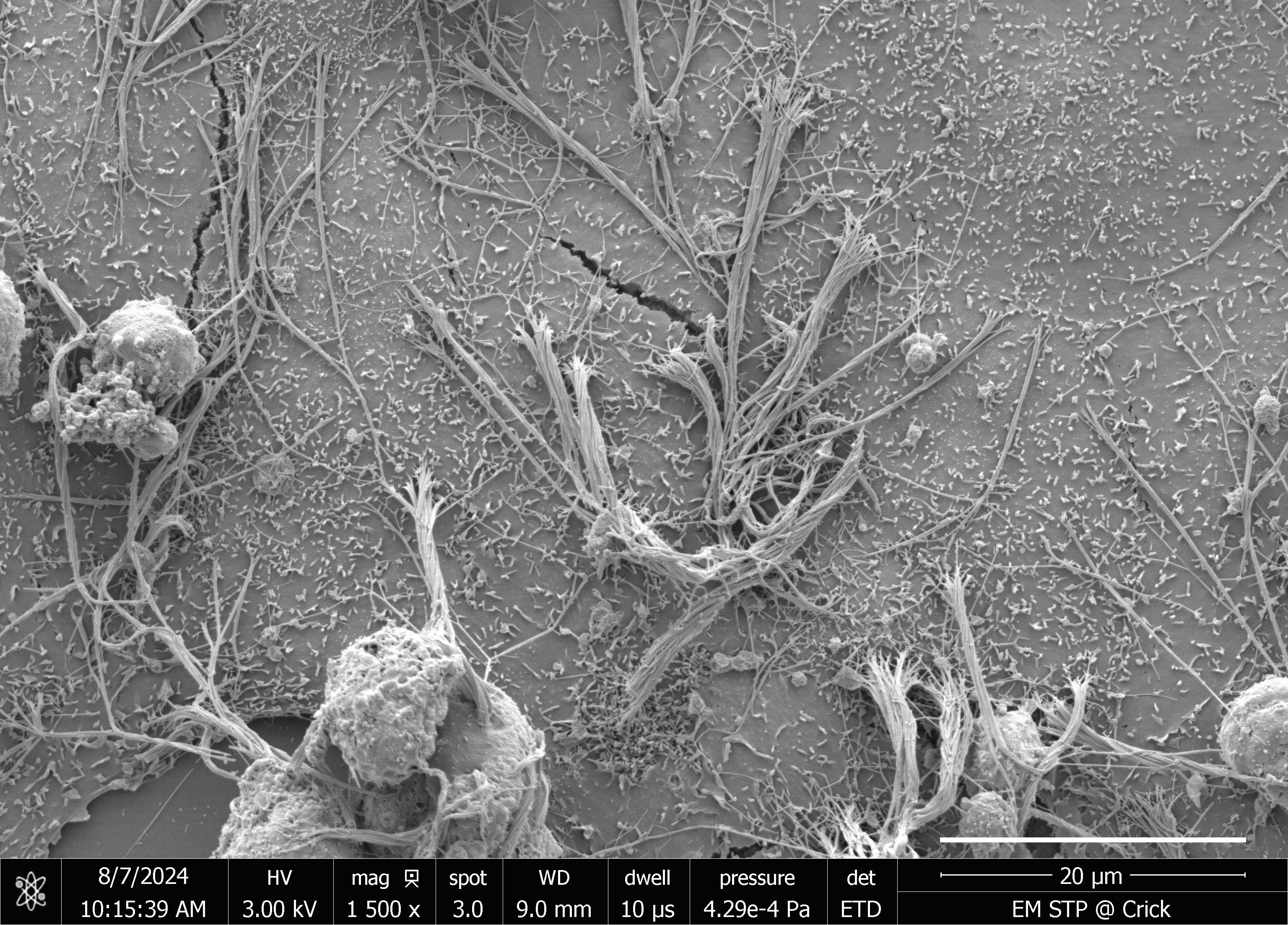

Supplement: Supplementary file 8 — Source data Fig. 6 [file 44319_2025_388_MOESM8_ESM.zip › Figure 6/6B_WT_20sb.tif]

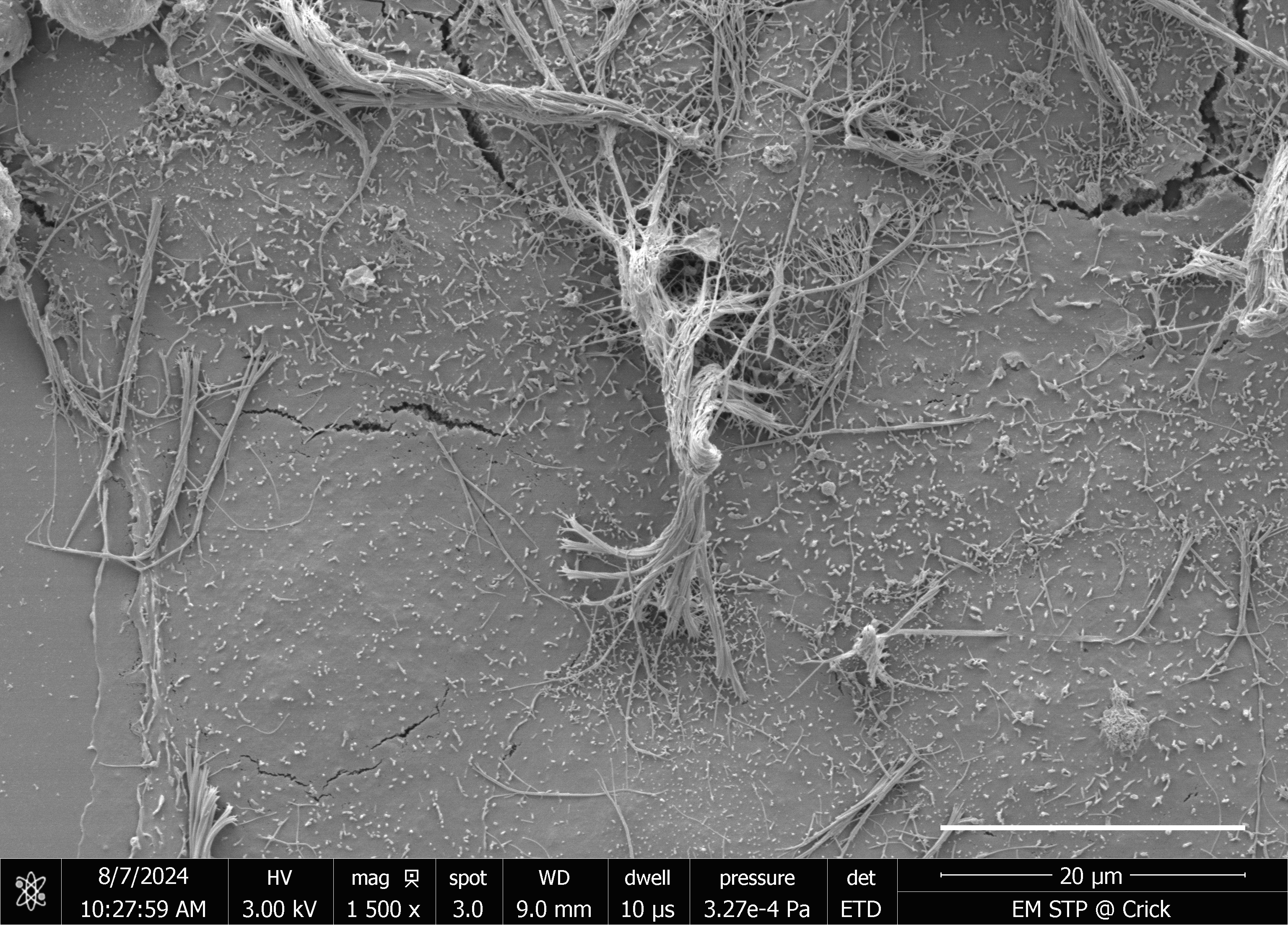

Supplement: Supplementary file 8 — Source data Fig. 6 [file 44319_2025_388_MOESM8_ESM.zip › Figure 6/6B_WT_20sb2.tif]

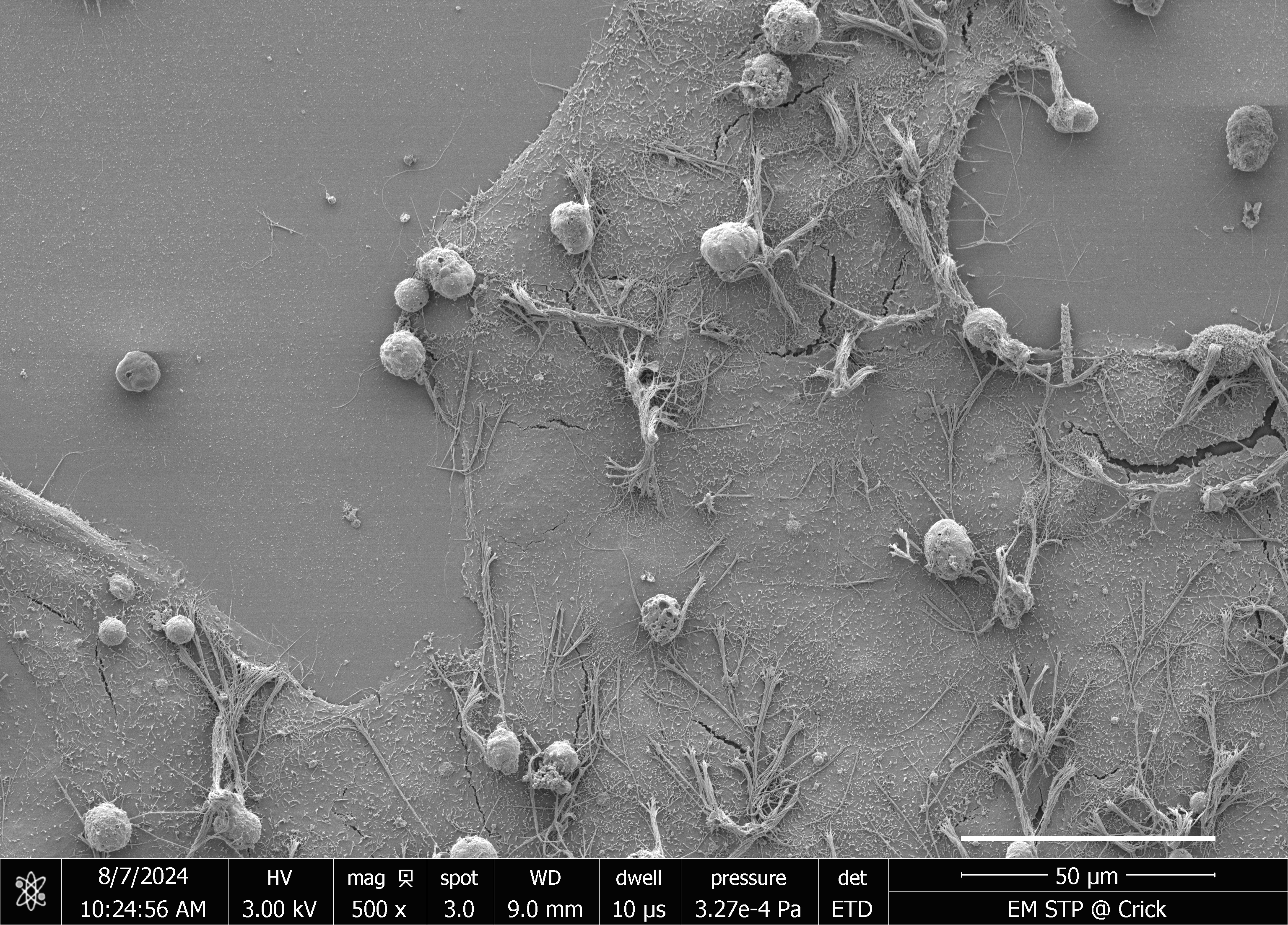

Supplement: Supplementary file 8 — Source data Fig. 6 [file 44319_2025_388_MOESM8_ESM.zip › Figure 6/6B_WT_50sb.tif]
