# Supplementary material for: Caspase cleavage of influenza A virus M2 disrupts M2-LC3 interaction and regulates virion production
Source: EMBO Rep. 2025 Mar 3;26(7):1768–91. doi: 10.1038/s44319-025-00388-7 (PMC11977235; doi:10.1038/s44319-025-00388-7)
Supplement: Supplementary file 5 — Source data Fig. 3 [file 44319_2025_388_MOESM5_ESM.zip › Figure 3/3C_Read me.rtf]

The image files are organised as follows:Figure panel_staining_perm_mutant ee3C_GFPLC3/hoechst/M2_perm_WT/D85A/delta8697
