# Supplementary material for: Caspase cleavage of influenza A virus M2 disrupts M2-LC3 interaction and regulates virion production
Source: EMBO Rep. 2025 Mar 3;26(7):1768–91. doi: 10.1038/s44319-025-00388-7 (PMC11977235; doi:10.1038/s44319-025-00388-7)
Supplement: Supplementary file 9 — Expanded View Figures [file 44319_2025_388_MOESM9_ESM.pdf]

Expanded View Figures

Figure EV1. Extended data supporting "M2 is cleaved at SAVD motif by caspases".

(**A**) Quantification of Fig. 1C. Bars show mean ± SD of $n = 3$ biological replicates. $^{**}P = 0.0065$. Ordinary one-way ANOVA with Bartlett's multiple comparisons. (**B**) Representative immunoblots of lysates of wild type (WT), Δ*CASP6*, and Δ*CASP6* stably expressing Caspase-6. Indicated samples were infected for 16 h with IAV PR8 at an MOI of 10. (**C**) Quantification of (**B**). Bars show mean ± SD of $n = 3$ biological replicates. $^{**}P = 0.0011$. Ordinary one-way ANOVA with Dunnett's multiple comparisons. (**D**) Quantification of Fig. 1D. Bars show mean ± SD of $n = 3$ biological replicates. $^{**}P = 0.0064$. Unpaired *t* test. (**E**) Representative immunoblots of lysates of THP-1 cells infected with IAV PR8 for 24 h. Indicated samples were treated with DMSO as a control, or 50 μM pan-caspase inhibitor (Z-VAD-FMK), caspase-3 inhibitor (Z-DEVD-FMK), and caspase-8 inhibitor (Z-IETD-FMK) as indicated. (**F**) Quantification of (**E**). Bars show mean ± SD of $n = 3$ biological replicates. $^{**}P = 0.0041$. Ordinary one-way ANOVA with Dunnett's multiple comparisons. (**G**) Multiple sequence alignment of amino acids 82-97 in IAV M2. IAV PR8 $M2^{WT}$, $M2^{D85A}$, and $M2^{\Delta 86-97}$ sequences were produced with Sanger sequencing and aligned to Influenza A virus (A/Puerto Rico/8/34(H1N1)) segment 7 (Schickli et al, 2001). (**H**) Quantification of Fig. 1F. Graph shows data points for $n = 2$ biological replicates.

© The Author(s)

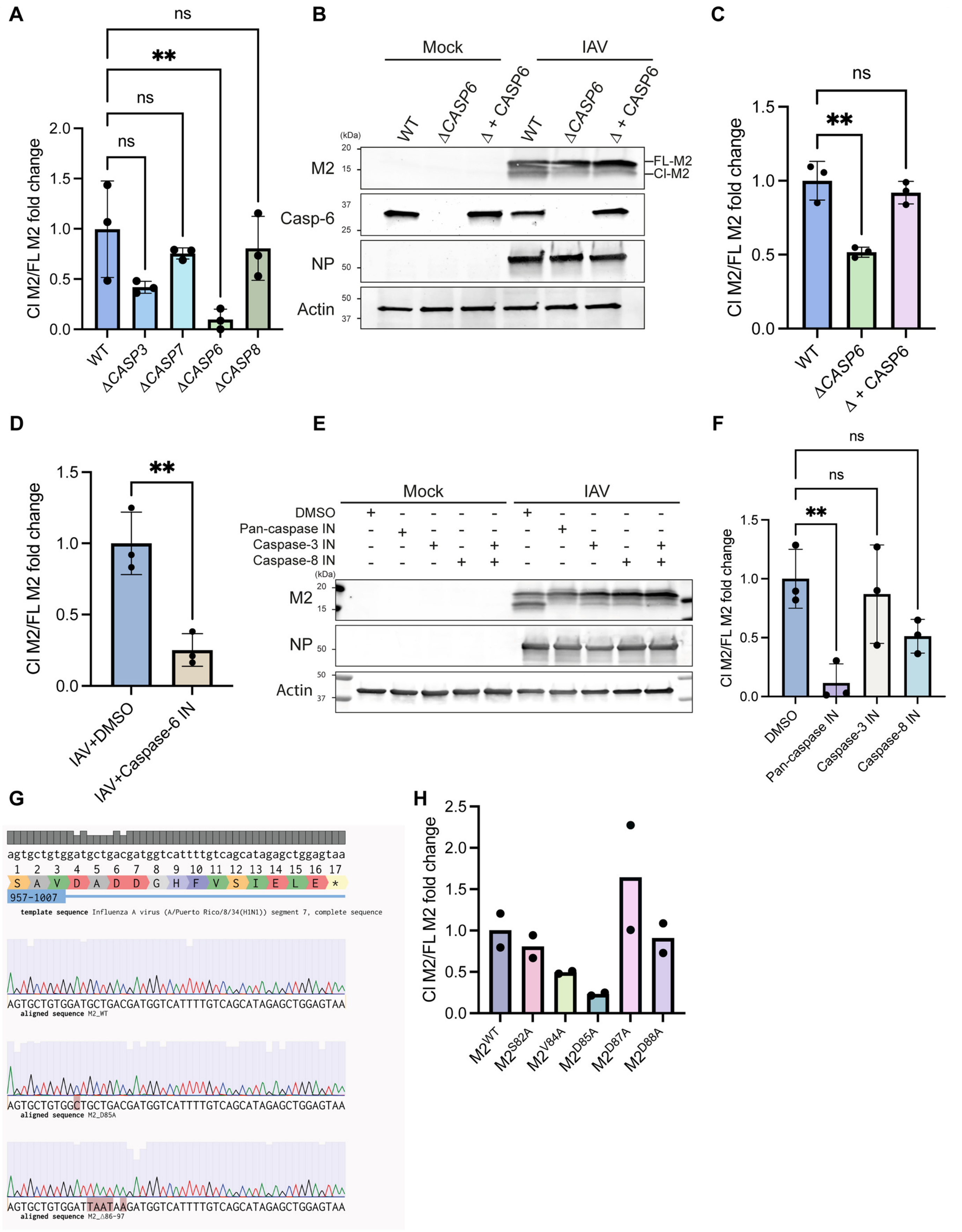
A
ns
**
ns
ns
CI M2/FL M2 fold change
2.0
1.5
1.0
0.5
0.0
WT
ΔCASP3
ΔCASP7
ΔCASP6
ΔCASP8
B
Mock
IAV
WT
ΔCASP6
Δ + CASP6
(kDa)
M2
Casp-6
NP
Actin
FL-M2
Cl-M2
C
ns
**
CI M2/FL M2 fold change
WT
ΔCASP6
Δ + CASP6
D
**
CI M2/FL M2 fold change
IAV+DMSO
IAV+Caspase-6 IN
E
Mock
IAV
DMSO
Pan-caspase IN
Caspase-3 IN
Caspase-8 IN
M2
NP
Actin
F
ns
ns
**
CI M2/FL M2 fold change
DMSO
Pan-caspase IN
Caspase-3 IN
Caspase-8 IN
G
agtgctgtggatgctgacgatggtcattttgtcagcatagagctggagtaa
1 2 3 4 5 6 7 8 9 10 11 12 13 14 15 16 17
S A V D A D D G H F V S I E L E *
957-1007
template sequence Influenza A virus (A/Puerto Rico/8/34(H1N1)) segment 7, complete sequence
AGTGCTGTGGATGCTGACGATGGTCATTTTGTCAGCATAGAGCTGGAGTAA
aligned sequence M2_WT
AGTGCTGTGGCTGCTGACGATGGTCATTTTGTCAGCATAGAGCTGGAGTAA
aligned sequence M2_D85A
AGTGCTGTGGATTAATAAGATGGTCATTTTGTCAGCATAGAGCTGGAGTAA
aligned sequence M2_Δ86-97
H
CI M2/FL M2 fold change
2.5
2.0
1.5
1.0
0.5
0.0
M2WT
M2S82A
M2V84A
M2D85A
M2D87A
M2D88A

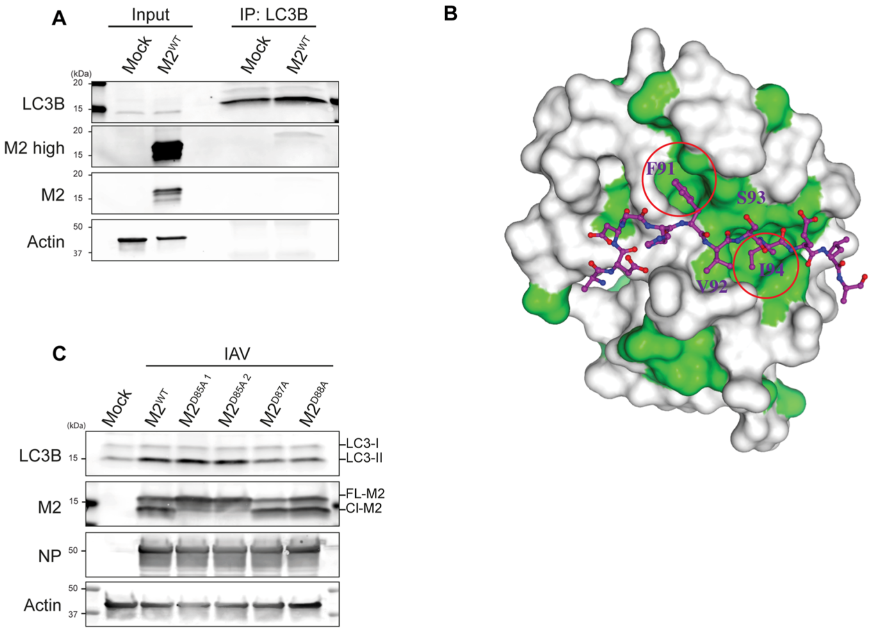

Figure EV2. Extended data supporting "M2 cleavage disrupts M2–LC3 interaction".

(**A**) Immunoprecipitation of endogenous LC3B from A549 cells analyzed by western blotting. Indicated samples were infected for 24 h with IAV PR8 WT, or mutant strains, with an MOI of 10. (**B**) Surface representation model of M2–LC3B LIR complex. Hydrophobic residues of LC3B are colored green. Red circles indicate hydrophobic pockets of LC3B for LIR interaction. (**C**) Representative immunoblots of lysates of THP-1 cells infected with IAV $M2^{WT}$, $M2^{D85A}$, $M2^{D87A}$and $M2^{D88A}$ mutants for 24 h with an MOI of 10.

 © The Author(s)

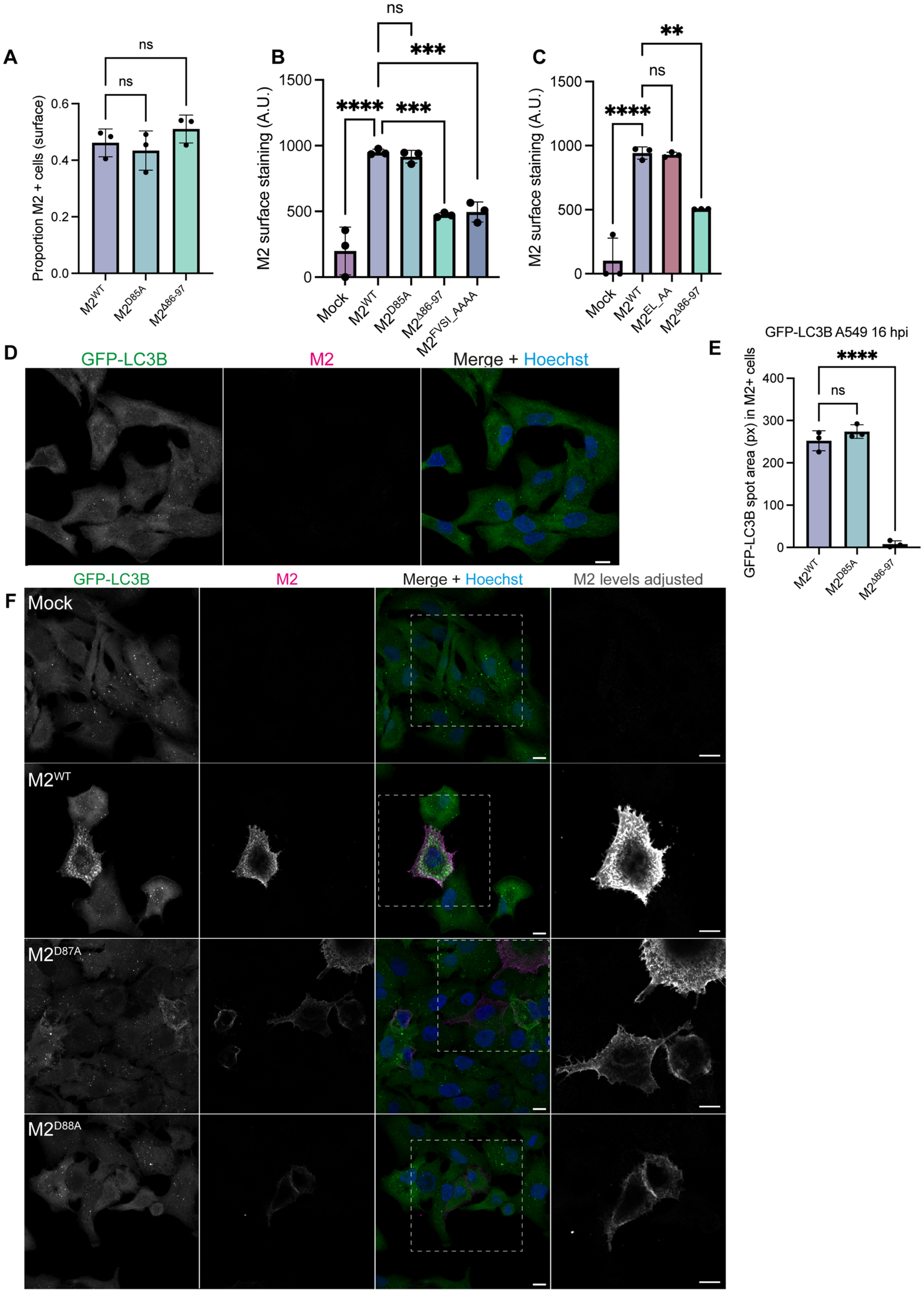
A
B
C
D
E
F
ns
****
***
**
Proportion M2 + cells (surface)
M2 surface staining (A.U.)
GFP-LC3B A549 16 hpi
GFP-LC3B spot area (px) in M2+ cells
Mock
M2WT
M2D85A
M2Δ86-97
M2FVSI_AAAA
M2EL_AA
GFP-LC3B
M2
Merge + Hoechst
M2 levels adjusted
M2D87A
M2D88A

Figure EV3. Extended data supporting "Cleaved M2 is incorporated into virions at a lower rate and exhibits a decreased titer".

(**A**) Quantification of the proportion of M2-positive cells in surface-stained THP-1 cells. Samples were infected with IAV PR8 $M2^{WT}$, $M2^{D85A}$, and $M2^{\Delta 86\text{-}97}$ for 8 h with an MOI of 1. Bars show mean ± SD of $n = 3$ technical replicates. Ordinary one-way ANOVA with Dunnett's multiple comparisons. (**B**) Quantification of M2 intensity in surface-stained THP-1 cells. Samples were infected with IAV PR8 $M2^{WT}$, $M2^{D85A}$, $M2^{\Delta 86\text{-}97}$, and $M2^{FVSI\_AAAA}$ for 8 h with an MOI of 1. Bars show mean ± SD of $n = 3$ technical replicates. ****$P < 0.0001$, ***: $M2^{WT}$ vs. $M2^{\Delta 86\text{-}97}$ $P = 0.0003$ and $M2^{WT}$ vs. $M2^{FVSI\_AAAA}$ $P = 0.0004$. Ordinary one-way ANOVA with Dunnett's multiple comparisons. (**C**) Quantification of M2 intensity in surface-stained THP-1 cells. Samples were infected with IAV PR8 $M2^{WT}$, $M2^{EL\_AA}$, and $M2^{\Delta 86\text{-}97}$ for 8 h with an MOI of 1. Bars show mean ± SD of $n = 3$ technical replicates. ****$P < 0.0001$, **$P = 0.0010$. Ordinary one-way ANOVA with Dunnett's multiple comparisons. (**D**) Representative images of GFP-LC3B A549 cells mock-infected from the experiment shown in Fig. 3C. Images show GFP-LC3B (green), M2 (magenta), and Hoechst (blue). Scale bar represents 10 µm. (**E**) Quantification of GFP-LC3B spot area in pixels in M2-positive GFP-LC3B A549 cells. Samples were infected with IAV PR8 $M2^{WT}$, $M2^{D85A}$, and $M2^{\Delta 86\text{-}97}$ for 16 h with an MOI of 10. Bars show mean ± SD of $n = 3$ technical replicates. ****$P < 0.0001$. Ordinary one-way ANOVA with Dunnett's multiple comparisons. (**F**) Representative images of surface-stained GFP-LC3B A549 cells that have either been mock-treated or infected with IAV PR8 $M2^{WT}$, $M2^{D87A}$, and $M2^{D88A}$ mutants for 16 h with an MOI of 10. Images show GFP-LC3B (green), M2 (magenta), and Hoechst (blue). Dashed box highlights the area of crop. M2 images were also adjusted to the same levels across infections to illustrate differences in intensity in M2 surface staining. Scale bar represents 10 µm.

 © The Author(s)

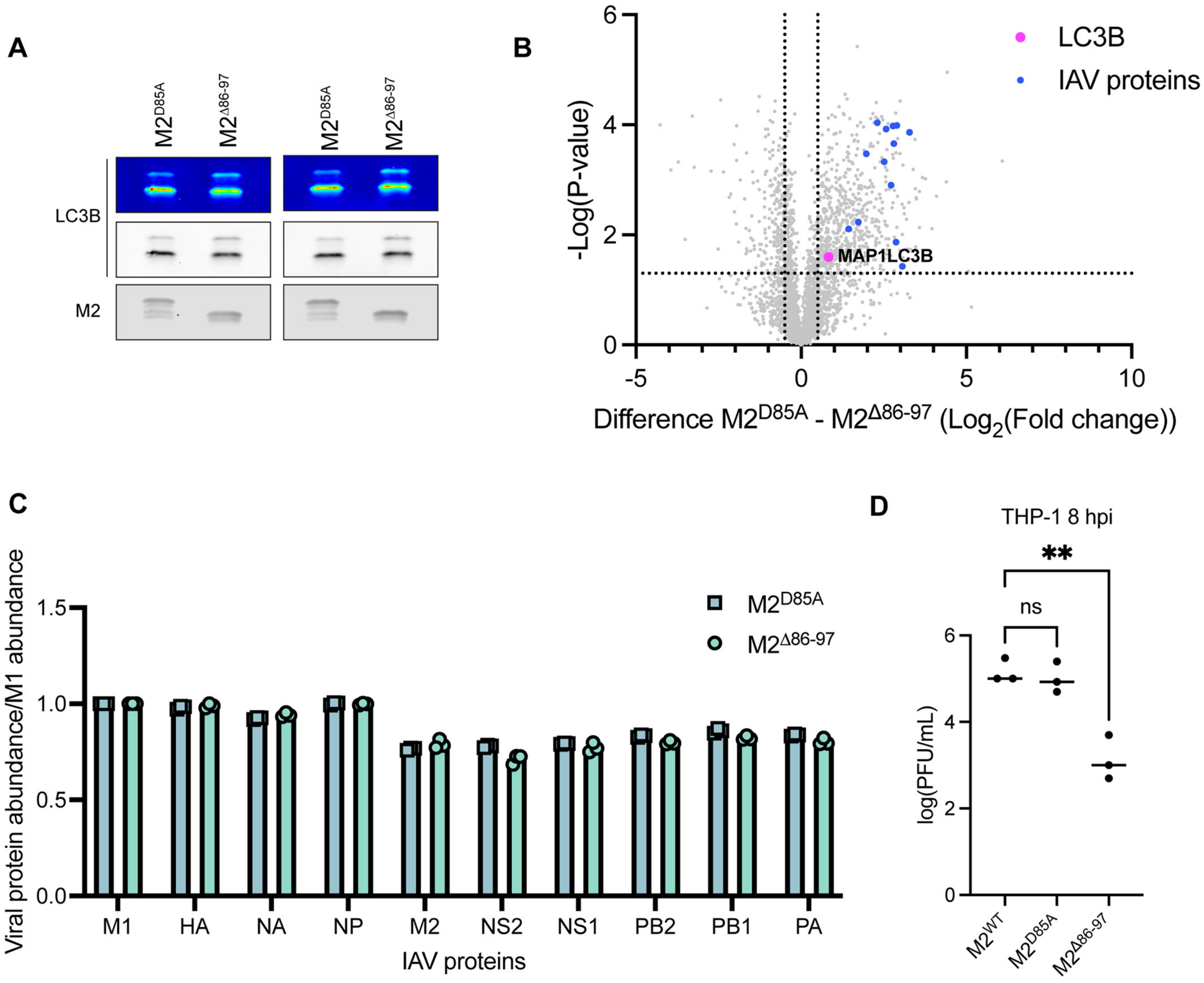

Figure EV4. Extended data supporting "M2 cleavage reduces M2 incorporation into virions" and "IAV M2$^{\Delta86\text{-}97}$ is attenuated".

(**A**) Biological replicates of immunoblots in Fig. 4E showing lysate of infectious particles following purification. Cells were infected with IAV PR8 M2^{D85A} or M2$^{\Delta86\text{-}97}$ for 48 h. The supernatant was then collected and purified. (**B**) Volcano plot showing the difference in log_2(fold change) of the abundance of proteins expressed in M2^{D85A} purified virions when comparing them to M2$^{\Delta86\text{-}97}$ purified virions from 3 biological replicates. MAP1LC3B is shown as a pink dot, and Influenza A proteins are shown as blue dots. Dashed lines represent a difference in log_2(fold change) of more than 0.5 or less than -0.5, and -Log(P-value) of 1.3). Unpaired *t* test. (**C**) Abundance of viral proteins was calculated through normalization to M1 abundance and compared between M2^{D85A} and M2$^{\Delta86\text{-}97}$ abundance. Bars show mean ± SD of $n = 3$ biological replicates. (**D**) Plaque assay quantification to assess IAV titer following THP-1 infection. The supernatant of THP-1 cells infected with IAV PR8 M2^{WT}, M2^{D85A}, and M2$^{\Delta86\text{-}97}$ mutants was collected after 8 h. Plaque assays were performed for 48 h. Bars show mean ± SD of $n = 3$ biological replicates. $^{**}P = 0.0013$. Graph shows data as Y=log(Y). Ordinary one-way ANOVA with Dunnett's multiple comparisons.

© The Author(s)

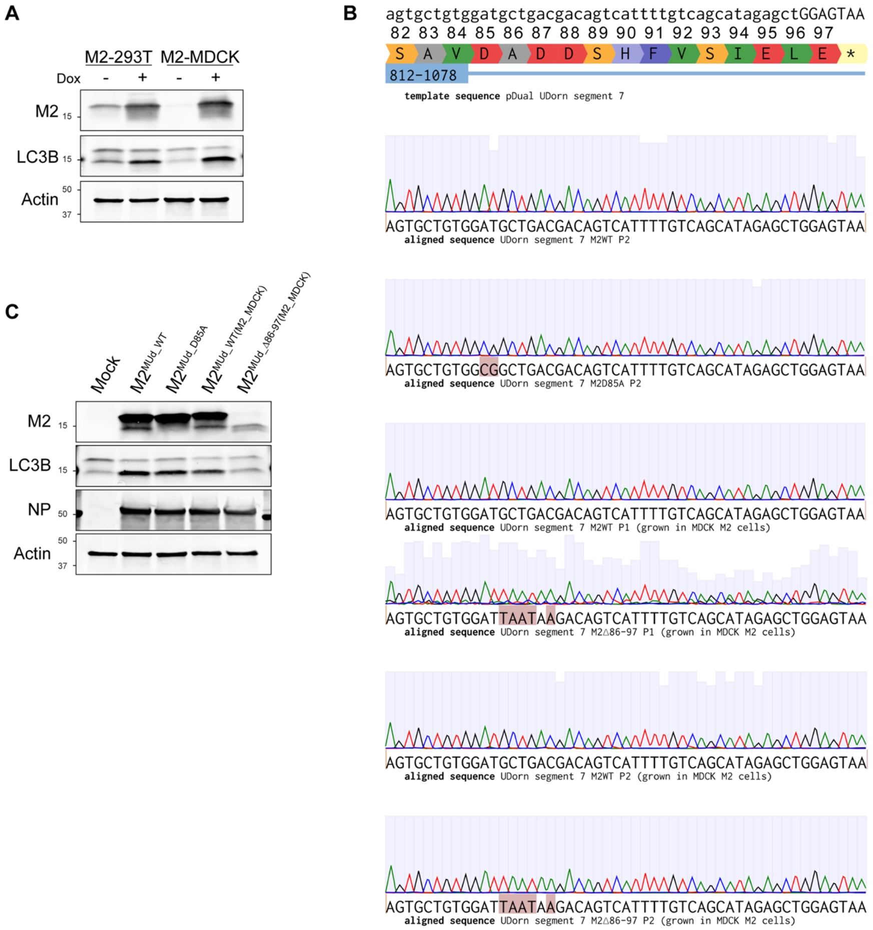

Figure EV5. Extended data supporting "IAV M2$^{\Delta 86\text{-}97}$ mutant in a filamentous background is defective in filamentous budding".

(**A**) Immunoblot of lysates of HEK293T and MDCK cells expressing a doxycycline-inducible promoter. Indicated cells were treated with doxycycline for 24 h. (**B**) Multiple sequence alignment of amino acids 82-97 in IAV M2. IAV MUd M2$^{\text{MUd-WT}}$, M2$^{\text{MUd-D85A}}$, M2$^{\text{MUd-WT(M2_MDCK)}}$ (grown in HEK293T and MDCK cells expressing M2 under a doxycycline-inducible promoter), and M2$^{\text{MUd-}\Delta 86\text{-}97\text{(M2_MDCK)}}$ (grown in HEK293T and MDCK cells expressing M2 under a doxycycline-inducible promoter). Sequencing of two viral passages is shown for M2$^{\text{MUd-WT(M2_MDCK)}}$ and M2$^{\text{MUd-}\Delta 86\text{-}97\text{(M2_MDCK)}}$ to verify amino acid substitutions leading to truncation have not been reversed. Sequences were produced with Sanger sequencing and aligned to pDual A/Udorn/307/1972 H3N2 segment 7. (**C**) Immunoblot of lysates of MDCK cells infected with IAV MUd M2$^{\text{MUd-WT}}$, M2$^{\text{MUd-D85A}}$, M2$^{\text{MUd-WT(M2_MDCK)}}$, and M2$^{\text{MUd-}\Delta 86\text{-}97\text{(M2_MDCK)}}$ for 24 h.

© The Author(s)
